# Exploring temporal trends and burden of traumatic shoulder dislocation: a global perspective

**DOI:** 10.3389/fpubh.2024.1346957

**Published:** 2024-02-29

**Authors:** Cheng Chen, Tianbao Ye, Jiantao Jiang, Wenbao He, Jiang Xia, Yunfeng Yang

**Affiliations:** ^1^Department of Orthopedics, Tongji Hospital, School of Medicine, Tongji University, Shanghai, China; ^2^Department of Cardiology, Shanghai Sixth People’s Hospital Affiliated to Shanghai Jiao Tong University School of Medicine, Shanghai, China; ^3^Department of Orthopedic Surgery, Shaoxing Shangyu Traditional Chinese Medicine Hospital, Zhejiang, China; ^4^Department of Orthopaedics, Ruijin Hospital, Shanghai Jiao Tong University School of Medicine, Shanghai, China

**Keywords:** traumatic shoulder dislocation, Global Burden of Disease, epidemiology, incidence, years lived with disability

## Abstract

**Objective:**

To explore the geographical and temporal trends of traumatic shoulder dislocation, describe the association between the social and demographic factors and the health burden due to traumatic shoulder dislocation, and further investigate its causes.

**Methods:**

Data on traumatic shoulder dislocation was collected from the Global Burden of Disease 2019, spanning the years 1990 to 2019. The epidemiology and disease burden were examined at global, regional, and national levels. Additionally, the age and gender patterns were analyzed, followed by an investigation into the primary causes. Lastly, the study studied the correlation between age-standardized rates and the socio-demographic index (SDI).

**Results:**

Over a span of 30 years, both the crude and age-standardized rates of incidence and years lived with disability (YLDs) rates for all genders displayed a slight fluctuating downward trend. The incidence and YLDs rates in males were consistently higher than those in females. The study analyzed both incidence and YLDs rates of the global, regional, and national of traumatic shoulder dislocations from 1990 to 2019, as well as the temporal trends. Among males, the highest incidence rate was observed in young adulthood, while females exhibited the highest incidence rate in old age. This pattern was mirrored in the YLDs rate. Falls were identified as the main cause contributing to the disease burden related to traumatic shoulder dislocations. Moreover, a positive correlation was found between the age-standardized rates and SDI.

**Conclusion:**

The disease burden of traumatic shoulder dislocation has not significantly decreased from 1990 to 2019. The incidence and YLD rates are associated with age, gender, and SDI. A thorough examination of the disease burden contributes to the efficient allocation and utilization of resources, as well as the development of targeted and effective intervention strategies.

## Introduction

Shoulder dislocation is a common site for joint dislocations (1–3). Hovelius (4) reported that up to 1.7% of adults in Sweden have experienced shoulder dislocation. The treatment for traumatic shoulder dislocation includes conservative treatment and surgical treatment. The main complications of traumatic shoulder dislocation are recurrent dislocation of the shoulder joint and arthritis (5). Studies have shown a recurrence rate of shoulder dislocation was 33% (6). A meta-analysis showed that among patients who received non-surgical treatment after their first traumatic shoulder dislocation, 53% experienced recurrent shoulder dislocations (7). In the average 15 years follow-up observation, 10.7% of patients developed moderate to severe arthritis, and if mild arthritis was included, the prevalence rate reached 44.6% (8). As the course of shoulder arthritis progresses, pain and reduced range of motion become important factors affecting patients’ daily work and life. As one of the most frequently used joints in the human body, the shoulder joint plays a crucial role in daily activities and quality of life. It is important to note that the high-risk population for shoulder dislocation is young patients (9), thus the impact should be carefully evaluated. Additionally, the personal and economic costs of shoulder dislocation are burdensome. Research showed that the average direct medical cost of treating shoulder dislocation was $612 (1), and the total expenditure (including indirect economic expenses) will be even higher.

Literature reports showed significant variation in the incidence rate, ranging from 8.2 to 26.2 per 100,000 (1, 9–15). Simonet et al. (10) reported an incidence rate of 8.2 per 100,000 for traumatic primary anterior shoulder dislocation in Olmsted County, Minnesota from 1970 to 1979. Kroner et al. (11) reported an overall annual incidence rate of 17 per 100,000 in Aarhus Borough, Denmark from 1980 to 1984. Nordqvist and Petersson (12) reported an incidence rate of approximately 23.9 per 100,000 for shoulder dislocation in Malmo, Sweden in 1987. In Taiwan, the annual incidence rate of shoulder dislocation from 2000 to 2005 was approximately 15.3 per 100,000 (1). According to reported data, the annual rate of closed reduction for anterior shoulder dislocation in Ontario, Canada, from 2002 to 2010, was 23.1 per 100,000 (13). Based on data from Oslo, Norway, the incidence rate for primary shoulder dislocation was approximately 26.2 per 100,000 in 2009 (14). A study based on the National Electronic Injury Surveillance System reported an incidence rate of 23.9 per 100,000 for shoulder dislocation from 2002 to 2006 in USA (9). An updated analysis of data from the National Electronic Injury Surveillance System by Patrick et al. (15) showed an incidence rate of 24.0 per 100,000 from 2012 to 2021 in USA.

The existing literature on the epidemiology of shoulder dislocation is limited to specific regions and has a limited timeframe. It also lacks a detailed analysis of the causes of injury and social demographic factors. Therefore, it is necessary to examine the epidemiology and disease burden of shoulder dislocation from a broader geographical and temporal perspective. This is important for the allocation and planning of healthcare resources, the formulation of public health policies, and the establishment of future development goals.

The aim of this study is to explore the geographical and temporal trends of traumatic shoulder dislocation, describe the association between the social and demographic factors and the health burden due to traumatic shoulder dislocation, and further investigate its causes.

## Methods

The Global Burden of Disease (GBD) Study 2019 is a comprehensive scientific research conducted by the Institute for Health Metrics and Evaluation (16). Its main objective is to analyze the burden of disease, health loss, and risk factors. The data acquisition process is standardized and controllable, and the analysis is conducted rigorously and scientifically. The data collected by GBD is gaining recognition, and research articles utilizing GBD data are being published in high-quality journals (17–20). Significantly, GBD 2019 offers a unified data source obtained through a standardized estimation method, allowing for comparisons of epidemiology and disease burden across countries and territories globally. This database holds great importance as it provides evidence for identifying epidemiological patterns and evaluating health systems, thereby optimizing investments in research and development.

Three hundred sixty-nine causes of death or injuries and 87 risk factors across 204 countries and territories from 1990 to 2019 are evaluated by GBD 2019 (16). GBD 2019 follows the guidelines for Accurate and Transparent Health Estimation Reporting for Population Health Research. The GBD study used a model framework to calculate the burden caused by various diseases and injuries. Briefly, the process was as follows (16): firstly, raw data was obtained from sources such as community surveys, national surveys, monitoring data, outpatient and inpatient data, etc. Next, GBD adjusted the raw data by adding covariates and adjusting for factors such as cause of death and population statistics. The adjusted data was then analyzed and estimated using the Bayesian meta-regression tool DisMod-MR 2.1, as well as other modeling strategies. Subsequently, estimates of injury severity distribution, disability weights, and comorbidity corrections were performed to calculate the final burden estimates for each disease and injury by age, sex, year, and location. Data is accessed via the Global Health Data Exchange query tool.^1^

The socio-demographic index (SDI) is used to assess the development status correlated with health outcomes. SDI includes three indicators: total fertility rate of the population under 25 years old, average educational attainment of the population aged 15 and above, and *per capita* lag-distributed income. The range of SDI is from 0 to 1. A higher score indicates a higher level of theoretical development related to health. SDI serves as a fundamental tool that promotes evidence-based decision-making, policy formulation, and resource allocation. Its comprehensive nature enhances our understanding of the complex interplay between socio-economic and demographic factors, contributing to informed strategies aimed at achieving equitable and sustainable development worldwide. SDI, as a tool, has been widely recognized for its reliability and effectiveness (21–25). SDI data is publicly available via the Global Health Data Exchange Query Tool.^2^

Two hundred four countries and territories are divided into 21 GBD regions in GBD 2019.^3^ Population data is accessible to the public via a website.^4^

In this study, we collected data on traumatic shoulder dislocation from 1990 to 2019 from GBD 2019, including estimates of crude and standardized incidence and years lived with disability (YLDs), as well as a 95% uncertainty interval (UI). YLDs refers to the years of healthy life lost due to disability caused by traumatic shoulder dislocation, which is a measurement indicator of quality of life. The UI refers to the actual probability distribution around the true parameter values, obtained by sampling 1,000 times during the estimation process. The 95% UI was calculated according to the 2.5th and 97.5th ranked draws (16).

### Statistical analysis

The epidemiology and burden of traumatic shoulder dislocation, including age, gender, and mechanism, were demonstrated at the global, regional, and national levels. The estimated annual percent change (EAPC) with a 95% confidence interval (CI) from 1990 to 2019 was calculated (26). A positive EAPC indicates an increasing trend, while a negative EAPC indicates a decreasing trend. Next, the age and gender patterns of traumatic shoulder dislocation were studied. Subsequently, main causes of traumatic shoulder dislocation were investigated. Finally, the Pearson correlation between age-standardized rates and SDI was calculated. *p* < 0.05 indicated a statistical significance. All statistical analyses and data visualizations were performed using R (version 4.2.2).

## Results

### The burden of traumatic shoulder dislocation at the global level

**Figure 1:**
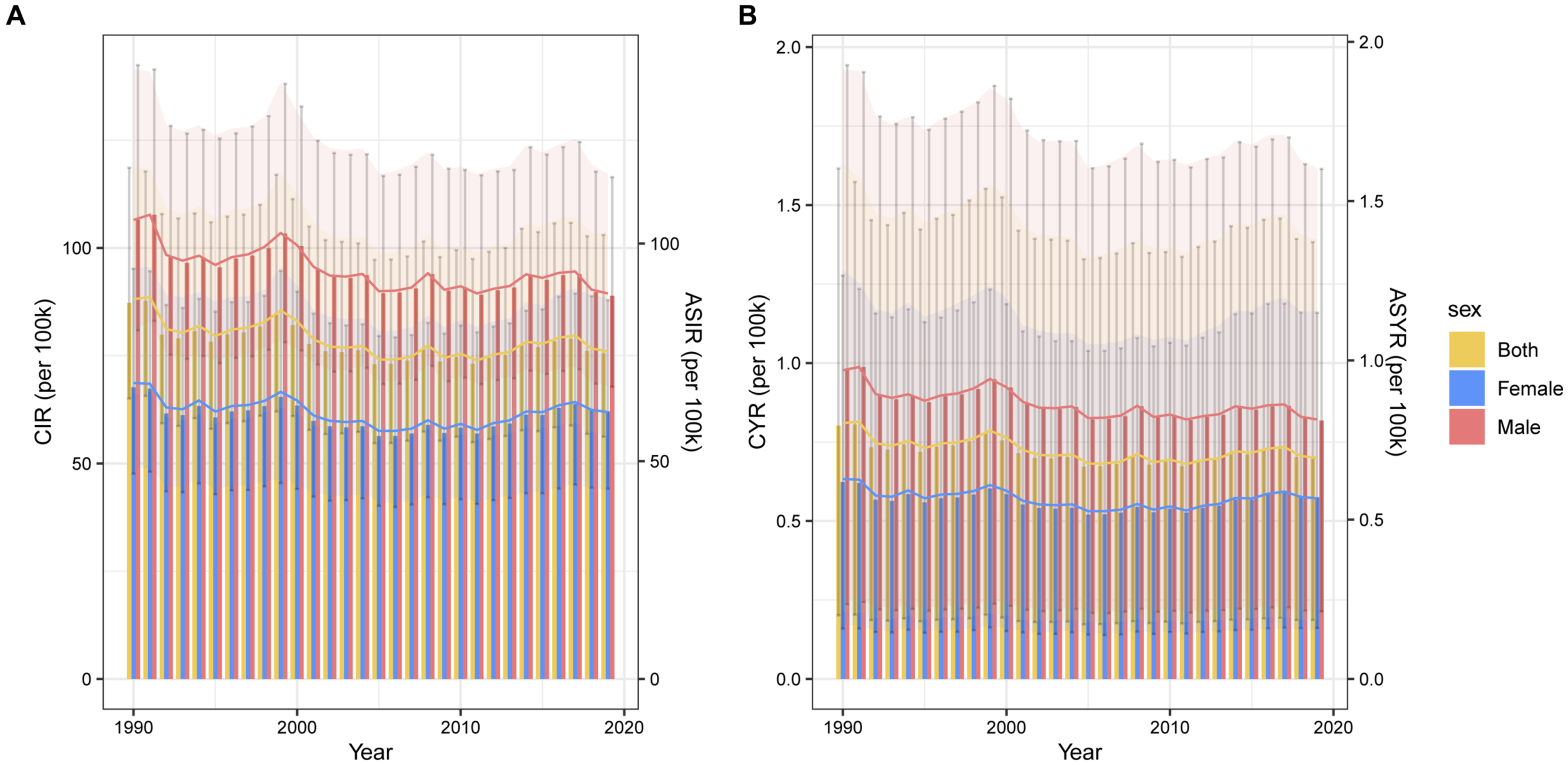
Global trends of incidence **(A)** and YLDs rate **(B)** of traumatic shoulder dislocation by gender from 1990 to 2019. The bar graphs represent the crude rate, corresponding to vertical ordinate at left. The line graphs represent the age-standardized rate, corresponding to vertical ordinate at right. CIR, crude incidence rate; ASIR, age-standardized incidence rate; YLDs, years lived with disability; CYR, crude YLDs rate; ASYR, age-standardized YLDs rate.

The burden of traumatic shoulder dislocation at the global level was reflected in Figure 1, both crude and age-standardized rates of incidence and YLDs in males were all higher than those in females. Over the course of 30 years, both crude and age-standardized rates of incidence and YLDs for all genders displayed a slight downward trend, accompanied by fluctuations.

In 2019, the global crude incidence rate of traumatic shoulder dislocation was 75.54 (95% UI 56.20–103.04) per 100,000 persons for both genders, 88.92 (95% UI 67.78–116.41) per 100,000 persons for males and 62.07 (95% UI 44.16–87.87) per 100,000 persons for females. The age-standardized incidence rate was 75.21 (95% UI 56.01–102.12) per 100,000 persons for both genders, 88.52 (95% UI 67.34–116.03) per 100,000 persons for males, and 61.29 (95% UI 43.34–87.53) per 100,000 persons for females in 2019.

Besides, in 2019, the crude YLDs rate was 0.70 (95% UI 0.19–1.38) per 100,000 persons for both genders, 0.82 (95% UI 0.21–1.61) per 100,000 persons for males, and 0.58 (95% UI 0.16–1.16) per 100,000 persons for females. The age-standardized YLDs rate was 0.69 (95% UI 0.18–1.38) per 100,000 persons for both genders, 0.81 (95% UI 0.21–1.60) per 100,000 persons for males, and 0.57 (95% UI 0.15–1.15) per 100,000 persons for females in 2019.

### The burden of traumatic shoulder dislocation at the regional level

As shown in Supplementary Table S1, in 2019, Australasia [195.78 (95% UI 138.38 to 285.05)], Central Europe [164.46 (95% UI 117.55 to 232.88)] and Eastern Europe [150.91 (95% UI 109.05 to 208.1)] had the highest age-standardized incidence rate per 100,000 persons of traumatic shoulder dislocation. For comparison, Central Sub-Saharan Africa [39.29 (95% UI 29.79 to 53.01)], Oceania [44.47 (95% UI 31.81 to 63.68)] and Southern Sub-Saharan Africa [46.3 (95% UI 34.1 to 61.46)] had the lowest age-standardized incidence rate. Further, most regions had a decreasing trend, except for 5 GBD regions (North Africa and Middle East, Caribbean, East Asia, Australasia, and Oceania). Specifically, North Africa and Middle East had the highest increasing trend [EAPC 1.78% (95% CI 1.16 to 2.41)], while Eastern Sub-Saharan Africa had the highest decreasing trend [EAPC −2.94% (95% CI −4.17 to −1.69)].

Supplementary Table S2 summarized YLDs rates of traumatic shoulder dislocation at the regional level. Australasia [1.79 (95% UI 0.43 to 3.74)], Central Europe [1.5 (95% UI 0.37 to 3.08)] and Eastern Europe [1.38 (95% UI 0.32 to 2.82)] had the highest age-standardized YLDs rate per 100,000 persons. In contrast, Central Sub-Saharan Africa [0.36 (95% UI 0.09 to 0.71)], Oceania [0.41 (95% UI 0.1 to 0.82)], and Southern Sub-Saharan Africa [0.43 (95% UI 0.12 to 0.85)] had the lowest age-standardized YLDs rate. Moreover, most regions displayed a decreasing trend, except for 5 GBD regions (North Africa and Middle East, Caribbean, East Asia, Australasia, and Oceania). In particular, Eastern Sub-Saharan Africa had the highest decreasing trend [EAPC −2.96% (−4.2 to −1.71)], while North Africa and Middle East had the highest increasing trend [EAPC 1.8% (1.17 to 2.44)].

### The burden of traumatic shoulder dislocation at the national level

Supplementary Table S3 showed that countries who had the highest age-standardized incidence rates per 100,000 persons in 2019 were Afghanistan [273.69 (95% UI 113.79 to 635.53)], Yemen [223.74 (104.73 to 482.89)] and New Zealand [222.54 (158.34 to 317.15)]. By contrast, Democratic People’s Republic of Korea [24.62 (95% UI 18.44 to 32.52)], Kiribati [29.47 (95% UI 21.28 to 40.56)] and Taiwan (Province of China) [30.16 (95% UI 22.15 to 41.47)] had the lowest age-standardized incidence rate. Furthermore, Liberia had the highest decreasing trend [EAPC −7.5% (95% CI −10.37 to −4.53)], while Syrian Arab Republic had the highest increasing trend [EAPC 10.69% (95% CI 7.07 to 14.44)].

As indicated in Supplementary Table S4, countries who had the highest age-standardized YLDs rates per 100,000 persons in 2019 were Afghanistan [2.55 (95% UI 0.55 to 7.01)], Yemen [2.07 (95% UI 0.45 to 5.69)] and New Zealand [2.03 (95% UI 0.48 to 4.12)]. In comparison, Democratic People’s Republic of Korea [0.23 (95% UI 0.06 to 0.46)], Kiribati [0.27 (95% UI 0.06 to 0.55)], and Taiwan (Province of China) [0.28 (95% UI 0.07 to 0.56)] had the lowest age-standardized YLDs rate. Furthermore, Lebanon had the highest decreasing trend [EAPC −7.5% (95% CI −10.37 to −4.53)], while Syrian Arab Republic had the highest increasing trend [EAPC 10.8% (95% CI 7.15 to 14.58)].

**Figure 2:**
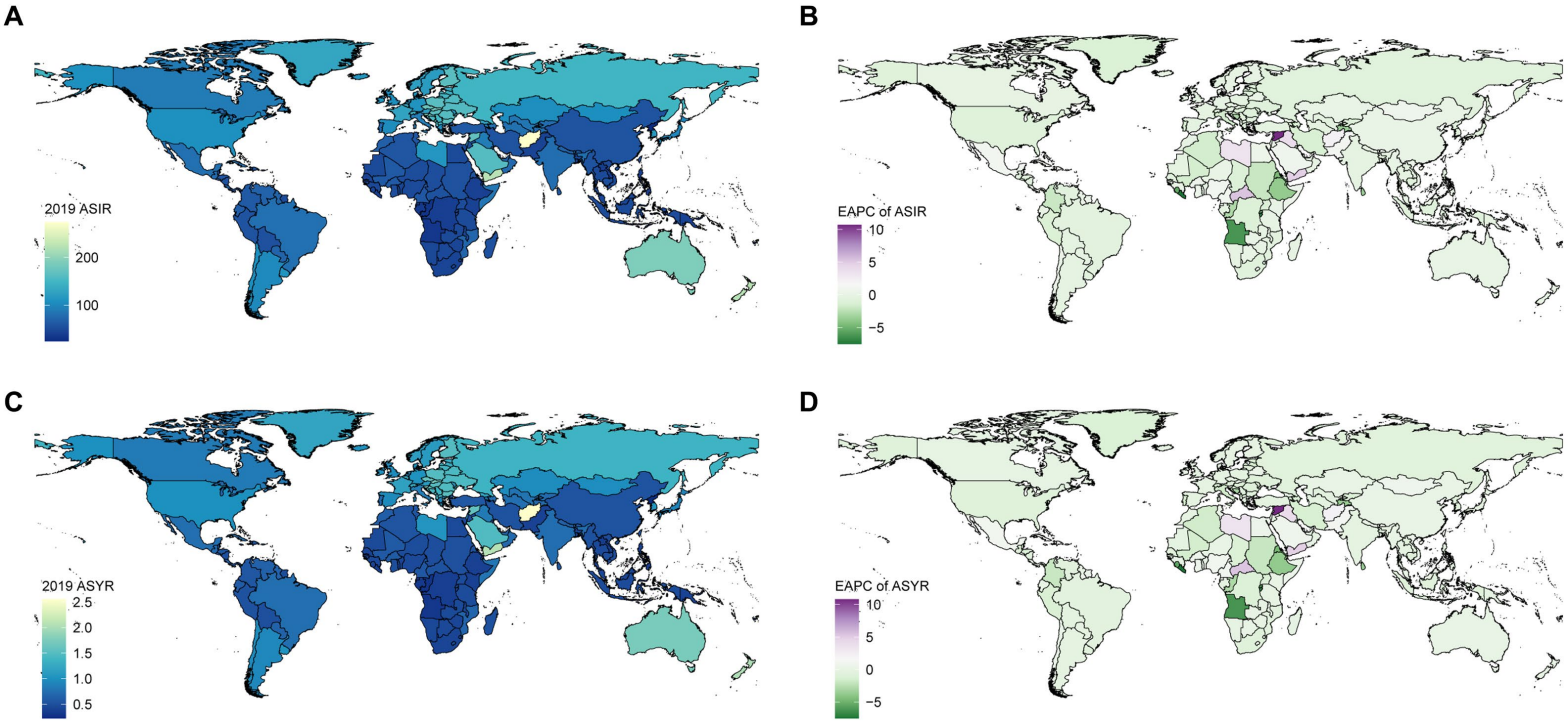
Global ASIR **(A)** and ASYR **(B)** of traumatic shoulder dislocation per 100 k population by country in 2019. EPAC of ASIR **(C)** and ASYR **(D)** of traumatic shoulder dislocation per 100 k population by country from 1990 to 2019. ASIR, age-standardized incidence rate; YLDs, years lived with disability; ASYR, age-standardized YLDs rate; EAPC, estimated annual percentage change.

Further visualization of the results was presented in the form of a map, where colors corresponded to the values of the parameter being studied (Figure 2).

### Age and gender patterns of traumatic shoulder dislocation

**Figure 3:**
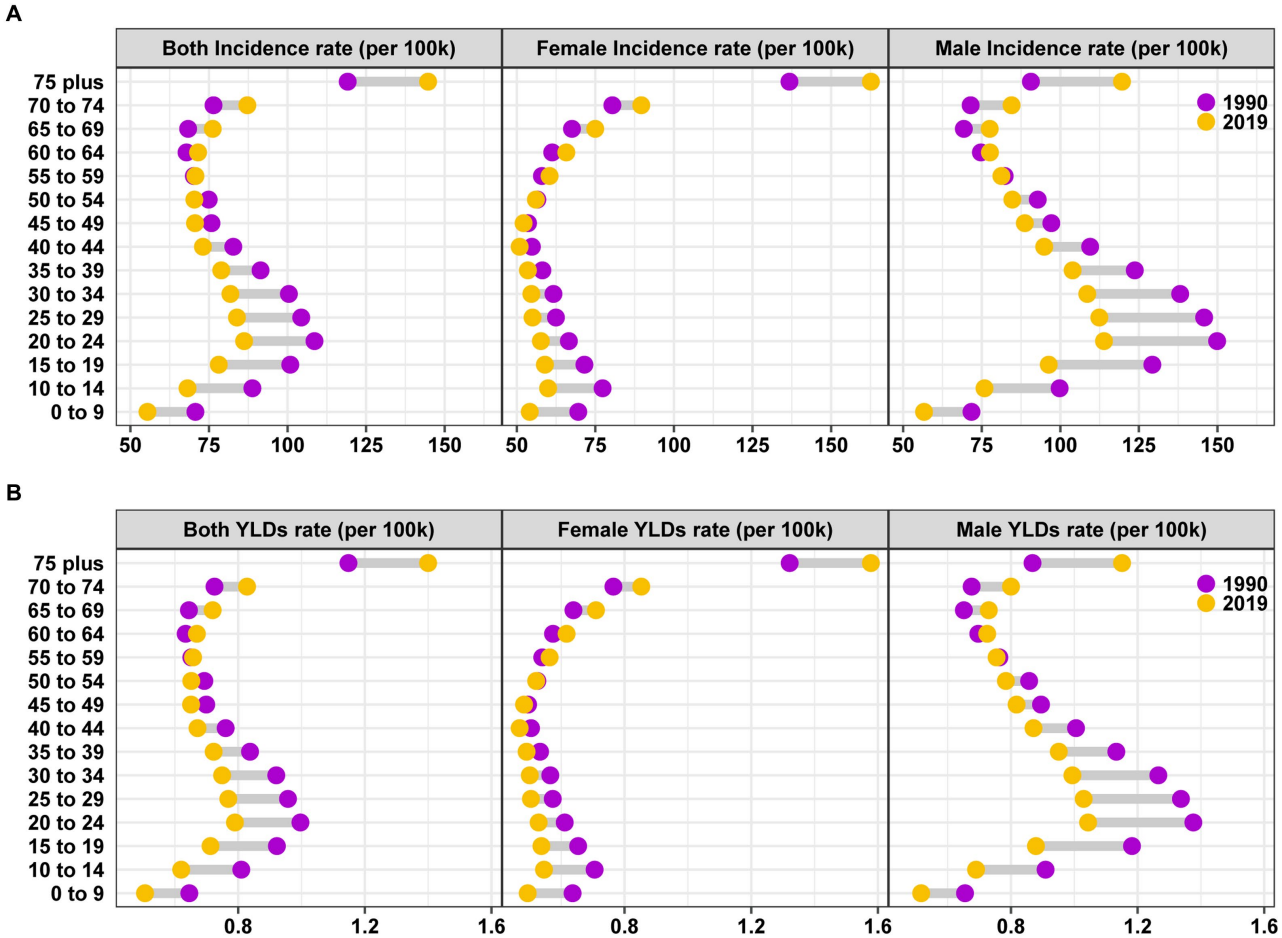
Global incidence **(A)** and YLDs **(B)** rate of traumatic shoulder dislocation by age and gender in 1990 and 2019. YLDs, years lived with disability.

The burden of traumatic shoulder dislocation in different genders and age groups between 1990 and 2019 was presented (Figure 3). The incidence rate among males peaked in the 20–24 age group, decreased thereafter, and then rose again around the age of 70. In contrast, the incidence rate among females reached its maximum in the 75 plus age group, significantly higher than other age groups (Figure 3A). The YLDs rate followed a similar trend as the incidence rate (Figure 3B). A comparison between 1990 and 2019 revealed a decrease in the overall burden of traumatic shoulder dislocation among younger individuals and an increase among older individuals.

### Leading causes of traumatic shoulder dislocation

**Figure 4:**
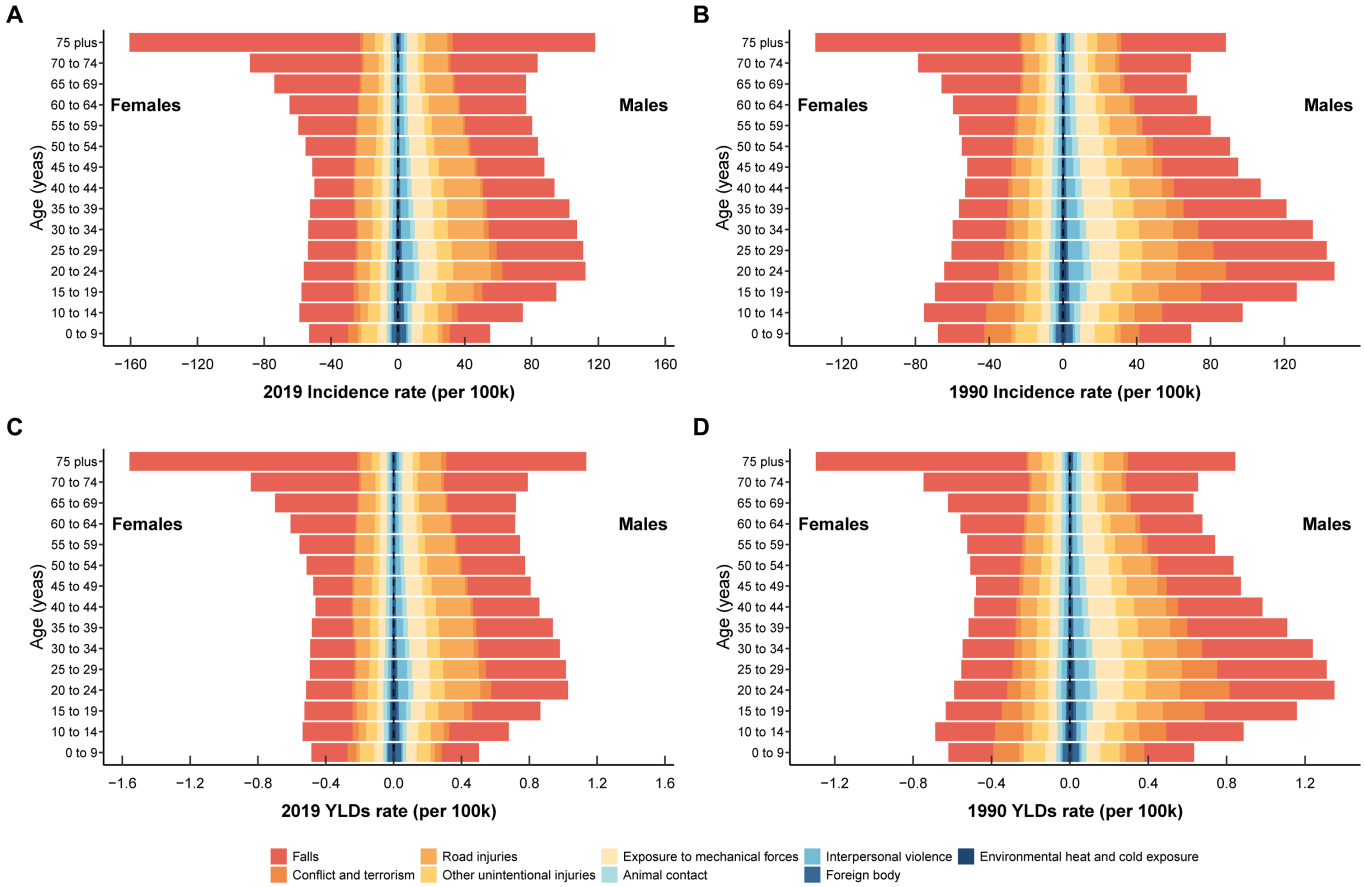
The incidence rate of the nine leading causes for traumatic shoulder dislocation in different age groups for males and females in 2019 **(A)** and1990 **(B)**. The YLDs rate of the nine leading causes for traumatic shoulder dislocation in different age groups for males and females in 2019 **(C)** and 1990 **(D)**. The nine leading causes are falls, conflict and terrorism, road injuries, other unintentional injuries, exposure to mechanical forces, animal contact, interpersonal violence, foreign body, environmental heat and cold exposure. YLDs, years lived with disability.

Figure 4 presented the incidence and YLDs rate of traumatic shoulder dislocations for nine main causes categorized by gender and age. For the entire population, the top three leading causes of incidence (Figure 4B) and YLDs (Figure 4D) rate in 1990 were falls, conflict and terrorism, and road injuries. In 2019, the top three leading causes of incidence rate (Figure 4A) and YLDs (Figure 4C) rate were falls, road injuries, and exposure to mechanical forces. In 1990, the incidence and YLDs rate caused by falls were 38.38 (95% UI 22.19–62.22) and 0.35 (95% UI 0.09–0.76), respectively. In 2019, the incidence rate and YLDs rate caused by falls were 38.82 (95% UI 22.83–62.59) and 0.36 (95% UI 0.10–0.76), respectively. Falls, as the primary cause of injury, are particularly noteworthy among the older adult population, especially in females.

### Correlation between age-standardized rate and SDI

**Figure 5:**
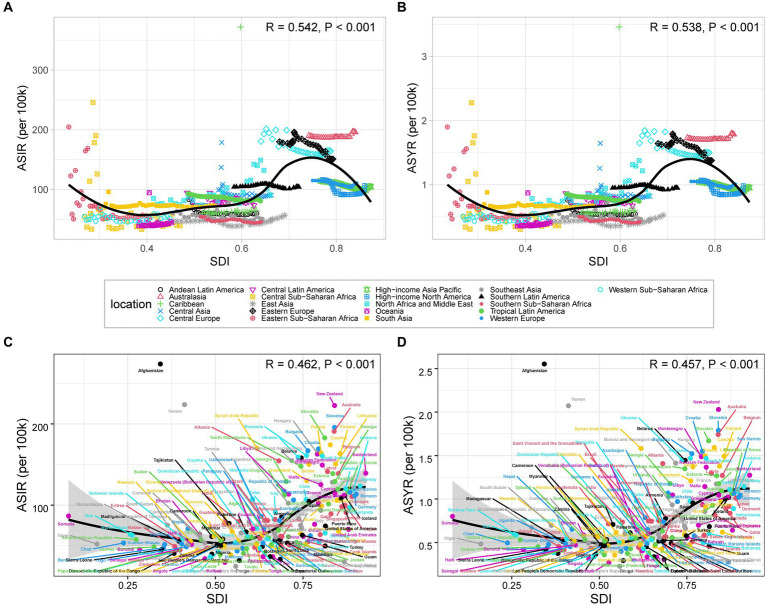
ASIR **(A)** and ASYR **(B)** of traumatic shoulder dislocation for 21 GBD regions by SDI from 1990 to 2019. ASIR **(C)** and ASYR **(D)** of traumatic shoulder dislocation for 204 countries and territories by SDI in 2019. The black line shows the trendline fit. The *R*- and *p*-values are calculated by Pearson’s correlation analysis. ASIR, age-standardized incidence rate; YLDs, years lived with disability; ASYR, age-standardized YLDs rate; SDI, sociodemographic index.

Overall, a positive correlation existed between age-standardized rates and SDI for traumatic shoulder dislocations (Figure 5). From 1990 to 2019, a sigmoidal trend was observed in age-standardized rates of incidence (Figure 5A) and YLDs (Figure 5B) for traumatic shoulder dislocations across 21 GBD regions as SDI increased. Western Europe, High-income Asia Pacific, and High-income North America exhibited lower disease burden, which accounted for the inflection point in the fitted curve. At the national level in 2019, the trend became more gradual (Figures 5C,D). Notably, several countries and regions, such as Afghanistan, Yemen, and New Zealand, have significantly higher age-standardized rate than expected.

## Discussion

Based on the global geographical regions and a three-decade time span, this study provides the latest, comprehensive, and systematic knowledge of the epidemiology and disease burden of traumatic shoulder dislocations. These findings can assist in data-driven healthcare policy-making and healthcare practices. The study presented the incidence and YLDs rates of traumatic shoulder dislocations at the global, regional, and national levels from 1990 to 2019, along with the temporal trends. Furthermore, the incidence and YLDs rates of traumatic shoulder dislocations were influenced by age, gender, and SDI. The incidence rate among males reached its peak in young adulthood, while females had the highest incidence rate in old age. The YLDs rate followed a similar pattern as the incidence rate. Overall, the burden of traumatic shoulder dislocation decreased among younger individuals and increased among older individuals between 1990 and 2019. Additionally, falls were the primary cause of the disease burden associated with traumatic shoulder dislocations. Lastly, there was a positive correlation between SDI and both age-standardized incidence and YLD rates.

Despite changes in production and lifestyle, as well as advancements in technology and medicine, the incidence and YLDs of traumatic shoulder dislocation did not significantly decrease. The disease burden among the older adult was rising while it was declining among young people. This implies that with the increasing aging population, traumatic shoulder dislocation is becoming a greater health burden. Additionally, the global distribution of traumatic shoulder dislocation is uneven, which may be related to different lifestyles, socioeconomic levels, climate, and geographic features. To note, Australasia, Central Europe, and Eastern Europe had a high disease burden and require considerable attention.

Exploring age and gender differences among traumatic shoulder dislocation patients can help determine which individuals will benefit from additional attention and care. This study found that the overall incidence rate in males was significantly higher than in females. Additionally, males and females demonstrated different epidemiological patterns. The incidence rate among males peaked in the age group of 20–24 and then decreased, with another increase around the age of 70. In females, the incidence rate was highest in the age group over 75, significantly higher than in other age groups. The trend in YLDs rate was similar to the incidence rate. Several epidemiological studies have shown a higher proportion of males compared to females in the distribution of shoulder dislocation (2, 4, 9–15, 27). The male–female pattern observed by Hindle et al. (28) was similar to ours. Patrick et al. (15) found the highest incidence rate in males aged 15–20. In contrast, females experienced relatively consistent rates of dislocation throughout their lives. After the age of 63, the incidence rate of dislocation in females was higher than in males (15). Studies conducted by Leroux et al. (13) and Shah et al. (27) were similar to this finding. Nabian et al. found that the incidence rate peaked in males at ages 21–30 and then rapidly declined, while it consistently remained low in females (2). Liavaag et al. (14) discovered a peak incidence rate among males aged 20–29, as well as an upward trend in incidence rates for both males and females in older age groups.

Men, especially young men, had a higher disease burden, which may be due to their greater participation in high-intensity and high-risk physical activities. The incidence of shoulder dislocation in the U.S. military population was much higher compared to the general population, with a total incidence rate of 169/100000 (29). The strength of Owens et al. study lies in its large cohort consisting of physically active individuals and the use of standardized databases, which may represent the population regularly engaged in sports and exercise. Older women were another major risk group for shoulder dislocation. Age-related muscle loss increases reaction time, leading to functional impairments and an increased risk of falls (30, 31). Importantly, the age-related changes may vary between genders (32, 33). The above survey results indicate the need for greater attention to these specific populations. In addition, interventions and preventive strategies targeting high-risk groups are advisable.

Zacchilli et al. (9) reported that falls were the most common cause of shoulder dislocation, accounting for 58.8% of cases. In Nordqvist et al. (12) study, falls were the most common cause of shoulder dislocation, with indoor falls accounting for 62.5% of cases in the population aged 65 and above. Yeap et al. (34) found that the most common mechanisms of injury were falls and direct impact. Direct collisions or falls onto the shoulder accounted for the most common cause of initial dislocations (55%). Our study found similar results, with falls being the primary cause of traumatic shoulder dislocation. It is particularly important to note that falls leading to traumatic shoulder dislocation were particularly severe in the older adult, especially in women. It can be expected that with the arrival of an aging population, the incidence of traumatic shoulder dislocation among the older adult will continue to increase, posing higher demands on future healthcare services. Falls in the older adult are a significant public health issue (35, 36). Enhancing preventive measures to ensure safety at home for the older adult should be given great attention. Hospitals, communities, and care institutions need to work together to implement a comprehensive and systematic strategy to prevent falls among the older adult and the potential consequent injuries (37–39).

Several studies have shown a clear association between SDI and orthopedic diseases, such as osteoarthritis (21), rheumatoid arthritis (22), fractures (23, 24), and osteoporosis (25). Similarly, we found some associations between traumatic shoulder dislocation and SDI. Possible explanations for this phenomenon may be complex. Theoretically, people in high SDI countries often have access to high-quality healthcare services and longer life expectancies. Therefore, this disease imposes a heavier burden on society. Additionally, universal health coverage in high SDI countries may contribute to proactive detection of shoulder dislocation cases. The association with SDI provides evidence for establishing precise strategies for prevention and management of shoulder dislocation at the national level. Economic conditions have been found to be related to the incidence rate of joint dislocations (28). The incidence rate was high among both the wealthiest and poorest populations (28). Wealthy individuals are more capable of affording relatively higher medical expenses, allowing them to seek timely and early medical consultations and treatments. Individuals in an economically disadvantaged position are more likely to engage in hazardous work and face an increased risk of traumatic shoulder dislocations.

### Limitations of this study

There are several limitations to our research findings that need to be mentioned. Firstly, the accuracy of the results is related to the quality and quantity of representative studies included in GBD 2019. For example, lower-SDI countries tend to lack high-quality research. Besides, different data sources and statistical methods across different countries and regions may introduce biases. Secondly, GBD 2019 only provides data at the national level, making it impossible to obtain data for different regions within a country. Therefore, caution should be exercised when extrapolating the results to subnational levels. Thirdly, no distinction is made between different types of traumatic shoulder dislocations. As a result, we are unable to study subtypes of traumatic shoulder dislocation in depth. Fourthly, patients with shoulder dislocation may experience spontaneous reduction, which may lead to underestimation of the true incidence. Finally, estimations of multiple injuries or combined injuries may be subject to bias. GBD 2019 has developed an injuries severity hierarchy, which selects the injury nature category that may cause the greatest burden in cases of multiple injuries.

## Conclusion

The disease burden of traumatic shoulder dislocation has not significantly decreased from 1990 to 2019. The incidence and YLD rates are associated with age, gender, and SDI. A thorough examination of the disease burden contributes to the efficient allocation and utilization of resources, as well as the development of targeted and effective intervention strategies.

## Data availability statement

Publicly available datasets were analyzed in this study. This data can be found at: the datasets analyzed in the current study can be acquired from the official website of the GBD study, and request to the original data for this study can also be made upon reasonable aims via the corresponding author’s E-mail.

## Ethics statement

The studies involving humans and GBD study’s protocol were approved by the Research Ethics Board at the University of Washington. This study was a secondary analysis of GBD 2019, using public data. Thus, ethical approval and consent from an institutional review board or ethics committee was not required for secondary analysis of data in this study. The studies were conducted in accordance with the local legislation and institutional requirements. Written informed consent for participation was not required from the participants or the participants’ legal guardians/next of kin in accordance with the national legislation and institutional requirements.

## Author contributions

CC: Conceptualization, Writing – original draft. TY: Conceptualization, Data curation, Formal analysis, Methodology, Visualization, Writing – original draft. JJ: Investigation, Writing – review & editing. WH: Investigation, Writing – review & editing. JX: Writing – original draft. YY: Project administration, Supervision, Writing – review & editing.

## Footnotes

^1^https://ghdx.healthdata.org/gbd-results

^2^https://ghdx.healthdata.org/gbd-2019

^3^https://ghdx.healthdata.org/countries

^4^https://ghdx.healthdata.org/record/ihme-data/global-population-forecasts-2017-2100

## References

[1] Yang NP Chen HC Phan DV Yu IL Lee YH Chan CL. Epidemiological survey of orthopedic joint dislocations based on nationwide insurance data in Taiwan, 2000–2005. BMC Musculoskelet Disord. (2011) 12:253. doi: 10.1186/1471-2474-12-253, PMID: 22053727 PMC3228707

[2] Nabian MH Zadegan SA Zanjani LO Mehrpour SR. Epidemiology of joint dislocations and ligamentous/tendinous injuries among 2,700 patients: five-year trend of a tertiary center in Iran. Arch Bone Jt Surg. (2017) 5:426–34. PMID: 29299498 PMC5736892

[3] Zhao WG Ma JT Yan XL Zhu YB Zhang YZ. Epidemiological characteristics of major joints fracture-dislocations. Orthop Surg. (2021) 13:2310–7. doi: 10.1111/os.13162, PMID: 34708546 PMC8654670

[4] Hovelius L. Incidence of shoulder dislocation in Sweden. Clin Orthop Relat Res. (1982) 166:127–31. doi: 10.1097/00003086-198206000-00021 7083659

[5] Van Tongel A Rosa F Heffernan G Levy O Sforza G. Long-term result after traumatic anterior shoulder dislocation: what works best? Musculoskelet Surg. (2011) 95:65–70. doi: 10.1007/s12306-011-0125-8, PMID: 21503722

[6] Sachs RA Lin D Stone ML Paxton E Kuney M. Can the need for future surgery for acute traumatic anterior shoulder dislocation be predicted? J Bone Joint Surg Am. (2007) 89:1665–74. doi: 10.2106/JBJS.F.00261, PMID: 17671003

[7] Kavaja L Lahdeoja T Malmivaara A Paavola M. Treatment after traumatic shoulder dislocation: a systematic review with a network meta-analysis. Br J Sports Med. (2018) 52:1498–506. doi: 10.1136/bjsports-2017-098539, PMID: 29936432 PMC6241619

[8] Hovelius LK Sandstrom BC Rosmark DL Saebo M Sundgren KH Malmqvist BG. Long-term results with the Bankart and Bristow-Latarjet procedures: recurrent shoulder instability and arthropathy. J Shoulder Elb Surg. (2001) 10:445–52. doi: 10.1067/mse.2001.117128, PMID: 11641702

[9] Zacchilli MA Owens BD. Epidemiology of shoulder dislocations presenting to emergency departments in the United States. J Bone Joint Surg Am. (2010) 92:542–9. doi: 10.2106/JBJS.I.00450, PMID: 20194311

[10] Simonet WT Melton LJ 3rd Cofield RH Ilstrup DM. Incidence of anterior shoulder dislocation in Olmsted County, Minnesota. Clin Orthop Relat Res. (1984) 186:186–91. doi: 10.1097/00003086-198406000-00030 6723141

[11] Kroner K Lind T Jensen J. The epidemiology of shoulder dislocations. Arch Orthop Trauma Surg. (1989) 108:288–90. doi: 10.1007/BF00932317 2789505

[12] Nordqvist A Petersson CJ. Incidence and causes of shoulder girdle injuries in an urban population. J Shoulder Elb Surg. (1995) 4:107–12. doi: 10.1016/s1058-2746(05)80063-1, PMID: 7600160

[13] Leroux T Wasserstein D Veillette C Khoshbin A Henry P Chahal J. Epidemiology of primary anterior shoulder dislocation requiring closed reduction in Ontario, Canada. Am J Sports Med. (2014) 42:442–50. doi: 10.1177/0363546513510391, PMID: 24275862

[14] Liavaag S Svenningsen S Reikeras O Enger M Fjalestad T Pripp AH. The epidemiology of shoulder dislocations in Oslo. Scand J Med Sci Sports. (2011) 21:e334–40. doi: 10.1111/j.1600-0838.2011.01300.x, PMID: 21507063 PMC3274702

[15] Patrick CM Snowden J Eckhoff MD Green CK Scanaliato JP Dunn JC. Epidemiology of shoulder dislocations presenting to United States emergency departments: an updated ten-year study. World J Orthop. (2023) 14:690–7. doi: 10.5312/wjo.v14.i9.690, PMID: 37744717 PMC10514709

[16] GBD 2019 Diseases and Injuries Collaborators. Global burden of 369 diseases and injuries in 204 countries and territories, 1990–2019: a systematic analysis for the Global Burden of Disease study 2019. Lancet. (2020) 396:1204–22. doi: 10.1016/s0140-6736(20)30925-9, PMID: 33069326 PMC7567026

[17] Cieza A Causey K Kamenov K Hanson SW Chatterji S Vos T. Global estimates of the need for rehabilitation based on the Global Burden of Disease study 2019: a systematic analysis for the Global Burden of Disease study 2019. Lancet. (2021) 396:2006–17. doi: 10.1016/s0140-6736(20)32340-0, PMID: 33275908 PMC7811204

[18] GBD 2019 Fracture Collaborators. Global, regional, and national burden of bone fractures in 204 countries and territories, 1990–2019: a systematic analysis from the Global Burden of Disease study 2019. Lancet Healthy Longev. (2021) 2:e580–92. doi: 10.1016/s2666-7568(21)00172-0, PMID: 34723233 PMC8547262

[19] Singh A Das S Chopra A Danda D Paul BJ March L. Burden of osteoarthritis in India and its states, 1990–2019: findings from the Global Burden of Disease study 2019. Osteoarthr Cartil. (2022) 30:1070–8. doi: 10.1016/j.joca.2022.05.004, PMID: 35598766

[20] Hassen N Lacaille D Xu A Alandejani A Sidi S Mansourian M. National burden of rheumatoid arthritis in Canada, 1990–2019: findings from the Global Burden of Disease study 2019—a GBD collaborator-led study. RMD Open. (2024) 10:e003533. doi: 10.1136/rmdopen-2023-003533, PMID: 38216285 PMC10806499

[21] Ding Y Liu X Chen C Yin C Sun X. Global, regional, and national trends in osteoarthritis disability-adjusted life years (DALYs) from 1990 to 2019: a comprehensive analysis of the Global Burden of Disease study. Public Health. (2024) 226:261–72. doi: 10.1016/j.puhe.2023.10.030, PMID: 38134839

[22] Safiri S Kolahi AA Hoy D Smith E Bettampadi D Mansournia MA. Global, regional and national burden of rheumatoid arthritis 1990–2017: a systematic analysis of the Global Burden of Disease study 2017. Ann Rheum Dis. (2019) 78:1463–71. doi: 10.1136/annrheumdis-2019-215920 31511227

[23] Hu S Guo J Zhu B Dong Y Li F. Epidemiology and burden of pelvic fractures: results from the Global Burden of Disease study 2019. Injury. (2023) 54:589–97. doi: 10.1016/j.injury.2022.12.007, PMID: 36528424

[24] Dong Y Peng R Kang H Song K Guo Q Zhao H. Global incidence, prevalence, and disability of vertebral fractures: a systematic analysis of the Global Burden of Disease study 2019. Spine J. (2022) 22:857–68. doi: 10.1016/j.spinee.2021.12.007, PMID: 34906740

[25] Shen Y Huang X Wu J Lin X Zhou X Zhu Z. The global burden of osteoporosis, low bone mass, and its related fracture in 204 countries and territories, 1990–2019. Front Endocrinol. (2022) 13:882241. doi: 10.3389/fendo.2022.882241, PMID: 35669691 PMC9165055

[26] Hankey BF Ries LA Kosary CL Feuer EJ Merrill RM Clegg LX. Partitioning linear trends in age-adjusted rates. Cancer Causes Control. (2000) 11:31–5. doi: 10.1023/a:1008953201688, PMID: 10680727

[27] Shah A Judge A Delmestri A Edwards K Arden NK Prieto-Alhambra D. Incidence of shoulder dislocations in the UK, 1995–2015: a population-based cohort study. BMJ Open. (2017) 7:e016112. doi: 10.1136/bmjopen-2017-016112, PMID: 29138197 PMC5695490

[28] Hindle P Davidson EK Biant LC Court-Brown CM. Appendicular joint dislocations. Injury. (2013) 44:1022–7. doi: 10.1016/j.injury.2013.01.043, PMID: 23433660

[29] Owens BD Dawson L Burks R Cameron KL. Incidence of shoulder dislocation in the United States military: demographic considerations from a high-risk population. J Bone Joint Surg Am. (2009) 91:791–6. doi: 10.2106/JBJS.H.00514, PMID: 19339562

[30] Kim TN Choi KM. Sarcopenia: definition, epidemiology, and pathophysiology. J Bone Metab. (2013) 20:1–10. doi: 10.11005/jbm.2013.20.1.1, PMID: 24524049 PMC3780834

[31] Pereira da Silva AlvesIISantos BuenoGABrito ElmescanyRAparecida BorgesLKran PintoDCorreia MartinsA. Motor reaction time, sarcopenia and functional skills in elderly women: a cross-sectional study. J Nutr Health Aging. (2023) 27:878–84. doi: 10.1007/s12603-023-1983-0, PMID: 37960911

[32] Gheller BJ Riddle ES Lem MR Thalacker-Mercer AE. Understanding age-related changes in skeletal muscle metabolism: differences between females and males. Annu Rev Nutr. (2016) 36:129–56. doi: 10.1146/annurev-nutr-071715-050901, PMID: 27431365

[33] Dong Y Kang H Peng R Song K Guo Q Guan H. Global, regional, and national burden of low bone mineral density from 1990 to 2019: results from the Global Burden of Disease study 2019. Front Endocrinol. (2022) 13:870905. doi: 10.3389/fendo.2022.870905, PMID: 35685212 PMC9172621

[34] Yeap JS Lee DJ Fazir M Borhan TA Kareem BA. The epidemiology of shoulder dislocations in Malaysia. Med J Malaysia. (2004) 59:19–23. PMID: 15941156

[35] James SL Lucchesi LR Bisignano C Castle CD Dingels ZV Fox JT. The global burden of falls: global, regional and national estimates of morbidity and mortality from the Global Burden of Disease study 2017. Inj Prev. (2020) 26:i3–i11. doi: 10.1136/injuryprev-2019-043286, PMID: 31941758 PMC7571347

[36] Haagsma JA Olij BF Majdan M Van Beeck EF Vos T Castle CD. Falls in older aged adults in 22 European countries: incidence, mortality and burden of disease from 1990 to 2017. Inj Prev. (2020) 26:i67–74. doi: 10.1136/injuryprev-2019-043347, PMID: 32111726 PMC7571349

[37] Clemson L Stark S Pighills AC Fairhall NJ Lamb SE Ali J. Environmental interventions for preventing falls in older people living in the community. Cochrane Database Syst Rev. (2023) 2023:Cd013258. doi: 10.1002/14651858.CD013258.pub2, PMID: 36893804 PMC9998238

[38] Kannus P Sievänen H Palvanen M Järvinen T Parkkari J. Prevention of falls and consequent injuries in elderly people. Lancet. (2005) 366:1885–93. doi: 10.1016/s0140-6736(05)67604-0 16310556

[39] Close JCT Lord SR. Fall prevention in older people: past, present and future. Age Ageing. (2022) 51:afac105. doi: 10.1093/ageing/afac105, PMID: 35754196

