# Supplementary material for: Exploring temporal trends and burden of traumatic shoulder dislocation: a global perspective
Source: Front Public Health. 2024 Feb 29;12:1346957. doi: 10.3389/fpubh.2024.1346957 (PMC10937414; doi:10.3389/fpubh.2024.1346957)
Supplement: Supplementary file 1 [file Table_1.DOCX]

**Global Epidemiology and Trends of Traumatic Shoulder Dislocation**

**Supplementary Material**

**Contents**

**Table S1………………………………………………………………………………………. 2**

**Table S2……………………………………………………………………………….……… 7**

**Table S3………………………………………………………………………………………. 12**

**Table S4………………………………………………………………………………………. 53**

Table S1. Incidence of traumatic shoulder dislocation in 1990 and 2019 for both sexes in globe and 21 GBD regions, with EAPC from 1990 and 2019

| **Location** | **Number** | | | **CR, per 100k** | | | **ASR, per 100k** | | |
| --- | --- | --- | --- | --- | --- | --- | --- | --- | --- |
|  | **Number in 1990 (95% UI)** | **Number in 2019 (95% UI)** | **Number change (95% UI)** | **CR in 1990 (95% UI)** | **CR in 2019 (95% UI)** | **EAPC of CR (95% CI)** | **ASR in 1990 (95% UI)** | **ASR in 2019 (95% UI)** | **EAPC of ASR (95% CI)** |
| Globe | 4667551 (3481087 to 6344843) | 5844810 (4348552 to 7972849) | 0.25% (0.11 to 0.35) | 87.25 (65.07 to 118.6) | 75.54 (56.2 to 103.04) | -0.36% (-0.52 to -0.2) | 87.21 (65.09 to 117.76) | 75.21 (56.01 to 102.12) | -0.38% (-0.53 to -0.23) |
| Andean Latin America | 34234 (24180 to 52643) | 38607 (28719 to 52290) | 0.13% (-0.26 to 0.5) | 89.67 (63.34 to 137.89) | 60.71 (45.16 to 82.22) | -0.9% (-1.25 to -0.56) | 83.12 (59.12 to 124.58) | 59.88 (44.6 to 81.05) | -0.72% (-1.05 to -0.39) |
| Australasia | 37526 (26723 to 53155) | 53257 (37869 to 76172) | 0.42% (0.34 to 0.5) | 185.06 (131.79 to 262.14) | 183.24 (130.29 to 262.09) | -0.03% (-0.1 to 0.04) | 190.2 (135.84 to 269.99) | 195.78 (138.38 to 285.05) | 0.09% (0.04 to 0.15) |
| Caribbean | 21657 (16672 to 28199) | 30744 (23234 to 41186) | 0.42% (0.33 to 0.51) | 61.4 (47.27 to 79.94) | 65.18 (49.26 to 87.32) | 0.61% (-0.75 to 2) | 59.91 (46.06 to 78.25) | 65.27 (49.38 to 87.56) | 0.71% (-0.66 to 2.09) |
| Central Asia | 72309 (53384 to 99240) | 86371 (63445 to 118613) | 0.19% (0.16 to 0.23) | 104.39 (77.07 to 143.27) | 92.35 (67.83 to 126.82) | -1.08% (-1.58 to -0.58) | 99.26 (72.96 to 135.38) | 90.32 (66.39 to 123.95) | -0.96% (-1.46 to -0.46) |
| Central Europe | 219629 (159954 to 305028) | 174277 (124085 to 247034) | -0.21% (-0.26 to -0.16) | 178.61 (130.08 to 248.06) | 152.57 (108.63 to 216.27) | -0.92% (-1.09 to -0.74) | 180.52 (131.39 to 251.07) | 164.46 (117.55 to 232.88) | -0.71% (-0.91 to -0.5) |
| Central Latin America | 162327 (119491 to 223439) | 183448 (133054 to 252327) | 0.13% (-0.02 to 0.22) | 98.91 (72.81 to 136.14) | 73.37 (53.22 to 100.92) | -0.15% (-0.46 to 0.16) | 94.42 (69.48 to 128.66) | 73.61 (53.58 to 101.79) | -0.02% (-0.31 to 0.27) |
| Central Sub-Saharan Africa | 32390 (21122 to 54838) | 53337 (40052 to 73180) | 0.65% (0.23 to 1.08) | 58.34 (38.04 to 98.77) | 40.55 (30.45 to 55.63) | -2.78% (-4.9 to -0.62) | 54.32 (36.91 to 89.42) | 39.29 (29.79 to 53.01) | -2.6% (-4.6 to -0.55) |
| East Asia | 569536 (397230 to 809864) | 915345 (644521 to 1248940) | 0.61% (0.44 to 0.8) | 46.49 (32.42 to 66.1) | 62.18 (43.78 to 84.83) | 0.5% (0.09 to 0.9) | 46.79 (33.04 to 66) | 56.95 (40.31 to 77.85) | 0.15% (-0.24 to 0.54) |
| Eastern Europe | 403193 (293559 to 546033) | 303890 (219720 to 421529) | -0.25% (-0.28 to -0.21) | 178.01 (129.6 to 241.07) | 144.73 (104.64 to 200.76) | -0.69% (-0.97 to -0.4) | 179.98 (130.81 to 242.94) | 150.91 (109.05 to 208.1) | -0.64% (-0.85 to -0.43) |
| Eastern Sub-Saharan Africa | 423196 (189151 to 944512) | 211184 (158186 to 282187) | -0.5% (-0.76 to 0.07) | 222.54 (99.47 to 496.68) | 51.29 (38.42 to 68.53) | -3.12% (-4.46 to -1.77) | 204.69 (96.43 to 436.07) | 51.94 (39.26 to 69.1) | -2.94% (-4.17 to -1.69) |
| High-income Asia Pacific | 197886 (144723 to 274793) | 187689 (136060 to 258016) | -0.05% (-0.1 to 0) | 114.05 (83.41 to 158.37) | 100.21 (72.65 to 137.76) | -0.61% (-0.7 to -0.52) | 114.19 (83.36 to 160.25) | 104.79 (75.07 to 148.73) | -0.46% (-0.57 to -0.35) |
| High-income North America | 332837 (239801 to 464493) | 425946 (309970 to 586481) | 0.28% (0.19 to 0.38) | 118.48 (85.36 to 165.34) | 116.84 (85.03 to 160.87) | -0.27% (-0.51 to -0.02) | 116.38 (84.14 to 164.26) | 106.34 (77.7 to 148) | -0.69% (-0.97 to -0.41) |
| North Africa and Middle East | 309658 (228642 to 425585) | 564323 (394755 to 851264) | 0.82% (0.58 to 1.1) | 89.75 (66.27 to 123.35) | 92.71 (64.85 to 139.85) | 1.64% (1.01 to 2.27) | 85.16 (63.71 to 116.27) | 91.32 (63.36 to 139.39) | 1.78% (1.16 to 2.41) |
| Oceania | 2549 (1905 to 3477) | 5852 (4162 to 8285) | 1.3% (1.02 to 1.52) | 39.4 (29.44 to 53.74) | 44.08 (31.35 to 62.41) | -0.04% (-0.71 to 0.63) | 39.27 (29.32 to 53.11) | 44.47 (31.81 to 63.68) | 0.01% (-0.66 to 0.68) |
| South Asia | 803573 (597993 to 1078140) | 1295971 (946475 to 1770065) | 0.61% (0.45 to 0.75) | 73.21 (54.48 to 98.22) | 71.79 (52.43 to 98.05) | -0.15% (-0.4 to 0.1) | 75.86 (56.87 to 101.87) | 72.97 (53.33 to 99.89) | -0.22% (-0.44 to 0) |
| Southeast Asia | 285648 (213790 to 392488) | 340880 (253698 to 456759) | 0.19% (0 to 0.35) | 61.19 (45.8 to 84.08) | 50.59 (37.65 to 67.79) | -0.56% (-0.95 to -0.18) | 60.1 (45.31 to 81.77) | 50.7 (37.87 to 67.87) | -0.51% (-0.89 to -0.12) |
| Southern Latin America | 52146 (37888 to 72431) | 67451 (48949 to 95047) | 0.29% (0.24 to 0.34) | 105.25 (76.48 to 146.2) | 101.05 (73.33 to 142.39) | -0.25% (-0.35 to -0.15) | 104.29 (75.78 to 144.96) | 104.14 (75.67 to 146.22) | -0.1% (-0.19 to -0.01) |
| Southern Sub-Saharan Africa | 28383 (20665 to 37741) | 36453 (26710 to 48597) | 0.28% (0.17 to 0.38) | 54.07 (39.37 to 71.9) | 46.39 (33.99 to 61.85) | -0.43% (-0.59 to -0.27) | 54.83 (39.64 to 73.03) | 46.3 (34.1 to 61.46) | -0.51% (-0.66 to -0.35) |
| Tropical Latin America | 144965 (102638 to 204853) | 182099 (129296 to 254601) | 0.26% (0.19 to 0.32) | 94.82 (67.14 to 134) | 81.44 (57.83 to 113.87) | -0.49% (-0.54 to -0.43) | 93.17 (66.38 to 130.53) | 80.76 (57.14 to 113.08) | -0.42% (-0.48 to -0.37) |
| Western Europe | 432833 (311081 to 615069) | 467987 (329876 to 674879) | 0.08% (0.02 to 0.14) | 112.54 (80.89 to 159.93) | 107.26 (75.61 to 154.68) | -0.34% (-0.42 to -0.26) | 115.04 (82.53 to 162.66) | 110.73 (76.21 to 161.49) | -0.29% (-0.36 to -0.22) |
| Western Sub-Saharan Africa | 101076 (71821 to 146277) | 219697 (159558 to 298918) | 1.17% (0.91 to 1.37) | 52.48 (37.29 to 75.96) | 48.15 (34.97 to 65.51) | -0.19% (-0.66 to 0.29) | 54.94 (39.32 to 77.23) | 51.87 (37.52 to 70.06) | -0.08% (-0.48 to 0.33) |

CR, crude rate; ASR, age-standardized rate; EAPC, estimated annual percentage change; UI, uncertainty interval; CI, confidence interval.

Table S2. YLDs of traumatic shoulder dislocation in 1990 and 2019 for both sexes in globe and 21 GBD regions, with EAPC from 1990 and 2019.

| **Location** | **Number** | | | **CR, per 100k** | | | **ASR, per 100k** | | |
| --- | --- | --- | --- | --- | --- | --- | --- | --- | --- |
|  | **Number in 1990 (95% UI)** | **Number in 2019 (95% UI)** | **Number change (95% UI)** | **CR in 1990 (95% UI)** | **CR in 2019 (95% UI)** | **EAPC of CR (95% CI)** | **ASR in 1990 (95% UI)** | **ASR in 2019 (95% UI)** | **EAPC of ASR (95% CI)** |
| Globe | 42902 (10786 to 86432) | 53927 (14437 to 106991) | 0.26% (0.11 to 0.41) | 0.8 (0.2 to 1.62) | 0.7 (0.19 to 1.38) | -0.34% (-0.5 to -0.18) | 0.8 (0.21 to 1.62) | 0.69 (0.18 to 1.38) | -0.37% (-0.52 to -0.22) |
| Andean Latin America | 314 (70 to 672) | 353 (82 to 701) | 0.12% (-0.26 to 0.49) | 0.82 (0.18 to 1.76) | 0.55 (0.13 to 1.1) | -0.91% (-1.27 to -0.56) | 0.76 (0.18 to 1.61) | 0.55 (0.13 to 1.08) | -0.74% (-1.07 to -0.4) |
| Australasia | 342 (82 to 703) | 488 (120 to 999) | 0.43% (0.34 to 0.61) | 1.69 (0.41 to 3.47) | 1.68 (0.41 to 3.44) | -0.01% (-0.08 to 0.06) | 1.73 (0.41 to 3.57) | 1.79 (0.43 to 3.74) | 0.1% (0.04 to 0.15) |
| Caribbean | 198 (48 to 391) | 282 (71 to 556) | 0.42% (0.33 to 0.57) | 0.56 (0.14 to 1.11) | 0.6 (0.15 to 1.18) | 0.63% (-0.75 to 2.02) | 0.55 (0.14 to 1.08) | 0.6 (0.15 to 1.18) | 0.71% (-0.67 to 2.1) |
| Central Asia | 659 (144 to 1324) | 788 (179 to 1593) | 0.2% (0.16 to 0.24) | 0.95 (0.21 to 1.91) | 0.84 (0.19 to 1.7) | -1.09% (-1.59 to -0.58) | 0.91 (0.2 to 1.81) | 0.82 (0.19 to 1.67) | -0.97% (-1.47 to -0.46) |
| Central Europe | 2010 (483 to 4011) | 1599 (392 to 3240) | -0.2% (-0.25 to -0.14) | 1.63 (0.39 to 3.26) | 1.4 (0.34 to 2.84) | -0.91% (-1.09 to -0.74) | 1.65 (0.39 to 3.31) | 1.5 (0.37 to 3.08) | -0.72% (-0.92 to -0.51) |
| Central Latin America | 1487 (359 to 2987) | 1683 (424 to 3399) | 0.13% (-0.03 to 0.25) | 0.91 (0.22 to 1.82) | 0.67 (0.17 to 1.36) | -0.14% (-0.45 to 0.17) | 0.87 (0.22 to 1.73) | 0.68 (0.17 to 1.36) | -0.03% (-0.32 to 0.26) |
| Central Sub-Saharan Africa | 298 (67 to 687) | 490 (111 to 977) | 0.64% (0.24 to 1.07) | 0.54 (0.12 to 1.24) | 0.37 (0.08 to 0.74) | -2.79% (-4.92 to -0.62) | 0.5 (0.12 to 1.12) | 0.36 (0.09 to 0.71) | -2.61% (-4.62 to -0.55) |
| East Asia | 5246 (1398 to 10737) | 8529 (2587 to 17264) | 0.63% (0.45 to 1) | 0.43 (0.11 to 0.88) | 0.58 (0.18 to 1.17) | 0.54% (0.14 to 0.94) | 0.43 (0.12 to 0.88) | 0.53 (0.16 to 1.07) | 0.17% (-0.22 to 0.56) |
| Eastern Europe | 3689 (874 to 7420) | 2786 (684 to 5671) | -0.24% (-0.28 to -0.2) | 1.63 (0.39 to 3.28) | 1.33 (0.33 to 2.7) | -0.68% (-0.96 to -0.39) | 1.65 (0.38 to 3.31) | 1.38 (0.32 to 2.82) | -0.64% (-0.85 to -0.42) |
| Eastern Sub-Saharan Africa | 3923 (871 to 10408) | 1939 (474 to 3926) | -0.51% (-0.76 to 0.05) | 2.06 (0.46 to 5.47) | 0.47 (0.11 to 0.95) | -3.15% (-4.5 to -1.79) | 1.9 (0.44 to 5.04) | 0.48 (0.13 to 0.96) | -2.96% (-4.2 to -1.71) |
| High-income Asia Pacific | 1814 (462 to 3628) | 1736 (492 to 3402) | -0.04% (-0.11 to 0.14) | 1.05 (0.27 to 2.09) | 0.93 (0.26 to 1.82) | -0.58% (-0.67 to -0.49) | 1.05 (0.26 to 2.1) | 0.96 (0.24 to 1.97) | -0.46% (-0.57 to -0.36) |
| High-income North America | 3075 (872 to 6173) | 3976 (1223 to 7804) | 0.29% (0.19 to 0.53) | 1.09 (0.31 to 2.2) | 1.09 (0.34 to 2.14) | -0.22% (-0.46 to 0.02) | 1.07 (0.3 to 2.19) | 0.98 (0.28 to 1.98) | -0.67% (-0.95 to -0.39) |
| North Africa and Middle East | 2849 (730 to 5582) | 5179 (1225 to 10655) | 0.82% (0.54 to 1.1) | 0.83 (0.21 to 1.62) | 0.85 (0.2 to 1.75) | 1.66% (1.03 to 2.3) | 0.79 (0.21 to 1.55) | 0.84 (0.2 to 1.73) | 1.8% (1.17 to 2.44) |
| Oceania | 23 (6 to 47) | 54 (13 to 109) | 1.29% (1.02 to 1.51) | 0.36 (0.09 to 0.73) | 0.4 (0.09 to 0.82) | -0.05% (-0.72 to 0.63) | 0.36 (0.09 to 0.72) | 0.41 (0.1 to 0.82) | 0% (-0.67 to 0.67) |
| South Asia | 7370 (1736 to 14987) | 11928 (3028 to 23835) | 0.62% (0.46 to 0.79) | 0.67 (0.16 to 1.37) | 0.66 (0.17 to 1.32) | -0.14% (-0.39 to 0.12) | 0.7 (0.18 to 1.41) | 0.67 (0.17 to 1.33) | -0.21% (-0.43 to 0.01) |
| Southeast Asia | 2625 (643 to 5171) | 3135 (812 to 6238) | 0.19% (-0.01 to 0.36) | 0.56 (0.14 to 1.11) | 0.47 (0.12 to 0.93) | -0.56% (-0.95 to -0.17) | 0.55 (0.15 to 1.08) | 0.47 (0.12 to 0.93) | -0.51% (-0.9 to -0.12) |
| Southern Latin America | 476 (111 to 960) | 616 (148 to 1252) | 0.3% (0.25 to 0.37) | 0.96 (0.22 to 1.94) | 0.92 (0.22 to 1.88) | -0.24% (-0.34 to -0.14) | 0.95 (0.22 to 1.92) | 0.95 (0.23 to 1.96) | -0.1% (-0.19 to -0.01) |
| Southern Sub-Saharan Africa | 261 (67 to 536) | 336 (90 to 676) | 0.29% (0.17 to 0.4) | 0.5 (0.13 to 1.02) | 0.43 (0.11 to 0.86) | -0.42% (-0.59 to -0.26) | 0.51 (0.14 to 1.03) | 0.43 (0.12 to 0.85) | -0.5% (-0.66 to -0.35) |
| Tropical Latin America | 1330 (330 to 2706) | 1679 (463 to 3446) | 0.26% (0.19 to 0.4) | 0.87 (0.22 to 1.77) | 0.75 (0.21 to 1.54) | -0.47% (-0.52 to -0.41) | 0.86 (0.23 to 1.75) | 0.74 (0.2 to 1.51) | -0.42% (-0.47 to -0.36) |
| Western Europe | 3978 (978 to 8117) | 4324 (1157 to 8788) | 0.09% (0.02 to 0.2) | 1.03 (0.25 to 2.11) | 0.99 (0.27 to 2.01) | -0.32% (-0.4 to -0.25) | 1.05 (0.26 to 2.2) | 1.01 (0.25 to 2.13) | -0.3% (-0.37 to -0.22) |
| Western Sub-Saharan Africa | 932 (236 to 1928) | 2027 (536 to 4149) | 1.17% (0.92 to 1.38) | 0.48 (0.12 to 1) | 0.44 (0.12 to 0.91) | -0.19% (-0.66 to 0.29) | 0.51 (0.13 to 1.04) | 0.48 (0.14 to 0.98) | -0.07% (-0.48 to 0.33) |

YLDs, years lived with disability; CR, crude rate; ASR, age-standardized rate; EAPC, estimated annual percentage change; UI, uncertainty interval; CI, confidence interval.

Table S3. Incidence of traumatic shoulder dislocation in 1990 and 2019 for both sexes in 204 countries and territories, with EAPC from 1990 and 2019.

| **Location** | **Number** | | | **CR, per 100k** | | | **ASR, per 100k** | | |
| --- | --- | --- | --- | --- | --- | --- | --- | --- | --- |
|  | **Number in 1990 (95% UI)** | **Number in 2019 (95% UI)** | **Number change (95% UI)** | **CR in 1990 (95% UI)** | **CR in 2019 (95% UI)** | **EAPC of CR (95% CI)** | **ASR in 1990 (95% UI)** | **ASR in 2019 (95% UI)** | **EAPC of ASR (95% CI)** |
| Afghanistan | 13008 (7437 to 24650) | 82501 (38475 to 184430) | 5.34% (3.76 to 6.82) | 113.91 (65.13 to 215.87) | 215.53 (100.51 to 481.82) | 1.8% (-0.07 to 3.7) | 104.98 (62.27 to 198.77) | 273.69 (113.79 to 635.53) | 2.13% (0.33 to 3.96) |
| Albania | 5690 (3934 to 8418) | 3869 (2741 to 5524) | -0.32% (-0.39 to -0.23) | 171.89 (118.84 to 254.33) | 142.24 (100.77 to 203.06) | -1.3% (-2 to -0.6) | 162.61 (113.04 to 242.91) | 154.41 (110.23 to 221.17) | -0.82% (-1.51 to -0.13) |
| Algeria | 16571 (12201 to 22356) | 25246 (18478 to 35021) | 0.52% (0.43 to 0.62) | 65.54 (48.26 to 88.42) | 60.33 (44.16 to 83.69) | -1.84% (-2.65 to -1.03) | 61.43 (45.18 to 82.29) | 59.48 (43.86 to 82.26) | -1.61% (-2.38 to -0.84) |
| American Samoa | 24 (17 to 33) | 28 (19 to 39) | 0.15% (0.07 to 0.23) | 49.43 (35.07 to 68.3) | 49.68 (34.86 to 69.79) | 0.22% (-0.74 to 1.19) | 50.84 (36.4 to 69.53) | 50.68 (35.9 to 70.93) | 0.23% (-0.7 to 1.18) |
| Andorra | 59 (42 to 85) | 94 (66 to 136) | 0.6% (0.5 to 0.7) | 108.55 (76.88 to 157.3) | 113.09 (78.96 to 164.11) | 0.14% (0.11 to 0.18) | 112.89 (79.87 to 163.18) | 119.17 (81.71 to 172.67) | 0.14% (0.09 to 0.19) |
| Angola | 16730 (7564 to 37318) | 10337 (7714 to 13822) | -0.38% (-0.74 to 0.39) | 162.14 (73.31 to 361.67) | 34.3 (25.6 to 45.86) | -6.7% (-9.2 to -4.12) | 143.64 (67.83 to 311.72) | 34.03 (25.5 to 45.08) | -6.23% (-8.61 to -3.78) |
| Antigua and Barbuda | 37 (27 to 52) | 55 (40 to 80) | 0.51% (0.4 to 0.61) | 60.59 (44.44 to 85.38) | 62.63 (44.81 to 90.08) | -0.12% (-0.36 to 0.13) | 58.45 (42.85 to 82.81) | 64.8 (46.52 to 92.79) | 0.1% (-0.17 to 0.37) |
| Argentina | 34828 (25360 to 48171) | 45094 (32790 to 63406) | 0.29% (0.24 to 0.35) | 105.15 (76.57 to 145.44) | 99.95 (72.68 to 140.54) | -0.28% (-0.35 to -0.21) | 104.95 (76.46 to 145.15) | 102.32 (74.25 to 143.83) | -0.2% (-0.27 to -0.14) |
| Armenia | 4356 (3189 to 5925) | 2372 (1738 to 3291) | -0.46% (-0.49 to -0.42) | 127.58 (93.38 to 173.52) | 78.55 (57.57 to 108.97) | -1.75% (-2.03 to -1.47) | 123.61 (90.52 to 168.13) | 85.2 (62.67 to 118.55) | -1.34% (-1.65 to -1.02) |
| Australia | 30309 (21623 to 42935) | 43935 (31217 to 62814) | 0.45% (0.37 to 0.54) | 179.77 (128.25 to 254.65) | 178.83 (127.06 to 255.67) | -0.02% (-0.11 to 0.07) | 185.25 (131.92 to 263.82) | 190.86 (134.78 to 278.41) | 0.09% (0.02 to 0.16) |
| Austria | 10184 (7248 to 14545) | 10297 (7173 to 14957) | 0.01% (-0.04 to 0.07) | 131.1 (93.3 to 187.22) | 115.49 (80.45 to 167.75) | -0.54% (-0.58 to -0.5) | 131.15 (93.3 to 187.33) | 119.52 (81.81 to 174.41) | -0.44% (-0.48 to -0.4) |
| Azerbaijan | 6606 (4950 to 8939) | 8257 (6032 to 11337) | 0.25% (0.18 to 0.32) | 90.11 (67.52 to 121.93) | 80.33 (58.68 to 110.29) | -1.45% (-2.37 to -0.52) | 84.02 (63.15 to 113.23) | 80.94 (59.23 to 111.56) | -1.17% (-2.1 to -0.22) |
| Bahamas | 127 (97 to 165) | 198 (150 to 261) | 0.56% (0.45 to 0.68) | 49.53 (37.82 to 64.27) | 52.48 (39.69 to 69.32) | 0.12% (-0.06 to 0.31) | 47.22 (36.16 to 61.03) | 53.87 (40.82 to 71.51) | 0.35% (0.17 to 0.53) |
| Bahrain | 296 (217 to 398) | 852 (622 to 1169) | 1.88% (1.64 to 2.12) | 58.17 (42.74 to 78.29) | 59.03 (43.1 to 81.05) | 0.28% (0.15 to 0.4) | 52.76 (39.13 to 70.73) | 61.41 (44.78 to 84.54) | 0.61% (0.47 to 0.75) |
| Bangladesh | 45851 (34379 to 60156) | 76982 (57004 to 102505) | 0.68% (0.53 to 0.82) | 42.04 (31.52 to 55.16) | 48.34 (35.79 to 64.36) | -0.65% (-2.11 to 0.83) | 40.86 (31.21 to 53.55) | 46.94 (34.77 to 62.12) | -0.66% (-2.08 to 0.78) |
| Barbados | 118 (89 to 155) | 142 (107 to 192) | 0.2% (0.12 to 0.29) | 46.63 (35.03 to 60.92) | 47.73 (35.78 to 64.48) | -0.14% (-0.32 to 0.04) | 46.33 (34.68 to 60.82) | 52.84 (39.38 to 71.39) | 0.24% (0.06 to 0.42) |
| Belarus | 16494 (12106 to 22592) | 14434 (10358 to 20300) | -0.12% (-0.18 to -0.07) | 157.53 (115.62 to 215.77) | 151.93 (109.02 to 213.67) | 0.2% (-0.12 to 0.52) | 160.09 (116.94 to 219.19) | 158.06 (112.69 to 220.89) | 0.2% (-0.04 to 0.44) |
| Belgium | 11992 (8431 to 17146) | 16057 (11060 to 23633) | 0.34% (0.25 to 0.41) | 120.17 (84.48 to 171.81) | 140.62 (96.86 to 206.96) | 1.07% (0.72 to 1.43) | 124.2 (86.72 to 178.71) | 138.76 (94.17 to 203.31) | 1.08% (0.64 to 1.52) |
| Belize | 131 (95 to 187) | 258 (192 to 350) | 0.98% (0.42 to 1.42) | 70.28 (50.91 to 100.58) | 62.98 (46.9 to 85.33) | -0.51% (-0.94 to -0.09) | 61.7 (45.48 to 87.26) | 60.54 (44.94 to 82.08) | -0.36% (-0.77 to 0.06) |
| Benin | 2015 (1463 to 2752) | 5347 (3852 to 7285) | 1.65% (1.54 to 1.78) | 41.51 (30.15 to 56.72) | 42.21 (30.41 to 57.52) | 0.04% (-0.03 to 0.1) | 45.88 (33.03 to 62.87) | 47.4 (33.91 to 64.74) | 0.1% (0.06 to 0.15) |
| Bermuda | 34 (24 to 47) | 40 (29 to 55) | 0.17% (0.04 to 0.3) | 56.96 (41.04 to 78.43) | 61.84 (45.35 to 86.02) | 0.25% (0.13 to 0.36) | 57.16 (41.13 to 78.36) | 65.52 (47.36 to 92.45) | 0.44% (0.33 to 0.54) |
| Bhutan | 310 (229 to 408) | 489 (361 to 654) | 0.58% (0.44 to 0.73) | 50.62 (37.44 to 66.68) | 64.88 (47.83 to 86.73) | 0.71% (0.31 to 1.12) | 50.69 (37.62 to 67) | 63.86 (47.48 to 85.03) | 0.63% (0.25 to 1.02) |
| Bolivia (Plurinational State of) | 3866 (2903 to 5113) | 6806 (5062 to 9176) | 0.76% (0.61 to 0.91) | 60.2 (45.22 to 79.62) | 56.66 (42.14 to 76.39) | -0.4% (-0.47 to -0.32) | 57.42 (43.36 to 75.87) | 55.74 (41.46 to 75.3) | -0.29% (-0.38 to -0.21) |
| Bosnia and Herzegovina | 7975 (5746 to 11310) | 4454 (3194 to 6266) | -0.44% (-0.57 to -0.35) | 175.75 (126.62 to 249.23) | 134.96 (96.79 to 189.88) | -3.32% (-5.16 to -1.44) | 174.15 (125.54 to 249.3) | 154.76 (109.75 to 220.57) | -2.95% (-4.85 to -1.01) |
| Botswana | 614 (459 to 834) | 1146 (834 to 1539) | 0.87% (0.67 to 1.07) | 47.2 (35.23 to 64.05) | 49 (35.67 to 65.81) | 0.19% (0.11 to 0.26) | 46.98 (34.82 to 63.66) | 48.86 (35.59 to 65.41) | 0.13% (0.05 to 0.21) |
| Brazil | 141791 (100180 to 201168) | 176999 (125516 to 248827) | 0.25% (0.19 to 0.31) | 95.27 (67.31 to 135.16) | 81.69 (57.93 to 114.84) | -0.49% (-0.54 to -0.44) | 93.67 (66.69 to 131.39) | 80.98 (57.03 to 113.44) | -0.43% (-0.48 to -0.37) |
| Brunei Darussalam | 301 (219 to 413) | 453 (329 to 632) | 0.5% (0.42 to 0.58) | 116.38 (84.89 to 159.86) | 103.56 (75.24 to 144.65) | -0.43% (-0.52 to -0.35) | 109.6 (80.47 to 150.61) | 101.41 (73.49 to 141.4) | -0.32% (-0.4 to -0.24) |
| Bulgaria | 15385 (11187 to 21134) | 9830 (6989 to 13908) | -0.36% (-0.42 to -0.31) | 177.22 (128.87 to 243.45) | 141.76 (100.79 to 200.56) | -0.84% (-0.89 to -0.79) | 189.12 (136.89 to 259.67) | 166.88 (119.69 to 236.4) | -0.49% (-0.57 to -0.42) |
| Burkina Faso | 3775 (2741 to 5127) | 13366 (9392 to 19586) | 2.54% (1.87 to 4.05) | 39.49 (28.67 to 53.64) | 58.9 (41.39 to 86.31) | 0.7% (0.38 to 1.01) | 44.37 (31.94 to 59.93) | 64.08 (45.19 to 90.47) | 0.72% (0.45 to 0.99) |
| Burundi | 2478 (1871 to 3264) | 5547 (4177 to 7408) | 1.24% (1.1 to 1.48) | 44.49 (33.59 to 58.6) | 46.48 (35 to 62.07) | -7.35% (-12.05 to -2.4) | 45.13 (34.06 to 59.09) | 45.51 (34.51 to 60.24) | -7.13% (-11.62 to -2.4) |
| Cabo Verde | 147 (108 to 196) | 277 (201 to 367) | 0.88% (0.73 to 1.04) | 41.85 (30.77 to 55.72) | 49.21 (35.67 to 65.18) | 0.69% (0.6 to 0.78) | 42.51 (31.05 to 56.53) | 49.89 (36.43 to 66.06) | 0.65% (0.6 to 0.71) |
| Cambodia | 6126 (4292 to 9228) | 8869 (6416 to 12425) | 0.45% (-0.03 to 0.9) | 59.04 (41.37 to 88.94) | 53.42 (38.65 to 74.83) | -0.94% (-1.59 to -0.29) | 56.51 (40.32 to 83.04) | 53.67 (38.83 to 74.91) | -0.75% (-1.35 to -0.16) |
| Cameroon | 4603 (3323 to 6338) | 16470 (11755 to 23122) | 2.58% (2.11 to 3.59) | 44.3 (31.98 to 61) | 56.6 (40.39 to 79.45) | 1.25% (0.77 to 1.73) | 49.73 (35.74 to 70.06) | 61.32 (43.51 to 84.53) | 1.08% (0.7 to 1.46) |
| Canada | 26048 (19124 to 35846) | 37217 (27261 to 52125) | 0.43% (0.32 to 0.54) | 95.56 (70.16 to 131.51) | 101.91 (74.65 to 142.73) | 0.27% (0.21 to 0.33) | 92.85 (68.24 to 127.88) | 90.94 (66.9 to 126.7) | -0.07% (-0.12 to -0.02) |
| Central African Republic | 862 (654 to 1133) | 3257 (2037 to 5794) | 2.78% (1.48 to 5.96) | 31.4 (23.84 to 41.27) | 61.46 (38.43 to 109.33) | 5.66% (3.37 to 8.01) | 31.35 (23.64 to 40.72) | 57.51 (37.16 to 99.37) | 5.26% (3.08 to 7.5) |
| Chad | 5149 (2872 to 10054) | 7914 (5700 to 10865) | 0.54% (-0.04 to 1.36) | 85.5 (47.69 to 166.95) | 48.26 (34.76 to 66.26) | -1.38% (-2.64 to -0.1) | 85.2 (49.37 to 159.37) | 53.39 (38.65 to 72.84) | -1.03% (-2.15 to 0.11) |
| Chile | 13477 (9806 to 18651) | 18658 (13513 to 26202) | 0.38% (0.31 to 0.46) | 101.49 (73.84 to 140.44) | 102.52 (74.25 to 143.98) | -0.06% (-0.29 to 0.17) | 98.67 (71.75 to 136.42) | 106.87 (77.57 to 151.41) | 0.26% (0.03 to 0.5) |
| China | 553401 (385559 to 789270) | 900891 (633673 to 1229159) | 0.63% (0.45 to 0.83) | 46.75 (32.57 to 66.68) | 63.34 (44.55 to 86.42) | 0.54% (0.13 to 0.95) | 47.1 (33.21 to 66.4) | 58.04 (41.01 to 79.51) | 0.19% (-0.2 to 0.59) |
| Colombia | 33520 (24689 to 46496) | 28684 (21030 to 38759) | -0.14% (-0.35 to 0) | 102.99 (75.85 to 142.86) | 60.04 (44.02 to 81.13) | -2.31% (-2.64 to -1.99) | 95.99 (71.24 to 132.67) | 62.55 (45.95 to 84.97) | -1.93% (-2.23 to -1.63) |
| Comoros | 272 (193 to 372) | 436 (308 to 597) | 0.6% (0.52 to 0.69) | 58.41 (41.45 to 79.82) | 61.06 (43.15 to 83.51) | -0.17% (-0.62 to 0.28) | 61.91 (43.8 to 84.69) | 62.13 (44.13 to 85.23) | -0.29% (-0.69 to 0.11) |
| Congo | 806 (599 to 1062) | 1678 (1245 to 2247) | 1.08% (0.99 to 1.18) | 32.97 (24.51 to 43.42) | 31.87 (23.65 to 42.66) | -2.96% (-6.28 to 0.48) | 32.9 (24.66 to 43.05) | 31.78 (23.77 to 42.33) | -2.81% (-5.99 to 0.47) |
| Cook Islands | 10 (7 to 13) | 10 (7 to 14) | -0.01% (-0.12 to 0.09) | 51.87 (37.36 to 70.26) | 54.25 (37.89 to 76.7) | -0.14% (-1.03 to 0.76) | 54.01 (39.36 to 72.66) | 54.78 (38.22 to 78.16) | -0.21% (-1.18 to 0.77) |
| Costa Rica | 2181 (1565 to 3007) | 3108 (2280 to 4293) | 0.42% (0.35 to 0.5) | 71.76 (51.47 to 98.92) | 65.89 (48.34 to 91.01) | -0.3% (-0.41 to -0.19) | 68.34 (50.33 to 93.5) | 69.01 (50.11 to 95.24) | 0.06% (-0.04 to 0.17) |
| Côte d'Ivoire | 5278 (3835 to 7196) | 11485 (8270 to 15509) | 1.18% (1.1 to 1.26) | 43.17 (31.37 to 58.85) | 43.89 (31.6 to 59.26) | 0.11% (-0.56 to 0.79) | 49.37 (35.85 to 67.12) | 49.25 (35.83 to 66.87) | 0.02% (-0.54 to 0.59) |
| Croatia | 8941 (6469 to 12232) | 7214 (5118 to 10218) | -0.19% (-0.31 to -0.08) | 182.44 (132 to 249.6) | 169.82 (120.47 to 240.54) | -0.98% (-1.47 to -0.49) | 185.05 (133.41 to 251.13) | 169.13 (119.38 to 238.98) | -1.02% (-1.47 to -0.56) |
| Cuba | 7516 (5700 to 9795) | 9536 (7104 to 13008) | 0.27% (0.14 to 0.4) | 69.38 (52.62 to 90.42) | 83.95 (62.55 to 114.52) | 0.69% (0.61 to 0.77) | 67.82 (51.62 to 88.6) | 82.41 (61.59 to 111.38) | 0.71% (0.65 to 0.77) |
| Cyprus | 797 (566 to 1136) | 1386 (968 to 2025) | 0.74% (0.62 to 0.86) | 102.45 (72.72 to 146) | 105.56 (73.7 to 154.15) | 0.09% (0.02 to 0.17) | 104.39 (74.06 to 148.18) | 114.18 (78.15 to 166.38) | 0.3% (0.25 to 0.35) |
| Czechia | 19986 (14524 to 27556) | 17101 (12340 to 24194) | -0.14% (-0.18 to -0.1) | 194.08 (141.04 to 267.6) | 160.68 (115.94 to 227.32) | -0.63% (-0.71 to -0.54) | 192.06 (139.68 to 264.09) | 174.18 (124.07 to 248.73) | -0.18% (-0.25 to -0.11) |
| Democratic People's Republic of Korea | 6268 (4568 to 8584) | 6642 (4954 to 8775) | 0.06% (-0.05 to 0.19) | 29.77 (21.7 to 40.77) | 25.32 (18.88 to 33.45) | -0.39% (-0.62 to -0.17) | 29.67 (21.68 to 40.51) | 24.62 (18.44 to 32.52) | -0.5% (-0.71 to -0.29) |
| Democratic Republic of the Congo | 13484 (10179 to 17949) | 36947 (27382 to 52230) | 1.74% (1.41 to 2.4) | 34.94 (26.38 to 46.51) | 42.14 (31.23 to 59.58) | -0.98% (-3.37 to 1.46) | 33.97 (25.69 to 44.28) | 40.57 (30.31 to 55.76) | -0.95% (-3.2 to 1.35) |
| Denmark | 6090 (4378 to 8741) | 5945 (4158 to 8666) | -0.02% (-0.08 to 0.03) | 118.38 (85.1 to 169.92) | 102.45 (71.66 to 149.34) | -0.7% (-0.81 to -0.59) | 115.07 (81.52 to 165.09) | 110.59 (75.66 to 163.3) | -0.24% (-0.35 to -0.14) |
| Djibouti | 506 (296 to 936) | 658 (472 to 898) | 0.3% (-0.32 to 1.09) | 104.15 (60.95 to 192.49) | 54.71 (39.22 to 74.65) | -2.55% (-3.92 to -1.15) | 101.24 (61.71 to 177.74) | 57.38 (41.47 to 77.42) | -2.34% (-3.6 to -1.06) |
| Dominica | 36 (27 to 47) | 35 (26 to 46) | -0.04% (-0.09 to 0.02) | 48.81 (36.84 to 63.07) | 50.62 (38.08 to 67.02) | 0.54% (0.07 to 1.01) | 47.15 (35.74 to 61.25) | 52.66 (39.49 to 70.63) | 0.71% (0.3 to 1.13) |
| Dominican Republic | 3676 (2779 to 4816) | 6741 (5058 to 8906) | 0.83% (0.71 to 0.94) | 51.04 (38.57 to 66.86) | 61.95 (46.48 to 81.85) | 0.69% (0.52 to 0.87) | 47.52 (35.97 to 61.67) | 60.84 (45.69 to 80.53) | 0.86% (0.69 to 1.04) |
| Ecuador | 6418 (4810 to 8479) | 11824 (8746 to 16005) | 0.84% (0.71 to 0.97) | 64.01 (47.97 to 84.56) | 67.23 (49.73 to 91) | 0.07% (-0.08 to 0.23) | 60.99 (45.8 to 80.78) | 66.01 (48.95 to 89.39) | 0.18% (0.03 to 0.33) |
| Egypt | 25426 (18762 to 34038) | 52070 (38228 to 71293) | 1.05% (0.89 to 1.25) | 45.65 (33.68 to 61.11) | 52.56 (38.59 to 71.96) | 0.44% (0.2 to 0.68) | 42.45 (31.6 to 56.53) | 50.19 (36.96 to 68.1) | 0.53% (0.28 to 0.78) |
| El Salvador | 8921 (5001 to 17348) | 4137 (3018 to 5591) | -0.54% (-0.77 to -0.18) | 169.36 (94.93 to 329.34) | 66.12 (48.24 to 89.37) | -1.47% (-2.36 to -0.58) | 150.91 (86.09 to 288.31) | 65.79 (48.11 to 89.28) | -1.16% (-2.03 to -0.29) |
| Equatorial Guinea | 144 (109 to 191) | 484 (359 to 644) | 2.35% (2.06 to 2.67) | 33.51 (25.42 to 44.29) | 34.06 (25.26 to 45.35) | 0.07% (-0.13 to 0.27) | 32.79 (24.72 to 42.73) | 33.28 (24.79 to 44.2) | 0.05% (-0.13 to 0.24) |
| Eritrea | 33682 (9434 to 86304) | 3643 (2596 to 4975) | -0.89% (-0.96 to -0.61) | 1122.21 (314.31 to 2875.45) | 54.28 (38.68 to 74.12) | -4.78% (-8.1 to -1.33) | 999.62 (288.17 to 2542.97) | 57.64 (41.34 to 78.46) | -4.65% (-7.81 to -1.37) |
| Estonia | 2908 (2130 to 4023) | 1671 (1215 to 2342) | -0.43% (-0.46 to -0.39) | 185.38 (135.8 to 256.47) | 127.34 (92.61 to 178.43) | -1.44% (-1.55 to -1.32) | 187.17 (137.17 to 259.13) | 138.68 (100.23 to 194.54) | -1.19% (-1.29 to -1.09) |
| Eswatini | 332 (242 to 449) | 532 (388 to 712) | 0.6% (0.5 to 0.72) | 41.16 (29.95 to 55.66) | 46.55 (33.93 to 62.38) | 0.49% (0.43 to 0.55) | 42.34 (30.91 to 56.66) | 46.75 (34.24 to 62.98) | 0.37% (0.28 to 0.47) |
| Ethiopia | 288039 (109288 to 683779) | 46611 (34840 to 62055) | -0.84% (-0.93 to -0.55) | 560.48 (212.66 to 1330.53) | 43.32 (32.38 to 57.68) | -3.8% (-6.4 to -1.12) | 505.88 (194.58 to 1190.52) | 42.57 (32 to 55.48) | -3.75% (-6.21 to -1.23) |
| Fiji | 265 (194 to 358) | 333 (239 to 470) | 0.26% (0.17 to 0.34) | 34.85 (25.53 to 47.12) | 36.53 (26.25 to 51.55) | 0.08% (-0.19 to 0.35) | 33.46 (24.76 to 44.95) | 36.43 (26.25 to 51.14) | 0.21% (-0.06 to 0.49) |
| Finland | 8102 (5659 to 11665) | 8553 (5841 to 12613) | 0.06% (0 to 0.11) | 161.71 (112.95 to 232.84) | 154.55 (105.55 to 227.91) | -0.13% (-0.48 to 0.23) | 166.22 (114.36 to 238.43) | 163.29 (109.19 to 245) | 0.01% (-0.35 to 0.37) |
| France | 76896 (54745 to 109570) | 86926 (61023 to 127389) | 0.13% (0.06 to 0.19) | 133.11 (94.76 to 189.67) | 131.3 (92.17 to 192.42) | -0.11% (-0.14 to -0.08) | 131.78 (93.65 to 186.55) | 128.15 (88.38 to 186.2) | -0.16% (-0.19 to -0.12) |
| Gabon | 365 (271 to 483) | 634 (471 to 839) | 0.74% (0.66 to 0.83) | 36.78 (27.3 to 48.75) | 36.23 (26.93 to 47.94) | -0.14% (-0.25 to -0.04) | 36.55 (27.35 to 48.06) | 36.58 (27.47 to 48.25) | -0.1% (-0.2 to -0.01) |
| Gambia | 380 (278 to 520) | 908 (661 to 1222) | 1.39% (1.28 to 1.49) | 38.35 (27.99 to 52.43) | 40.44 (29.43 to 54.41) | 0.2% (0.06 to 0.35) | 41.98 (30.45 to 56.8) | 44.66 (32.34 to 59.99) | 0.23% (0.11 to 0.36) |
| Georgia | 6426 (4672 to 8869) | 4313 (3091 to 5956) | -0.33% (-0.36 to -0.3) | 116.65 (84.82 to 161) | 117.68 (84.35 to 162.51) | -0.39% (-1.13 to 0.35) | 118.04 (85.94 to 163.16) | 129.03 (92.16 to 177.99) | -0.16% (-0.92 to 0.61) |
| Germany | 83799 (59516 to 120115) | 90930 (63452 to 131649) | 0.09% (0.03 to 0.14) | 104.83 (74.45 to 150.25) | 107.08 (74.72 to 155.04) | -0.07% (-0.23 to 0.09) | 109 (77.14 to 156.05) | 111.64 (77.27 to 163.95) | -0.04% (-0.17 to 0.09) |
| Ghana | 6410 (4590 to 8815) | 15938 (11232 to 22312) | 1.49% (1.33 to 1.63) | 42.68 (30.57 to 58.7) | 50.54 (35.62 to 70.75) | 0.63% (0.55 to 0.7) | 46.46 (33.14 to 63.64) | 53.78 (38.11 to 74.96) | 0.53% (0.46 to 0.6) |
| Greece | 10915 (7822 to 15576) | 9024 (6393 to 12971) | -0.17% (-0.21 to -0.13) | 105.06 (75.29 to 149.92) | 87.29 (61.84 to 125.48) | -0.78% (-0.85 to -0.71) | 112.05 (79.65 to 158.81) | 102.53 (70.63 to 149.9) | -0.41% (-0.46 to -0.36) |
| Greenland | 94 (67 to 135) | 76 (54 to 109) | -0.19% (-0.25 to -0.13) | 169.69 (120.88 to 242.7) | 135.83 (96.13 to 193.92) | -0.82% (-0.94 to -0.71) | 171.83 (123.41 to 242.15) | 130.04 (91.98 to 185.28) | -1.11% (-1.18 to -1.04) |
| Grenada | 47 (35 to 63) | 66 (48 to 91) | 0.4% (0.29 to 0.51) | 54.73 (40.72 to 73.19) | 63.7 (46.93 to 87.72) | 0.34% (0.08 to 0.6) | 52.85 (39.69 to 70.85) | 64.94 (47.83 to 89.91) | 0.55% (0.28 to 0.81) |
| Guam | 68 (48 to 93) | 84 (59 to 117) | 0.24% (0.14 to 0.34) | 49.47 (34.86 to 68.16) | 49.14 (34.41 to 68.71) | -0.1% (-0.25 to 0.04) | 48.48 (34.36 to 66.69) | 49.68 (34.71 to 69.51) | 0.01% (-0.13 to 0.14) |
| Guatemala | 10032 (6556 to 16961) | 14440 (10537 to 20257) | 0.44% (-0.15 to 1.03) | 125.93 (82.29 to 212.9) | 81.23 (59.27 to 113.96) | -0.8% (-1.15 to -0.45) | 113.31 (74.32 to 188.93) | 78.21 (57.07 to 109.58) | -0.6% (-0.94 to -0.27) |
| Guinea | 2580 (1877 to 3501) | 5489 (3987 to 7503) | 1.13% (1.02 to 1.24) | 41.7 (30.34 to 56.59) | 43.41 (31.54 to 59.35) | 0.01% (-0.69 to 0.71) | 44.12 (31.88 to 60.54) | 48.52 (35.06 to 66.84) | 0.24% (-0.4 to 0.87) |
| Guinea-Bissau | 480 (345 to 666) | 898 (634 to 1251) | 0.87% (0.79 to 0.96) | 47.62 (34.2 to 66.13) | 47.24 (33.33 to 65.78) | -0.69% (-2.11 to 0.76) | 52.12 (36.89 to 71.83) | 51.48 (36.36 to 71.55) | -0.63% (-1.9 to 0.66) |
| Guyana | 522 (398 to 683) | 559 (423 to 732) | 0.07% (-0.01 to 0.15) | 67.79 (51.63 to 88.65) | 72.54 (54.91 to 94.92) | 0.09% (-0.2 to 0.37) | 64.23 (49.2 to 83.98) | 71.63 (54.54 to 93.78) | 0.23% (-0.05 to 0.52) |
| Haiti | 3592 (2732 to 4882) | 6647 (4973 to 9046) | 0.85% (0.73 to 0.97) | 56.51 (42.98 to 76.81) | 53.59 (40.1 to 72.94) | 0.17% (-2.35 to 2.76) | 53.67 (40.88 to 72.59) | 51.64 (38.9 to 70.84) | 0.25% (-2.31 to 2.88) |
| Honduras | 3391 (2530 to 4636) | 6395 (4733 to 8757) | 0.89% (0.75 to 1.03) | 72.02 (53.72 to 98.47) | 65.16 (48.22 to 89.22) | -1.09% (-2.35 to 0.18) | 63.84 (48.2 to 86.26) | 61.93 (46.21 to 84.08) | -0.86% (-2.15 to 0.45) |
| Hungary | 20195 (14445 to 28269) | 14854 (10548 to 21092) | -0.26% (-0.3 to -0.23) | 194.32 (138.99 to 272) | 153.54 (109.03 to 218.01) | -1.14% (-1.25 to -1.02) | 189.57 (137.6 to 263.47) | 163.71 (115.9 to 234.2) | -0.78% (-0.9 to -0.65) |
| Iceland | 272 (192 to 384) | 364 (253 to 527) | 0.34% (0.27 to 0.4) | 107.18 (75.58 to 151.09) | 105.69 (73.45 to 152.67) | -0.15% (-0.27 to -0.04) | 107.42 (75.5 to 152.09) | 111.42 (76.36 to 163.07) | 0.02% (-0.09 to 0.13) |
| India | 702230 (521057 to 947285) | 1095416 (797889 to 1503652) | 0.56% (0.39 to 0.71) | 82.08 (60.9 to 110.72) | 78.77 (57.37 to 108.12) | -0.1% (-0.2 to 0.01) | 85.38 (63.58 to 115.5) | 79.64 (58.16 to 109.1) | -0.21% (-0.3 to -0.11) |
| Indonesia | 106197 (77190 to 144238) | 131991 (94919 to 178229) | 0.24% (0.2 to 0.29) | 57.29 (41.64 to 77.81) | 50.87 (36.58 to 68.69) | -0.59% (-0.88 to -0.3) | 58.19 (42.25 to 78.96) | 51.56 (37.17 to 69.79) | -0.6% (-0.89 to -0.32) |
| Iran (Islamic Republic of) | 78292 (56472 to 111562) | 49124 (35824 to 66741) | -0.37% (-0.58 to -0.16) | 133.74 (96.47 to 190.57) | 58.27 (42.5 to 79.17) | -1.43% (-1.91 to -0.94) | 136.6 (96.34 to 197.24) | 57.75 (42.29 to 78.49) | -1.35% (-1.9 to -0.79) |
| Iraq | 24370 (16117 to 37169) | 41995 (30122 to 59534) | 0.72% (0.41 to 1.04) | 138.49 (91.59 to 211.23) | 99.7 (71.52 to 141.35) | 4.07% (1.78 to 6.41) | 130.29 (87.91 to 196.05) | 91.65 (66.15 to 129.41) | 3.86% (1.53 to 6.24) |
| Ireland | 3799 (2686 to 5384) | 5088 (3538 to 7405) | 0.34% (0.25 to 0.42) | 105.49 (74.58 to 149.5) | 103.62 (72.06 to 150.8) | -0.15% (-0.25 to -0.04) | 106.95 (75.76 to 151.94) | 111.98 (77.09 to 164.53) | 0.06% (-0.04 to 0.15) |
| Israel | 5207 (3747 to 7331) | 9066 (6285 to 13136) | 0.74% (0.5 to 0.93) | 104.94 (75.51 to 147.75) | 97.38 (67.51 to 141.1) | 0.02% (-0.68 to 0.72) | 102.63 (73.96 to 143.66) | 98.97 (68.29 to 144.25) | 0.16% (-0.54 to 0.87) |
| Italy | 66111 (46892 to 93993) | 54082 (37695 to 79173) | -0.18% (-0.24 to -0.13) | 116.4 (82.56 to 165.49) | 89.67 (62.5 to 131.27) | -1.75% (-2.1 to -1.4) | 116.81 (83.69 to 164.54) | 93.5 (64.15 to 137.07) | -1.6% (-1.97 to -1.23) |
| Jamaica | 1480 (1102 to 2006) | 1588 (1168 to 2205) | 0.07% (-0.05 to 0.18) | 62.62 (46.62 to 84.87) | 56.51 (41.54 to 78.44) | -0.36% (-0.48 to -0.25) | 58.84 (44 to 80.43) | 57.23 (42.01 to 79.45) | -0.13% (-0.24 to -0.01) |
| Japan | 133340 (97138 to 187572) | 122573 (89176 to 172201) | -0.08% (-0.13 to -0.02) | 105.94 (77.18 to 149.02) | 95.92 (69.78 to 134.75) | -0.44% (-0.54 to -0.35) | 107.43 (77.59 to 152.32) | 99.83 (71.31 to 142.85) | -0.34% (-0.45 to -0.22) |
| Jordan | 2201 (1616 to 2996) | 6703 (4834 to 9262) | 2.05% (1.81 to 2.3) | 58.32 (42.82 to 79.41) | 57.61 (41.54 to 79.59) | 0.11% (-0.29 to 0.5) | 52.51 (39.04 to 70.71) | 53.95 (39.16 to 74) | 0.25% (-0.14 to 0.64) |
| Kazakhstan | 18672 (13648 to 25805) | 19551 (14191 to 27047) | 0.05% (0.01 to 0.09) | 114.08 (83.38 to 157.66) | 106.3 (77.16 to 147.06) | 0.05% (-0.09 to 0.18) | 109.72 (80.34 to 151.34) | 106.27 (76.89 to 145.83) | 0.16% (0.05 to 0.27) |
| Kenya | 9827 (7263 to 13156) | 21572 (16141 to 28731) | 1.2% (1.12 to 1.27) | 42.37 (31.31 to 56.72) | 42.95 (32.14 to 57.2) | 0.21% (-0.16 to 0.58) | 41.76 (31.49 to 55.13) | 42.69 (32.15 to 56.27) | 0.2% (-0.12 to 0.52) |
| Kiribati | 21 (15 to 28) | 37 (26 to 51) | 0.79% (0.67 to 0.91) | 27.7 (20.36 to 37.16) | 30.9 (22.14 to 43) | -0.62% (-1.43 to 0.21) | 26.35 (19.41 to 35.16) | 29.47 (21.28 to 40.56) | -0.59% (-1.35 to 0.19) |
| Kuwait | 6137 (2772 to 13772) | 3141 (2256 to 4407) | -0.49% (-0.78 to 0.2) | 348.79 (157.57 to 782.73) | 70.95 (50.97 to 99.56) | -2.15% (-3.62 to -0.65) | 306.21 (137.22 to 680.04) | 67.81 (49.19 to 95.05) | -2.21% (-3.9 to -0.49) |
| Kyrgyzstan | 4811 (3498 to 6602) | 5410 (3966 to 7458) | 0.12% (0.05 to 0.2) | 107.81 (78.39 to 147.97) | 82.79 (60.68 to 114.11) | -1.05% (-1.32 to -0.78) | 102.95 (75.63 to 140.78) | 79.45 (58.6 to 108.93) | -1.02% (-1.29 to -0.75) |
| Lao People's Democratic Republic | 2908 (1794 to 5412) | 2903 (2160 to 3833) | 0% (-0.46 to 0.52) | 70.04 (43.2 to 130.36) | 40.55 (30.18 to 53.55) | -0.52% (-0.91 to -0.12) | 65.92 (42.21 to 117.11) | 39.72 (29.75 to 52.49) | -0.53% (-0.89 to -0.17) |
| Latvia | 5520 (4012 to 7685) | 2526 (1841 to 3515) | -0.54% (-0.56 to -0.52) | 207.62 (150.91 to 289.06) | 131.88 (96.11 to 183.55) | -1.69% (-1.86 to -1.52) | 207.6 (151.63 to 288.06) | 141.28 (102.29 to 196.46) | -1.49% (-1.63 to -1.35) |
| Lebanon | 5098 (2689 to 10450) | 3136 (2263 to 4386) | -0.38% (-0.7 to 0.15) | 155.67 (82.1 to 319.07) | 60.57 (43.72 to 84.72) | -0.7% (-1.67 to 0.28) | 146.67 (78.7 to 300.53) | 61.01 (43.98 to 84.98) | -0.56% (-1.52 to 0.4) |
| Lesotho | 767 (569 to 1033) | 1056 (771 to 1405) | 0.38% (0.27 to 0.49) | 42.45 (31.49 to 57.15) | 50.51 (36.88 to 67.19) | 0.65% (0.31 to 0.99) | 42.62 (31.42 to 56.64) | 50.12 (36.54 to 66.87) | 0.6% (0.29 to 0.9) |
| Liberia | 14406 (4191 to 37283) | 1818 (1324 to 2488) | -0.87% (-0.95 to -0.56) | 733.4 (213.35 to 1898.1) | 37.96 (27.64 to 51.93) | -8.04% (-11.05 to -4.93) | 690.88 (205.56 to 1737.24) | 41.22 (30 to 55.6) | -7.5% (-10.37 to -4.53) |
| Libya | 2599 (1922 to 3508) | 7827 (5109 to 13334) | 2.01% (1.1 to 4.21) | 61.34 (45.37 to 82.8) | 116.2 (75.85 to 197.97) | 3.32% (1.07 to 5.62) | 58.96 (43.34 to 80.13) | 113.5 (73.18 to 196.64) | 3.33% (1.08 to 5.62) |
| Lithuania | 7086 (5218 to 9744) | 4091 (2962 to 5688) | -0.42% (-0.45 to -0.39) | 192.9 (142.04 to 265.24) | 146.4 (106.02 to 203.55) | -0.9% (-1.03 to -0.76) | 190.97 (140.3 to 261.99) | 150.51 (109.65 to 209.45) | -0.8% (-0.9 to -0.69) |
| Luxembourg | 511 (366 to 720) | 717 (506 to 1045) | 0.4% (0.28 to 0.51) | 133.96 (96.01 to 188.79) | 115.99 (81.8 to 168.91) | -0.69% (-0.76 to -0.63) | 137.75 (99.17 to 194.17) | 121.43 (83.07 to 176.65) | -0.66% (-0.74 to -0.57) |
| Madagascar | 6511 (4705 to 8953) | 14552 (10705 to 19870) | 1.23% (1.11 to 1.43) | 54.48 (39.37 to 74.91) | 54.52 (40.11 to 74.45) | -0.03% (-0.2 to 0.15) | 56.06 (40.18 to 77.07) | 55.55 (40.68 to 76.13) | -0.06% (-0.2 to 0.08) |
| Malawi | 4368 (3140 to 5997) | 8885 (6400 to 12093) | 1.03% (0.94 to 1.14) | 45.71 (32.86 to 62.76) | 48.18 (34.7 to 65.57) | 0.03% (-0.13 to 0.19) | 47.55 (34.64 to 65.11) | 49.13 (35.99 to 66.83) | -0.03% (-0.17 to 0.11) |
| Malaysia | 7585 (5621 to 9907) | 14854 (10914 to 19692) | 0.96% (0.83 to 1.1) | 42.96 (31.84 to 56.12) | 47.45 (34.87 to 62.91) | 0.21% (0.08 to 0.33) | 42.84 (32.08 to 56.19) | 47.32 (34.88 to 63.1) | 0.17% (0.05 to 0.29) |
| Maldives | 91 (67 to 123) | 218 (160 to 299) | 1.4% (1.15 to 1.69) | 40.9 (30.37 to 55.34) | 43.8 (32.16 to 59.89) | 0.06% (-0.68 to 0.81) | 40.68 (30.54 to 54.41) | 43.41 (31.97 to 59.16) | 0.04% (-0.7 to 0.79) |
| Mali | 6030 (3819 to 10344) | 13132 (8795 to 20703) | 1.18% (0.96 to 1.41) | 69.53 (44.03 to 119.27) | 59.91 (40.13 to 94.46) | -1.76% (-4.05 to 0.57) | 71.09 (45.97 to 117.81) | 62.93 (43.17 to 96.33) | -1.55% (-3.65 to 0.6) |
| Malta | 408 (286 to 586) | 485 (334 to 705) | 0.19% (0.12 to 0.26) | 110 (77.03 to 158.01) | 110.37 (75.99 to 160.5) | 0.2% (0.13 to 0.27) | 115.21 (80.21 to 164.95) | 125.26 (84.48 to 184.31) | 0.58% (0.49 to 0.68) |
| Marshall Islands | 16 (12 to 21) | 22 (16 to 30) | 0.41% (0.33 to 0.51) | 34.43 (25.39 to 46.44) | 39.19 (28.62 to 53.43) | 0.46% (0.39 to 0.53) | 34.94 (25.79 to 46.55) | 38.71 (28.32 to 52.72) | 0.34% (0.26 to 0.43) |
| Mauritania | 1145 (815 to 1584) | 1877 (1333 to 2615) | 0.64% (0.36 to 0.82) | 55.39 (39.45 to 76.65) | 46.75 (33.21 to 65.15) | -0.22% (-0.44 to 0) | 59.26 (41.32 to 82.67) | 50.23 (35.72 to 70.35) | -0.25% (-0.46 to -0.04) |
| Mauritius | 484 (351 to 662) | 682 (491 to 921) | 0.41% (0.31 to 0.52) | 44.04 (31.94 to 60.18) | 53.44 (38.48 to 72.12) | 0.69% (0.62 to 0.76) | 43.23 (31.38 to 58.83) | 53.29 (38.37 to 72.76) | 0.74% (0.68 to 0.8) |
| Mexico | 84448 (59805 to 120403) | 101086 (71632 to 141657) | 0.2% (0.14 to 0.26) | 98.78 (69.96 to 140.84) | 80.91 (57.33 to 113.38) | 1.21% (0.67 to 1.75) | 97.93 (69.94 to 137.16) | 80.91 (57.37 to 113.71) | 1.11% (0.62 to 1.61) |
| Micronesia (Federated States of) | 36 (27 to 49) | 42 (30 to 59) | 0.16% (0.07 to 0.25) | 34.82 (25.69 to 46.68) | 41.15 (29.67 to 57.46) | 0.54% (-0.11 to 1.19) | 35.24 (26.14 to 47.01) | 40.84 (29.63 to 56.81) | 0.47% (-0.17 to 1.11) |
| Monaco | 29 (20 to 41) | 40 (28 to 57) | 0.39% (0.32 to 0.45) | 95.03 (66.27 to 134.74) | 106.98 (73.89 to 152.71) | 0.44% (0.4 to 0.49) | 99.74 (67.98 to 147.63) | 113.5 (75.81 to 169.31) | 0.5% (0.43 to 0.56) |
| Mongolia | 2072 (1557 to 2821) | 3743 (2731 to 5163) | 0.81% (0.68 to 0.92) | 96.22 (72.3 to 131) | 110.48 (80.6 to 152.4) | 0.67% (0.45 to 0.88) | 91.16 (68.08 to 124.45) | 107.12 (78.28 to 147.31) | 0.71% (0.52 to 0.91) |
| Montenegro | 1029 (745 to 1431) | 941 (673 to 1327) | -0.09% (-0.12 to -0.05) | 164.42 (119.05 to 228.77) | 151.74 (108.45 to 213.97) | -0.32% (-0.39 to -0.25) | 163.36 (118.26 to 227.67) | 162.76 (116.52 to 232.84) | -0.07% (-0.15 to 0.01) |
| Morocco | 17574 (12876 to 23994) | 24592 (17785 to 34260) | 0.4% (0.28 to 0.52) | 69.47 (50.9 to 94.85) | 68.4 (49.47 to 95.29) | -0.23% (-0.33 to -0.13) | 64.52 (47.33 to 87.65) | 67.42 (48.87 to 94.33) | -0.02% (-0.13 to 0.08) |
| Mozambique | 15353 (8251 to 30513) | 19845 (14643 to 26924) | 0.29% (-0.32 to 1.2) | 117.46 (63.13 to 233.44) | 67.21 (49.59 to 91.18) | -0.68% (-1.47 to 0.11) | 116.26 (64.87 to 223.61) | 72.38 (53.05 to 98.73) | -0.46% (-1.16 to 0.26) |
| Myanmar | 26424 (19694 to 35318) | 31687 (23460 to 43183) | 0.2% (0.09 to 0.3) | 64.29 (47.91 to 85.92) | 57.95 (42.91 to 78.98) | -0.11% (-1.5 to 1.29) | 60.79 (45.56 to 80.83) | 58.09 (43.02 to 78.95) | 0.1% (-1.29 to 1.52) |
| Namibia | 625 (452 to 840) | 1140 (828 to 1522) | 0.83% (0.72 to 0.93) | 44.3 (32.09 to 59.58) | 47.46 (34.48 to 63.33) | 0.02% (-0.37 to 0.42) | 45.95 (33.27 to 61.82) | 47.94 (34.71 to 63.73) | -0.09% (-0.45 to 0.28) |
| Nauru | 5 (3 to 6) | 5 (4 to 8) | 0.22% (0.14 to 0.31) | 43.97 (31.25 to 60.2) | 52.08 (36.38 to 72.77) | 0.52% (0.45 to 0.59) | 46.27 (32.8 to 62.89) | 52.61 (37.01 to 72.8) | 0.37% (0.3 to 0.45) |
| Nepal | 13015 (9458 to 18009) | 21981 (15693 to 30640) | 0.69% (0.6 to 0.78) | 66.62 (48.41 to 92.18) | 72.27 (51.59 to 100.74) | 0.15% (-0.76 to 1.07) | 67.68 (49.12 to 93.1) | 71.02 (50.88 to 99.6) | 0.05% (-0.8 to 0.9) |
| Netherlands | 12458 (8941 to 17681) | 17541 (12431 to 25308) | 0.41% (0.29 to 0.52) | 83.48 (59.91 to 118.48) | 102.24 (72.46 to 147.51) | 0.54% (0.17 to 0.92) | 85.51 (60.96 to 120.64) | 96.92 (68.42 to 138.55) | 0.25% (-0.02 to 0.51) |
| New Zealand | 7217 (5166 to 10237) | 9321 (6654 to 13211) | 0.29% (0.2 to 0.38) | 211.18 (151.17 to 299.57) | 207.34 (148.01 to 293.86) | -0.04% (-0.08 to 0) | 214.06 (152.89 to 305.93) | 222.54 (158.34 to 317.15) | 0.14% (0.09 to 0.2) |
| Nicaragua | 2886 (2124 to 3988) | 3852 (2772 to 5436) | 0.34% (0.12 to 0.5) | 74.23 (54.64 to 102.59) | 59.17 (42.58 to 83.5) | -0.89% (-1.38 to -0.4) | 64.36 (48.06 to 87.91) | 57.56 (41.69 to 81.15) | -0.54% (-1.17 to 0.09) |
| Niger | 4146 (2943 to 5936) | 10925 (7842 to 15221) | 1.64% (1.47 to 1.79) | 51.67 (36.68 to 73.99) | 46.9 (33.66 to 65.34) | -0.01% (-0.44 to 0.42) | 54.71 (39.38 to 77.16) | 52.05 (37.7 to 70.37) | 0.16% (-0.19 to 0.52) |
| Nigeria | 37919 (27606 to 51245) | 100471 (72091 to 137414) | 1.65% (1.38 to 2.24) | 42.05 (30.61 to 56.82) | 46.77 (33.56 to 63.97) | 1.02% (0.54 to 1.5) | 44.57 (32.37 to 59.82) | 49.79 (36.05 to 67.32) | 0.98% (0.57 to 1.4) |
| Niue | 1 (1 to 1) | 1 (1 to 1) | -0.15% (-0.21 to -0.08) | 42.87 (30.17 to 58.51) | 50.72 (35.79 to 71.03) | 0.54% (-0.13 to 1.2) | 44.48 (31.32 to 60.71) | 51.12 (35.88 to 71.68) | 0.43% (-0.24 to 1.11) |
| North Macedonia | 3224 (2313 to 4482) | 2912 (2102 to 4131) | -0.1% (-0.26 to 0.05) | 159.99 (114.74 to 222.37) | 135.28 (97.63 to 191.92) | -0.77% (-0.9 to -0.64) | 158.3 (113.1 to 219.94) | 147.8 (105.45 to 209.7) | -0.45% (-0.6 to -0.3) |
| Northern Mariana Islands | 29 (20 to 39) | 26 (18 to 36) | -0.09% (-0.18 to 0.01) | 63 (44.27 to 86.04) | 61.33 (43.23 to 85.14) | -0.33% (-0.55 to -0.11) | 61.6 (43.98 to 83.93) | 62.28 (44.15 to 87.12) | -0.07% (-0.24 to 0.1) |
| Norway | 5789 (4047 to 8156) | 7176 (4938 to 10291) | 0.24% (0.17 to 0.3) | 136.31 (95.3 to 192.04) | 134.15 (92.31 to 192.39) | -0.18% (-0.24 to -0.12) | 126.72 (88.43 to 181.85) | 123.61 (83.31 to 182.11) | -0.2% (-0.27 to -0.12) |
| Oman | 1440 (1057 to 1946) | 3741 (2717 to 5199) | 1.6% (1.39 to 1.84) | 74.12 (54.42 to 100.15) | 81.61 (59.28 to 113.42) | 0.35% (0.27 to 0.43) | 73.32 (54.1 to 99.02) | 76.34 (56.24 to 105.64) | 0.02% (-0.06 to 0.11) |
| Pakistan | 42167 (31562 to 55000) | 101103 (74348 to 133417) | 1.4% (1.24 to 1.55) | 37.37 (27.97 to 48.74) | 45.12 (33.18 to 59.54) | 1.38% (0.88 to 1.88) | 38.1 (28.54 to 49.48) | 44.6 (32.92 to 58.72) | 1.21% (0.73 to 1.7) |
| Palau | 11 (8 to 16) | 15 (11 to 21) | 0.32% (0.24 to 0.39) | 74.16 (53.26 to 101.61) | 83.52 (60.14 to 114.65) | 0.26% (0.19 to 0.34) | 74.03 (53.54 to 100.63) | 83.42 (59.68 to 116.31) | 0.3% (0.26 to 0.34) |
| Palestine | 3661 (1959 to 7457) | 3609 (2623 to 4926) | -0.01% (-0.47 to 0.74) | 176.87 (94.64 to 360.23) | 72.82 (52.91 to 99.38) | -1.61% (-5.03 to 1.94) | 154.37 (85.39 to 303.31) | 66.87 (49.08 to 90.42) | -1.5% (-4.84 to 1.96) |
| Panama | 1608 (1184 to 2204) | 2558 (1873 to 3530) | 0.59% (0.51 to 0.67) | 67.33 (49.58 to 92.27) | 61.49 (45.02 to 84.84) | -0.43% (-0.52 to -0.33) | 63.5 (47.4 to 85.94) | 61.8 (45.24 to 85.58) | -0.21% (-0.31 to -0.12) |
| Papua New Guinea | 1604 (1188 to 2200) | 4357 (3076 to 6165) | 1.72% (1.28 to 2.02) | 39.24 (29.07 to 53.83) | 44.16 (31.18 to 62.48) | -0.12% (-0.97 to 0.75) | 39.2 (29.07 to 53.21) | 44.88 (32.08 to 64.33) | -0.05% (-0.91 to 0.81) |
| Paraguay | 3174 (2289 to 4423) | 5100 (3694 to 7064) | 0.61% (0.54 to 0.68) | 78.45 (56.58 to 109.32) | 73.59 (53.3 to 101.93) | -0.23% (-0.32 to -0.14) | 73.24 (53.47 to 100.92) | 71.24 (51.69 to 98.49) | -0.12% (-0.22 to -0.02) |
| Peru | 23951 (15219 to 41378) | 19977 (14704 to 27030) | -0.17% (-0.52 to 0.25) | 110.22 (70.04 to 190.42) | 58.76 (43.25 to 79.51) | -1.42% (-2.02 to -0.82) | 100.81 (65.15 to 171.21) | 58.39 (43.13 to 79.05) | -1.18% (-1.76 to -0.6) |
| Philippines | 37102 (27934 to 50722) | 50123 (37076 to 66483) | 0.35% (0.08 to 0.58) | 58.62 (44.14 to 80.14) | 44.7 (33.06 to 59.28) | -0.35% (-0.83 to 0.13) | 56.76 (42.83 to 77.1) | 44.19 (32.83 to 58.6) | -0.28% (-0.75 to 0.19) |
| Poland | 66147 (48110 to 91193) | 59800 (42827 to 84956) | -0.1% (-0.18 to -0.02) | 173.35 (126.08 to 238.99) | 155.59 (111.43 to 221.04) | -0.39% (-0.45 to -0.33) | 174.4 (126.29 to 240.27) | 163.81 (117.12 to 232.15) | -0.26% (-0.37 to -0.15) |
| Portugal | 10021 (7217 to 13768) | 7548 (5414 to 10724) | -0.25% (-0.3 to -0.19) | 98.86 (71.2 to 135.82) | 70.87 (50.83 to 100.68) | -1.55% (-1.67 to -1.42) | 101.48 (73.06 to 139.63) | 75.2 (54.1 to 107.15) | -1.41% (-1.54 to -1.29) |
| Puerto Rico | 2490 (1820 to 3358) | 2602 (1899 to 3583) | 0.04% (-0.06 to 0.14) | 68.92 (50.37 to 92.93) | 73.89 (53.92 to 101.76) | 0.58% (0.18 to 0.99) | 68.54 (50.08 to 92.37) | 74.25 (53.3 to 103.92) | 0.63% (0.21 to 1.06) |
| Qatar | 464 (340 to 633) | 3083 (2184 to 4354) | 5.64% (5.08 to 6.2) | 104.23 (76.31 to 142.27) | 107.61 (76.25 to 151.98) | 0.55% (0.33 to 0.77) | 89.55 (65.81 to 122.15) | 89.25 (64.91 to 124.07) | 0.15% (0.02 to 0.28) |
| Republic of Korea | 60809 (44229 to 83456) | 59544 (42622 to 83635) | -0.02% (-0.09 to 0.06) | 137.15 (99.76 to 188.23) | 111.51 (79.82 to 156.62) | -1.02% (-1.13 to -0.91) | 129.81 (94.75 to 177.74) | 116.44 (83.01 to 164.52) | -0.68% (-0.79 to -0.57) |
| Republic of Moldova | 6667 (4950 to 8925) | 4065 (2974 to 5624) | -0.39% (-0.43 to -0.35) | 149.95 (111.32 to 200.73) | 110.23 (80.64 to 152.5) | -1.42% (-1.7 to -1.15) | 149.43 (110.69 to 199.63) | 117.02 (85.75 to 161.7) | -1.24% (-1.55 to -0.93) |
| Romania | 44253 (31927 to 61725) | 27951 (19853 to 39777) | -0.37% (-0.4 to -0.34) | 189.15 (136.46 to 263.82) | 145.3 (103.2 to 206.77) | -1.12% (-1.23 to -1.01) | 192.13 (138.58 to 267.26) | 161.06 (114.28 to 228.79) | -0.79% (-0.92 to -0.66) |
| Russian Federation | 268347 (196402 to 362954) | 211831 (153193 to 294539) | -0.21% (-0.26 to -0.16) | 177.69 (130.05 to 240.33) | 144.38 (104.41 to 200.75) | -0.64% (-1.03 to -0.25) | 178.88 (130.87 to 240.76) | 149.3 (107.29 to 206.83) | -0.61% (-0.93 to -0.3) |
| Rwanda | 15167 (6674 to 32754) | 6801 (4813 to 9299) | -0.55% (-0.81 to 0.02) | 211.46 (93.05 to 456.64) | 53.6 (37.94 to 73.29) | -6% (-8.62 to -3.3) | 201.06 (94.54 to 426.26) | 55.54 (39.49 to 75.47) | -5.75% (-8.32 to -3.11) |
| Saint Kitts and Nevis | 27 (19 to 39) | 42 (30 to 61) | 0.55% (0.45 to 0.65) | 65.15 (46.93 to 93.94) | 70.17 (49.69 to 103.09) | 0.1% (-0.05 to 0.25) | 65.71 (47.39 to 95.07) | 71.6 (50.93 to 104.56) | 0.15% (0.01 to 0.3) |
| Saint Lucia | 74 (56 to 97) | 94 (70 to 126) | 0.27% (0.15 to 0.39) | 53.65 (40.93 to 70.6) | 53.77 (40.04 to 72.25) | -0.02% (-0.19 to 0.14) | 51.36 (39.21 to 67.02) | 56.46 (42.24 to 76.24) | 0.34% (0.18 to 0.5) |
| Saint Vincent and the Grenadines | 59 (45 to 78) | 65 (48 to 88) | 0.1% (0.02 to 0.18) | 53.72 (40.63 to 71.04) | 57.48 (42.83 to 77.97) | 0.13% (-0.08 to 0.34) | 50.82 (38.57 to 67.35) | 58.91 (43.97 to 80.19) | 0.41% (0.21 to 0.62) |
| Samoa | 68 (50 to 91) | 88 (63 to 123) | 0.3% (0.09 to 0.45) | 41.29 (30.78 to 55.89) | 41.57 (29.72 to 58.08) | 0.5% (-0.78 to 1.81) | 40.92 (30.99 to 54.59) | 41.39 (29.88 to 57.45) | 0.55% (-0.72 to 1.84) |
| San Marino | 24 (17 to 36) | 37 (26 to 54) | 0.52% (0.43 to 0.6) | 103.8 (72.52 to 151.63) | 112.14 (77.3 to 163.42) | 0.29% (0.23 to 0.36) | 106.11 (73.57 to 154.77) | 115.81 (78.11 to 173.52) | 0.33% (0.26 to 0.4) |
| Sao Tome and Principe | 60 (44 to 81) | 115 (84 to 153) | 0.91% (0.79 to 1.03) | 49.41 (36.44 to 66.37) | 55.79 (40.83 to 74.37) | 0.39% (0.33 to 0.45) | 50.63 (37.07 to 67.67) | 58.96 (43.1 to 78.54) | 0.54% (0.49 to 0.6) |
| Saudi Arabia | 21909 (15621 to 30461) | 66209 (45693 to 95052) | 2.02% (1.6 to 2.39) | 136.54 (97.36 to 189.84) | 185.29 (127.88 to 266.01) | 1.26% (1.11 to 1.41) | 130.38 (93.77 to 179.93) | 160.57 (111.82 to 230.97) | 0.82% (0.68 to 0.96) |
| Senegal | 3438 (2504 to 4738) | 6229 (4507 to 8487) | 0.81% (0.47 to 1.04) | 45.12 (32.86 to 62.17) | 41.16 (29.78 to 56.08) | -0.65% (-1.09 to -0.21) | 48.23 (34.9 to 65.73) | 44.92 (32.3 to 61.4) | -0.52% (-0.88 to -0.15) |
| Serbia | 12697 (9248 to 17592) | 11862 (8473 to 16856) | -0.07% (-0.14 to 0.01) | 135.11 (98.41 to 187.2) | 135.62 (96.87 to 192.72) | -0.87% (-1.94 to 0.2) | 140.16 (101.71 to 195.69) | 150.21 (107.05 to 214.45) | -0.66% (-1.77 to 0.46) |
| Seychelles | 37 (27 to 50) | 55 (39 to 74) | 0.48% (0.39 to 0.58) | 50.41 (36.41 to 68.65) | 53.45 (38.46 to 72.13) | 0.05% (-0.09 to 0.19) | 50.98 (36.99 to 69.27) | 52.45 (37.84 to 71.72) | -0.07% (-0.22 to 0.08) |
| Sierra Leone | 1499 (1093 to 2037) | 3415 (2457 to 4692) | 1.28% (1.16 to 1.39) | 41.05 (29.93 to 55.78) | 41.22 (29.66 to 56.63) | -6.02% (-8.69 to -3.27) | 43.2 (31.21 to 59.25) | 45.32 (32.59 to 62.84) | -5.46% (-8.01 to -2.85) |
| Singapore | 3437 (2497 to 4746) | 5120 (3647 to 7202) | 0.49% (0.4 to 0.59) | 112.78 (81.96 to 155.75) | 90.34 (64.36 to 127.07) | -0.91% (-1.01 to -0.82) | 105.66 (76.46 to 147) | 98.05 (69.8 to 139.2) | -0.43% (-0.52 to -0.34) |
| Slovakia | 9979 (7147 to 13882) | 9550 (6805 to 13680) | -0.04% (-0.1 to 0.01) | 188.88 (135.29 to 262.78) | 175.63 (125.16 to 251.6) | -0.32% (-0.39 to -0.25) | 188.03 (134.62 to 262.09) | 182.7 (130.11 to 260.08) | -0.16% (-0.27 to -0.06) |
| Slovenia | 4129 (2974 to 5819) | 3937 (2812 to 5605) | -0.05% (-0.11 to 0.02) | 209.48 (150.87 to 295.21) | 189.8 (135.58 to 270.19) | -0.03% (-0.16 to 0.11) | 207.37 (150.01 to 291.27) | 195.62 (139.65 to 282.76) | 0.16% (0.01 to 0.31) |
| Solomon Islands | 164 (117 to 229) | 375 (262 to 550) | 1.29% (1.12 to 1.44) | 48.17 (34.49 to 67.24) | 57.25 (39.97 to 83.92) | 0.58% (0.31 to 0.85) | 50.67 (36.36 to 70.78) | 58.9 (41.12 to 85.94) | 0.52% (0.26 to 0.78) |
| Somalia | 14670 (6057 to 33500) | 17514 (10838 to 29967) | 0.19% (-0.16 to 1.03) | 205.24 (84.74 to 468.69) | 86.1 (53.28 to 147.31) | 0.74% (-1.41 to 2.94) | 199.4 (86.33 to 435.37) | 86.54 (55.41 to 142.84) | 0.55% (-1.44 to 2.59) |
| South Africa | 21912 (15791 to 29412) | 25762 (18553 to 34418) | 0.18% (0.05 to 0.27) | 59.5 (42.88 to 79.86) | 46.34 (33.37 to 61.92) | -0.65% (-0.84 to -0.45) | 59.42 (42.72 to 79.99) | 45.96 (33.2 to 61.38) | -0.7% (-0.89 to -0.51) |
| South Sudan | 3196 (2274 to 4373) | 6880 (4602 to 10509) | 1.15% (0.68 to 2.3) | 54.54 (38.81 to 74.63) | 74.12 (49.57 to 113.21) | 2.09% (-0.39 to 4.64) | 58.45 (41.62 to 79.83) | 75.28 (51.56 to 113.92) | 1.82% (-0.45 to 4.14) |
| Spain | 39668 (28756 to 54365) | 42966 (30151 to 62655) | 0.08% (-0.03 to 0.19) | 102.29 (74.15 to 140.18) | 93.36 (65.52 to 136.14) | -0.52% (-0.59 to -0.45) | 105.07 (76.37 to 145.26) | 104.55 (71.38 to 154.66) | -0.2% (-0.27 to -0.14) |
| Sri Lanka | 36539 (17955 to 77488) | 13347 (9845 to 18394) | -0.63% (-0.81 to -0.27) | 212.17 (104.26 to 449.94) | 61.07 (45.05 to 84.17) | -3.83% (-5.66 to -1.96) | 198.51 (98.46 to 416.39) | 61.42 (45.48 to 83.99) | -3.59% (-5.43 to -1.71) |
| Sudan | 32235 (15075 to 67523) | 23747 (17290 to 32845) | -0.26% (-0.66 to 0.54) | 159.59 (74.63 to 334.29) | 58.19 (42.37 to 80.49) | -2% (-3.2 to -0.79) | 150.12 (72.41 to 305.87) | 56.19 (41.14 to 77.26) | -1.98% (-3.13 to -0.81) |
| Suriname | 187 (142 to 244) | 302 (228 to 401) | 0.62% (0.53 to 0.72) | 48.38 (36.7 to 63.08) | 52.48 (39.57 to 69.72) | 0.29% (0.25 to 0.34) | 45.92 (34.9 to 59.86) | 53.44 (40.34 to 71.03) | 0.53% (0.48 to 0.58) |
| Sweden | 10606 (7368 to 15119) | 13234 (9070 to 18950) | 0.25% (0.18 to 0.31) | 123.49 (85.78 to 176.03) | 129.46 (88.72 to 185.37) | 0.12% (0.06 to 0.17) | 118.53 (82.28 to 172.88) | 120.15 (81.21 to 177.89) | 0% (-0.11 to 0.1) |
| Switzerland | 11383 (8079 to 16297) | 11866 (8212 to 17303) | 0.04% (-0.02 to 0.11) | 165.8 (117.68 to 237.38) | 135.22 (93.58 to 197.18) | -1.09% (-1.23 to -0.96) | 163.98 (116.18 to 232.44) | 139.25 (93.95 to 206.12) | -0.98% (-1.17 to -0.8) |
| Syrian Arab Republic | 7518 (5483 to 10360) | 21244 (12083 to 41301) | 1.83% (0.74 to 4.08) | 58.3 (42.52 to 80.34) | 146.6 (83.38 to 285.01) | 10.06% (6.49 to 13.74) | 52.55 (38.64 to 71.95) | 169.93 (89.08 to 344.22) | 10.69% (7.07 to 14.44) |
| Taiwan (Province of China) | 9867 (7146 to 13623) | 7812 (5669 to 10691) | -0.21% (-0.27 to -0.14) | 48.37 (35.03 to 66.78) | 33.07 (24 to 45.26) | -1.84% (-2.16 to -1.51) | 47.36 (34.5 to 65.7) | 30.16 (22.15 to 41.47) | -2.07% (-2.41 to -1.74) |
| Tajikistan | 5385 (3923 to 7467) | 7883 (5702 to 10887) | 0.46% (0.39 to 0.54) | 100.17 (72.98 to 138.9) | 83.05 (60.07 to 114.7) | -3.59% (-5.48 to -1.66) | 94.53 (69.23 to 130.31) | 77.48 (56.19 to 107.18) | -3.53% (-5.39 to -1.62) |
| Thailand | 30851 (23320 to 40424) | 35524 (26486 to 48186) | 0.15% (0.03 to 0.28) | 54.24 (41 to 71.07) | 50.67 (37.78 to 68.73) | -0.27% (-0.49 to -0.05) | 52.43 (39.65 to 68.32) | 50.65 (37.5 to 68.33) | -0.15% (-0.37 to 0.07) |
| Timor-Leste | 1267 (582 to 2797) | 527 (389 to 704) | -0.58% (-0.82 to -0.08) | 161.83 (74.39 to 357.23) | 39.5 (29.15 to 52.75) | -7.57% (-9.89 to -5.19) | 143.63 (68.41 to 306.81) | 39.01 (29.1 to 51.84) | -7.12% (-9.37 to -4.81) |
| Togo | 1614 (1162 to 2244) | 3621 (2587 to 4971) | 1.24% (1.14 to 1.35) | 44.06 (31.73 to 61.26) | 45.71 (32.66 to 62.75) | -0.02% (-0.51 to 0.47) | 47.34 (33.82 to 65.46) | 48.65 (34.61 to 67.2) | -0.01% (-0.44 to 0.41) |
| Tokelau | 1 (0 to 1) | 1 (0 to 1) | -0.01% (-0.08 to 0.07) | 37.88 (26.94 to 51.48) | 44.92 (31.66 to 62.47) | 0.6% (0.52 to 0.68) | 40.21 (28.48 to 54.81) | 46.6 (32.88 to 64.75) | 0.49% (0.39 to 0.59) |
| Tonga | 34 (25 to 46) | 38 (27 to 53) | 0.12% (0.01 to 0.21) | 34.74 (25.73 to 47.33) | 36.7 (26.32 to 51.83) | 0.26% (-0.06 to 0.59) | 34.7 (25.83 to 46.51) | 36.41 (26.12 to 51.21) | 0.23% (-0.1 to 0.57) |
| Trinidad and Tobago | 719 (540 to 980) | 669 (505 to 881) | -0.07% (-0.26 to 0.07) | 59.79 (44.89 to 81.43) | 48.22 (36.4 to 63.52) | -0.4% (-0.54 to -0.26) | 57.63 (43.22 to 78.71) | 51.61 (38.51 to 68.39) | -0.02% (-0.17 to 0.14) |
| Tunisia | 4987 (3675 to 6759) | 6892 (4996 to 9533) | 0.38% (0.29 to 0.48) | 59.09 (43.54 to 80.09) | 59.56 (43.18 to 82.38) | 0.15% (0.07 to 0.22) | 56.18 (41.35 to 76.34) | 60.6 (44.05 to 83.18) | 0.37% (0.29 to 0.46) |
| Turkey | 36376 (26989 to 49668) | 51123 (37360 to 70774) | 0.41% (0.2 to 0.62) | 60.86 (45.15 to 83.09) | 62.84 (45.92 to 86.99) | -0.51% (-1.29 to 0.29) | 57.12 (42.47 to 77.9) | 63 (45.95 to 87.51) | -0.25% (-1.04 to 0.54) |
| Turkmenistan | 3170 (2359 to 4340) | 4315 (3147 to 5941) | 0.36% (0.3 to 0.44) | 85.56 (63.67 to 117.14) | 84.88 (61.92 to 116.87) | -0.45% (-0.85 to -0.04) | 78.15 (58.42 to 106.05) | 82.47 (60.22 to 113.98) | -0.25% (-0.71 to 0.22) |
| Tuvalu | 4 (3 to 5) | 5 (4 to 8) | 0.5% (0.4 to 0.62) | 39.01 (27.74 to 53.19) | 46.41 (32.63 to 64.77) | 0.55% (0.44 to 0.65) | 40.04 (28.47 to 54.43) | 46.33 (32.9 to 64.34) | 0.43% (0.35 to 0.52) |
| Uganda | 12022 (7939 to 18966) | 20917 (15247 to 28547) | 0.74% (0.14 to 1.28) | 69.43 (45.85 to 109.53) | 50.87 (37.08 to 69.43) | -1.08% (-1.93 to -0.22) | 69.01 (46.82 to 105.74) | 52.18 (37.92 to 71) | -0.99% (-1.74 to -0.23) |
| Ukraine | 96170 (68085 to 135139) | 65272 (46596 to 90916) | -0.32% (-0.35 to -0.29) | 182.61 (129.28 to 256.61) | 148.2 (105.8 to 206.43) | -0.91% (-1.11 to -0.71) | 187.74 (133.42 to 264.67) | 158.99 (114.24 to 220.48) | -0.79% (-1.04 to -0.55) |
| United Arab Emirates | 1450 (1065 to 1964) | 7037 (5182 to 9534) | 3.85% (3.51 to 4.24) | 77.43 (56.89 to 104.9) | 76.14 (56.07 to 103.16) | 0.04% (-0.12 to 0.21) | 70.78 (52.16 to 95.06) | 70.15 (51.26 to 95.57) | -0.15% (-0.21 to -0.09) |
| United Kingdom | 57352 (41569 to 80077) | 68156 (47784 to 98136) | 0.19% (0.1 to 0.26) | 99.79 (72.33 to 139.33) | 101.39 (71.09 to 145.99) | 0.04% (-0.04 to 0.13) | 106.13 (76.04 to 148.56) | 105.95 (73.99 to 154.08) | -0.04% (-0.13 to 0.06) |
| United Republic of Tanzania | 12974 (9418 to 17643) | 28444 (20579 to 39256) | 1.19% (1.11 to 1.29) | 50.09 (36.36 to 68.12) | 50.13 (36.27 to 69.19) | 0.02% (-0.1 to 0.14) | 51.78 (37.81 to 70.69) | 51.6 (37.48 to 70.59) | 0% (-0.1 to 0.1) |
| United States of America | 306688 (219361 to 430828) | 388647 (283697 to 533349) | 0.27% (0.17 to 0.37) | 120.93 (86.5 to 169.88) | 118.5 (86.5 to 162.62) | -0.31% (-0.57 to -0.05) | 118.91 (84.9 to 168.79) | 108.04 (78.65 to 150.59) | -0.74% (-1.05 to -0.44) |
| United States Virgin Islands | 63 (46 to 86) | 64 (46 to 88) | 0.01% (-0.07 to 0.09) | 59.59 (43.61 to 80.9) | 61.34 (44.69 to 84.76) | -0.01% (-0.13 to 0.12) | 60 (43.96 to 81.32) | 63.64 (46.24 to 88.61) | 0.08% (-0.06 to 0.23) |
| Uruguay | 3838 (2669 to 5668) | 3696 (2654 to 5239) | -0.04% (-0.12 to 0.05) | 122.27 (85 to 180.56) | 107.56 (77.24 to 152.46) | -0.62% (-0.71 to -0.52) | 124.52 (86.58 to 183.86) | 112.34 (80.25 to 159.79) | -0.53% (-0.64 to -0.43) |
| Uzbekistan | 20811 (15164 to 28210) | 30528 (22287 to 41695) | 0.47% (0.39 to 0.53) | 99.35 (72.39 to 134.67) | 90.65 (66.18 to 123.81) | -0.49% (-0.62 to -0.37) | 91.74 (67.29 to 123.7) | 86.07 (62.99 to 117.95) | -0.4% (-0.5 to -0.3) |
| Vanuatu | 51 (37 to 69) | 110 (80 to 150) | 1.17% (1.04 to 1.31) | 33.4 (24.65 to 45.53) | 37.21 (27.04 to 51.04) | -0.26% (-1.42 to 0.92) | 33.06 (24.37 to 44.55) | 36.58 (26.72 to 50.11) | -0.29% (-1.47 to 0.9) |
| Venezuela (Bolivarian Republic of) | 15340 (11304 to 20919) | 19188 (13967 to 26560) | 0.25% (0.18 to 0.32) | 81.46 (60.03 to 111.09) | 68.36 (49.76 to 94.62) | -0.9% (-1.73 to -0.06) | 75 (56.02 to 101.89) | 70.67 (51.2 to 98.27) | -0.52% (-1.36 to 0.32) |
| Viet Nam | 29657 (21965 to 40052) | 49653 (36796 to 66590) | 0.67% (0.51 to 0.84) | 43.65 (32.33 to 58.95) | 51.52 (38.18 to 69.1) | 0.49% (0.36 to 0.63) | 44.15 (32.92 to 59.52) | 51.65 (38.23 to 69.12) | 0.42% (0.28 to 0.56) |
| Yemen | 7839 (5790 to 10650) | 79881 (36953 to 179043) | 9.19% (3.56 to 23.21) | 57.1 (42.18 to 77.57) | 253.57 (117.3 to 568.34) | 4.86% (3.09 to 6.65) | 54.97 (40.64 to 74.26) | 223.74 (104.73 to 482.89) | 4.79% (3 to 6.61) |
| Zambia | 3820 (2819 to 5179) | 8709 (6301 to 12002) | 1.28% (1.04 to 1.47) | 48.09 (35.49 to 65.2) | 47.75 (34.55 to 65.81) | 0.12% (0 to 0.24) | 50.92 (37.76 to 68.65) | 50.54 (36.6 to 68.53) | 0.08% (-0.01 to 0.17) |
| Zimbabwe | 4133 (3041 to 5552) | 6817 (5101 to 9289) | 0.65% (0.47 to 1.05) | 39.97 (29.42 to 53.71) | 45.41 (33.99 to 61.88) | 0.06% (-0.22 to 0.34) | 41.94 (31.24 to 56.06) | 46.79 (35.02 to 62.87) | -0.01% (-0.26 to 0.25) |

CR, crude rate; ASR, age-standardized rate; EAPC, estimated annual percentage change; UI, uncertainty interval; CI, confidence interval.

Table S4. YLDs of traumatic shoulder dislocation in 1990 and 2019 for both sexes in 204 countries and territories, with EAPC from 1990 and 2019.

| **Location** | **Number** | | | **CR, per 100k** | | | **ASR, per 100k** | | |
| --- | --- | --- | --- | --- | --- | --- | --- | --- | --- |
|  | **Number in 1990 (95% UI)** | **Number in 2019 (95% UI)** | **Number change (95% UI)** | **CR in 1990 (95% UI)** | **CR in 2019 (95% UI)** | **EAPC of CR (95% CI)** | **ASR in 1990 (95% UI)** | **ASR in 2019 (95% UI)** | **EAPC of ASR (95% CI)** |
| Afghanistan | 120 (25 to 304) | 764 (173 to 2033) | 5.38% (3.76 to 6.97) | 1.05 (0.22 to 2.66) | 2 (0.45 to 5.31) | 1.81% (-0.07 to 3.73) | 0.97 (0.21 to 2.38) | 2.55 (0.55 to 7.01) | 2.15% (0.34 to 4) |
| Albania | 52 (11 to 107) | 35 (9 to 72) | -0.32% (-0.39 to -0.2) | 1.56 (0.34 to 3.23) | 1.3 (0.31 to 2.65) | -1.29% (-2 to -0.58) | 1.48 (0.33 to 3.06) | 1.41 (0.34 to 2.92) | -0.82% (-1.52 to -0.12) |
| Algeria | 151 (33 to 303) | 231 (53 to 472) | 0.53% (0.44 to 0.64) | 0.6 (0.13 to 1.2) | 0.55 (0.13 to 1.13) | -1.86% (-2.67 to -1.04) | 0.56 (0.13 to 1.12) | 0.54 (0.13 to 1.11) | -1.63% (-2.4 to -0.85) |
| American Samoa | 0 (0 to 0) | 0 (0 to 1) | 0.16% (0.08 to 0.28) | 0.45 (0.11 to 0.92) | 0.46 (0.12 to 0.94) | 0.24% (-0.73 to 1.21) | 0.47 (0.13 to 0.95) | 0.47 (0.13 to 0.95) | 0.24% (-0.7 to 1.19) |
| Andorra | 1 (0 to 1) | 1 (0 to 2) | 0.61% (0.5 to 0.9) | 0.99 (0.24 to 2.05) | 1.04 (0.28 to 2.13) | 0.18% (0.14 to 0.21) | 1.03 (0.25 to 2.17) | 1.09 (0.27 to 2.31) | 0.14% (0.09 to 0.19) |
| Angola | 155 (34 to 430) | 95 (22 to 188) | -0.39% (-0.73 to 0.37) | 1.5 (0.33 to 4.17) | 0.31 (0.07 to 0.62) | -6.74% (-9.25 to -4.16) | 1.33 (0.29 to 3.62) | 0.31 (0.08 to 0.62) | -6.26% (-8.65 to -3.8) |
| Antigua and Barbuda | 0 (0 to 1) | 1 (0 to 1) | 0.51% (0.4 to 0.63) | 0.55 (0.13 to 1.16) | 0.57 (0.13 to 1.18) | -0.12% (-0.37 to 0.13) | 0.53 (0.12 to 1.12) | 0.59 (0.14 to 1.22) | 0.09% (-0.19 to 0.37) |
| Argentina | 318 (73 to 646) | 412 (97 to 845) | 0.3% (0.24 to 0.36) | 0.96 (0.22 to 1.95) | 0.91 (0.22 to 1.87) | -0.28% (-0.35 to -0.21) | 0.96 (0.22 to 1.95) | 0.93 (0.22 to 1.92) | -0.2% (-0.27 to -0.14) |
| Armenia | 40 (9 to 79) | 22 (5 to 44) | -0.45% (-0.49 to -0.41) | 1.16 (0.26 to 2.32) | 0.72 (0.17 to 1.46) | -1.74% (-2.02 to -1.45) | 1.13 (0.26 to 2.25) | 0.78 (0.18 to 1.6) | -1.33% (-1.65 to -1.02) |
| Australia | 277 (66 to 567) | 403 (99 to 826) | 0.46% (0.37 to 0.65) | 1.64 (0.39 to 3.36) | 1.64 (0.4 to 3.36) | 0% (-0.09 to 0.09) | 1.69 (0.4 to 3.51) | 1.74 (0.43 to 3.62) | 0.1% (0.03 to 0.17) |
| Austria | 94 (23 to 191) | 95 (25 to 195) | 0.02% (-0.04 to 0.11) | 1.2 (0.3 to 2.46) | 1.07 (0.28 to 2.19) | -0.52% (-0.56 to -0.48) | 1.2 (0.29 to 2.49) | 1.09 (0.26 to 2.3) | -0.44% (-0.48 to -0.4) |
| Azerbaijan | 60 (14 to 121) | 75 (17 to 153) | 0.25% (0.18 to 0.33) | 0.82 (0.19 to 1.65) | 0.73 (0.17 to 1.49) | -1.46% (-2.39 to -0.53) | 0.77 (0.18 to 1.54) | 0.74 (0.17 to 1.5) | -1.18% (-2.13 to -0.22) |
| Bahamas | 1 (0 to 2) | 2 (0 to 4) | 0.56% (0.45 to 0.7) | 0.45 (0.1 to 0.91) | 0.48 (0.12 to 0.96) | 0.12% (-0.06 to 0.31) | 0.43 (0.1 to 0.86) | 0.49 (0.12 to 0.98) | 0.34% (0.16 to 0.53) |
| Bahrain | 3 (1 to 5) | 8 (2 to 16) | 1.88% (1.66 to 2.15) | 0.53 (0.12 to 1.05) | 0.54 (0.12 to 1.1) | 0.27% (0.15 to 0.4) | 0.48 (0.11 to 0.96) | 0.56 (0.13 to 1.15) | 0.6% (0.46 to 0.75) |
| Bangladesh | 420 (95 to 839) | 706 (168 to 1441) | 0.68% (0.53 to 0.83) | 0.39 (0.09 to 0.77) | 0.44 (0.11 to 0.91) | -0.65% (-2.11 to 0.83) | 0.38 (0.09 to 0.76) | 0.43 (0.1 to 0.88) | -0.67% (-2.09 to 0.77) |
| Barbados | 1 (0 to 2) | 1 (0 to 3) | 0.2% (0.13 to 0.32) | 0.43 (0.1 to 0.85) | 0.44 (0.11 to 0.87) | -0.14% (-0.32 to 0.04) | 0.42 (0.1 to 0.84) | 0.48 (0.11 to 0.97) | 0.23% (0.06 to 0.41) |
| Belarus | 151 (36 to 302) | 132 (32 to 268) | -0.12% (-0.17 to -0.05) | 1.44 (0.35 to 2.88) | 1.39 (0.33 to 2.82) | 0.21% (-0.11 to 0.52) | 1.46 (0.34 to 2.92) | 1.44 (0.33 to 2.96) | 0.2% (-0.04 to 0.44) |
| Belgium | 110 (28 to 228) | 148 (40 to 304) | 0.35% (0.26 to 0.51) | 1.1 (0.28 to 2.28) | 1.3 (0.35 to 2.66) | 1.09% (0.74 to 1.44) | 1.14 (0.28 to 2.4) | 1.27 (0.31 to 2.7) | 1.07% (0.64 to 1.51) |
| Belize | 1 (0 to 2) | 2 (1 to 5) | 0.97% (0.42 to 1.41) | 0.64 (0.14 to 1.31) | 0.58 (0.13 to 1.16) | -0.53% (-0.96 to -0.1) | 0.57 (0.13 to 1.15) | 0.55 (0.13 to 1.11) | -0.37% (-0.79 to 0.05) |
| Benin | 19 (5 to 37) | 49 (13 to 99) | 1.66% (1.54 to 1.78) | 0.38 (0.1 to 0.77) | 0.39 (0.1 to 0.79) | 0.04% (-0.02 to 0.11) | 0.43 (0.12 to 0.88) | 0.44 (0.13 to 0.9) | 0.11% (0.07 to 0.15) |
| Bermuda | 0 (0 to 1) | 0 (0 to 1) | 0.18% (0.05 to 0.39) | 0.53 (0.14 to 1.07) | 0.57 (0.18 to 1.14) | 0.27% (0.16 to 0.39) | 0.53 (0.14 to 1.07) | 0.6 (0.17 to 1.22) | 0.43% (0.32 to 0.54) |
| Bhutan | 3 (1 to 6) | 4 (1 to 9) | 0.58% (0.45 to 0.77) | 0.46 (0.11 to 0.93) | 0.6 (0.15 to 1.18) | 0.72% (0.32 to 1.13) | 0.47 (0.12 to 0.93) | 0.59 (0.15 to 1.17) | 0.63% (0.25 to 1.02) |
| Bolivia (Plurinational State of) | 35 (8 to 71) | 62 (14 to 124) | 0.76% (0.61 to 0.93) | 0.55 (0.12 to 1.11) | 0.52 (0.12 to 1.03) | -0.39% (-0.47 to -0.32) | 0.52 (0.12 to 1.06) | 0.51 (0.12 to 1.01) | -0.29% (-0.38 to -0.21) |
| Bosnia and Herzegovina | 73 (17 to 151) | 41 (10 to 84) | -0.44% (-0.56 to -0.34) | 1.61 (0.38 to 3.33) | 1.24 (0.3 to 2.54) | -3.35% (-5.21 to -1.45) | 1.6 (0.38 to 3.28) | 1.41 (0.33 to 2.93) | -2.98% (-4.9 to -1.02) |
| Botswana | 6 (1 to 11) | 11 (3 to 21) | 0.87% (0.68 to 1.1) | 0.43 (0.11 to 0.88) | 0.45 (0.12 to 0.92) | 0.2% (0.12 to 0.27) | 0.43 (0.12 to 0.87) | 0.45 (0.13 to 0.91) | 0.13% (0.05 to 0.21) |
| Brazil | 1301 (323 to 2645) | 1633 (452 to 3357) | 0.26% (0.19 to 0.4) | 0.87 (0.22 to 1.78) | 0.75 (0.21 to 1.55) | -0.47% (-0.52 to -0.42) | 0.86 (0.23 to 1.75) | 0.75 (0.2 to 1.51) | -0.42% (-0.48 to -0.37) |
| Brunei Darussalam | 3 (1 to 6) | 4 (1 to 8) | 0.51% (0.42 to 0.61) | 1.06 (0.24 to 2.17) | 0.94 (0.22 to 1.92) | -0.43% (-0.52 to -0.34) | 1 (0.24 to 2.01) | 0.93 (0.22 to 1.88) | -0.32% (-0.41 to -0.24) |
| Bulgaria | 140 (33 to 285) | 90 (22 to 181) | -0.36% (-0.42 to -0.3) | 1.62 (0.38 to 3.29) | 1.3 (0.31 to 2.61) | -0.83% (-0.88 to -0.79) | 1.72 (0.4 to 3.5) | 1.52 (0.36 to 3.18) | -0.49% (-0.57 to -0.42) |
| Burkina Faso | 35 (9 to 70) | 123 (30 to 256) | 2.55% (1.87 to 3.96) | 0.36 (0.1 to 0.73) | 0.54 (0.13 to 1.13) | 0.7% (0.39 to 1.02) | 0.41 (0.12 to 0.85) | 0.6 (0.16 to 1.23) | 0.73% (0.46 to 1) |
| Burundi | 23 (5 to 45) | 51 (12 to 103) | 1.24% (1.09 to 1.48) | 0.41 (0.09 to 0.81) | 0.43 (0.1 to 0.86) | -7.38% (-12.11 to -2.41) | 0.42 (0.1 to 0.83) | 0.42 (0.11 to 0.83) | -7.16% (-11.67 to -2.41) |
| Cabo Verde | 1 (0 to 3) | 3 (1 to 5) | 0.89% (0.74 to 1.07) | 0.39 (0.1 to 0.77) | 0.46 (0.12 to 0.92) | 0.7% (0.62 to 0.79) | 0.39 (0.11 to 0.79) | 0.46 (0.13 to 0.93) | 0.66% (0.6 to 0.71) |
| Cambodia | 56 (13 to 116) | 81 (19 to 166) | 0.44% (-0.02 to 0.9) | 0.54 (0.12 to 1.12) | 0.49 (0.12 to 1) | -0.96% (-1.62 to -0.3) | 0.52 (0.13 to 1.07) | 0.49 (0.12 to 1) | -0.77% (-1.37 to -0.16) |
| Cameroon | 42 (11 to 87) | 152 (39 to 314) | 2.58% (2.12 to 3.53) | 0.41 (0.11 to 0.84) | 0.52 (0.13 to 1.08) | 1.25% (0.78 to 1.73) | 0.46 (0.13 to 0.97) | 0.57 (0.16 to 1.18) | 1.08% (0.71 to 1.46) |
| Canada | 239 (61 to 476) | 345 (99 to 679) | 0.44% (0.32 to 0.71) | 0.88 (0.22 to 1.75) | 0.94 (0.27 to 1.86) | 0.3% (0.24 to 0.36) | 0.85 (0.21 to 1.7) | 0.84 (0.21 to 1.69) | -0.06% (-0.11 to -0.02) |
| Central African Republic | 8 (2 to 15) | 30 (7 to 71) | 2.81% (1.5 to 5.9) | 0.29 (0.07 to 0.56) | 0.57 (0.13 to 1.34) | 5.71% (3.4 to 8.06) | 0.29 (0.07 to 0.56) | 0.53 (0.13 to 1.24) | 5.3% (3.1 to 7.54) |
| Chad | 48 (10 to 120) | 73 (19 to 150) | 0.53% (-0.02 to 1.35) | 0.79 (0.17 to 2) | 0.44 (0.11 to 0.92) | -1.39% (-2.65 to -0.1) | 0.79 (0.18 to 1.96) | 0.5 (0.13 to 1.01) | -1.02% (-2.15 to 0.12) |
| Chile | 123 (29 to 251) | 171 (41 to 350) | 0.39% (0.31 to 0.51) | 0.93 (0.22 to 1.89) | 0.94 (0.23 to 1.93) | -0.05% (-0.27 to 0.18) | 0.9 (0.22 to 1.83) | 0.98 (0.24 to 2) | 0.26% (0.03 to 0.5) |
| China | 5098 (1366 to 10427) | 8395 (2548 to 16997) | 0.65% (0.46 to 1.03) | 0.43 (0.12 to 0.88) | 0.59 (0.18 to 1.19) | 0.58% (0.17 to 0.99) | 0.44 (0.12 to 0.89) | 0.54 (0.16 to 1.09) | 0.21% (-0.18 to 0.61) |
| Colombia | 307 (69 to 620) | 263 (64 to 535) | -0.14% (-0.34 to 0.01) | 0.94 (0.21 to 1.9) | 0.55 (0.13 to 1.12) | -2.31% (-2.64 to -1.99) | 0.88 (0.21 to 1.76) | 0.57 (0.14 to 1.18) | -1.94% (-2.25 to -1.63) |
| Comoros | 3 (1 to 5) | 4 (1 to 8) | 0.61% (0.52 to 0.73) | 0.54 (0.14 to 1.1) | 0.56 (0.15 to 1.16) | -0.17% (-0.62 to 0.29) | 0.57 (0.15 to 1.19) | 0.58 (0.16 to 1.17) | -0.29% (-0.69 to 0.11) |
| Congo | 7 (2 to 15) | 15 (4 to 31) | 1.08% (1 to 1.18) | 0.3 (0.07 to 0.6) | 0.29 (0.07 to 0.58) | -2.98% (-6.32 to 0.48) | 0.3 (0.08 to 0.6) | 0.29 (0.07 to 0.58) | -2.83% (-6.02 to 0.47) |
| Cook Islands | 0 (0 to 0) | 0 (0 to 0) | -0.01% (-0.12 to 0.12) | 0.48 (0.13 to 0.98) | 0.5 (0.14 to 1.02) | -0.12% (-1.02 to 0.78) | 0.5 (0.15 to 1.01) | 0.51 (0.14 to 1.02) | -0.21% (-1.19 to 0.77) |
| Costa Rica | 20 (5 to 42) | 28 (7 to 58) | 0.43% (0.35 to 0.56) | 0.66 (0.15 to 1.37) | 0.6 (0.15 to 1.24) | -0.29% (-0.39 to -0.18) | 0.63 (0.15 to 1.28) | 0.63 (0.15 to 1.3) | 0.06% (-0.05 to 0.17) |
| Côte d'Ivoire | 49 (13 to 98) | 106 (28 to 215) | 1.18% (1.1 to 1.28) | 0.4 (0.1 to 0.8) | 0.4 (0.11 to 0.82) | 0.12% (-0.56 to 0.79) | 0.46 (0.13 to 0.94) | 0.46 (0.13 to 0.94) | 0.02% (-0.54 to 0.59) |
| Croatia | 82 (20 to 162) | 67 (19 to 133) | -0.19% (-0.31 to 0) | 1.67 (0.41 to 3.32) | 1.57 (0.44 to 3.13) | -0.96% (-1.46 to -0.46) | 1.69 (0.41 to 3.36) | 1.55 (0.39 to 3.2) | -1.03% (-1.49 to -0.57) |
| Cuba | 69 (17 to 135) | 88 (24 to 171) | 0.28% (0.14 to 0.53) | 0.64 (0.16 to 1.25) | 0.78 (0.21 to 1.51) | 0.72% (0.64 to 0.8) | 0.62 (0.16 to 1.22) | 0.76 (0.19 to 1.5) | 0.7% (0.64 to 0.76) |
| Cyprus | 7 (2 to 15) | 13 (3 to 26) | 0.75% (0.62 to 0.92) | 0.94 (0.23 to 1.95) | 0.97 (0.25 to 1.99) | 0.1% (0.03 to 0.18) | 0.96 (0.23 to 1.98) | 1.04 (0.25 to 2.2) | 0.3% (0.25 to 0.35) |
| Czechia | 184 (46 to 369) | 157 (40 to 318) | -0.14% (-0.18 to -0.1) | 1.78 (0.45 to 3.58) | 1.48 (0.38 to 2.99) | -0.63% (-0.71 to -0.54) | 1.76 (0.43 to 3.56) | 1.59 (0.39 to 3.29) | -0.2% (-0.27 to -0.12) |
| Democratic People's Republic of Korea | 57 (14 to 118) | 61 (16 to 124) | 0.06% (-0.04 to 0.22) | 0.27 (0.07 to 0.56) | 0.23 (0.06 to 0.47) | -0.38% (-0.6 to -0.15) | 0.27 (0.07 to 0.56) | 0.23 (0.06 to 0.46) | -0.49% (-0.71 to -0.28) |
| Democratic Republic of the Congo | 123 (29 to 243) | 339 (77 to 684) | 1.75% (1.41 to 2.41) | 0.32 (0.07 to 0.63) | 0.39 (0.09 to 0.78) | -0.98% (-3.37 to 1.48) | 0.31 (0.08 to 0.6) | 0.37 (0.09 to 0.75) | -0.95% (-3.21 to 1.37) |
| Denmark | 56 (15 to 113) | 55 (14 to 113) | -0.03% (-0.09 to 0.03) | 1.09 (0.29 to 2.2) | 0.94 (0.24 to 1.94) | -0.72% (-0.83 to -0.61) | 1.06 (0.26 to 2.2) | 1.01 (0.24 to 2.17) | -0.26% (-0.37 to -0.16) |
| Djibouti | 5 (1 to 12) | 6 (2 to 12) | 0.3% (-0.3 to 1.11) | 0.96 (0.2 to 2.43) | 0.5 (0.13 to 1.02) | -2.55% (-3.92 to -1.15) | 0.94 (0.22 to 2.27) | 0.53 (0.14 to 1.08) | -2.34% (-3.6 to -1.06) |
| Dominica | 0 (0 to 1) | 0 (0 to 1) | -0.04% (-0.09 to 0.03) | 0.45 (0.1 to 0.89) | 0.46 (0.11 to 0.92) | 0.55% (0.07 to 1.03) | 0.43 (0.1 to 0.86) | 0.48 (0.11 to 0.95) | 0.72% (0.29 to 1.14) |
| Dominican Republic | 34 (8 to 69) | 62 (14 to 122) | 0.84% (0.72 to 0.97) | 0.47 (0.1 to 0.96) | 0.57 (0.13 to 1.12) | 0.7% (0.52 to 0.87) | 0.43 (0.1 to 0.88) | 0.56 (0.13 to 1.09) | 0.86% (0.68 to 1.04) |
| Ecuador | 59 (13 to 115) | 108 (25 to 215) | 0.85% (0.72 to 1) | 0.58 (0.13 to 1.15) | 0.61 (0.14 to 1.22) | 0.08% (-0.08 to 0.23) | 0.56 (0.13 to 1.1) | 0.6 (0.14 to 1.2) | 0.18% (0.03 to 0.34) |
| Egypt | 232 (51 to 466) | 476 (107 to 972) | 1.05% (0.9 to 1.26) | 0.42 (0.09 to 0.84) | 0.48 (0.11 to 0.98) | 0.44% (0.2 to 0.69) | 0.39 (0.09 to 0.78) | 0.46 (0.11 to 0.93) | 0.53% (0.28 to 0.78) |
| El Salvador | 82 (18 to 203) | 38 (9 to 76) | -0.54% (-0.77 to -0.2) | 1.56 (0.35 to 3.86) | 0.61 (0.15 to 1.21) | -1.48% (-2.37 to -0.57) | 1.39 (0.32 to 3.39) | 0.6 (0.15 to 1.21) | -1.18% (-2.05 to -0.3) |
| Equatorial Guinea | 1 (0 to 3) | 4 (1 to 9) | 2.35% (2.07 to 2.66) | 0.31 (0.07 to 0.61) | 0.31 (0.07 to 0.62) | 0.07% (-0.14 to 0.27) | 0.3 (0.08 to 0.59) | 0.31 (0.08 to 0.61) | 0.06% (-0.13 to 0.24) |
| Eritrea | 312 (46 to 1017) | 34 (9 to 68) | -0.89% (-0.96 to -0.62) | 10.39 (1.53 to 33.89) | 0.5 (0.13 to 1.02) | -4.79% (-8.12 to -1.34) | 9.27 (1.39 to 29.96) | 0.53 (0.15 to 1.09) | -4.65% (-7.82 to -1.37) |
| Estonia | 27 (6 to 53) | 15 (4 to 31) | -0.42% (-0.46 to -0.39) | 1.7 (0.41 to 3.4) | 1.17 (0.28 to 2.33) | -1.43% (-1.54 to -1.32) | 1.71 (0.41 to 3.43) | 1.27 (0.29 to 2.57) | -1.19% (-1.29 to -1.09) |
| Eswatini | 3 (1 to 6) | 5 (1 to 10) | 0.61% (0.5 to 0.74) | 0.38 (0.1 to 0.76) | 0.43 (0.11 to 0.87) | 0.5% (0.44 to 0.56) | 0.39 (0.11 to 0.79) | 0.43 (0.12 to 0.88) | 0.38% (0.28 to 0.48) |
| Ethiopia | 2677 (554 to 7628) | 426 (98 to 860) | -0.84% (-0.93 to -0.56) | 5.21 (1.08 to 14.84) | 0.4 (0.09 to 0.8) | -3.81% (-6.43 to -1.13) | 4.71 (1.02 to 13.17) | 0.39 (0.1 to 0.77) | -3.77% (-6.23 to -1.24) |
| Fiji | 2 (1 to 5) | 3 (1 to 6) | 0.26% (0.18 to 0.35) | 0.32 (0.07 to 0.65) | 0.33 (0.08 to 0.69) | 0.08% (-0.19 to 0.36) | 0.31 (0.07 to 0.62) | 0.33 (0.08 to 0.69) | 0.21% (-0.06 to 0.49) |
| Finland | 74 (18 to 155) | 79 (21 to 165) | 0.06% (0 to 0.17) | 1.48 (0.36 to 3.1) | 1.43 (0.37 to 2.98) | -0.11% (-0.47 to 0.24) | 1.52 (0.36 to 3.2) | 1.49 (0.36 to 3.22) | 0% (-0.36 to 0.36) |
| France | 707 (176 to 1448) | 803 (218 to 1655) | 0.14% (0.06 to 0.26) | 1.22 (0.31 to 2.51) | 1.21 (0.33 to 2.5) | -0.09% (-0.12 to -0.06) | 1.21 (0.29 to 2.53) | 1.17 (0.29 to 2.45) | -0.16% (-0.19 to -0.13) |
| Gabon | 3 (1 to 7) | 6 (1 to 12) | 0.74% (0.67 to 0.83) | 0.34 (0.08 to 0.66) | 0.33 (0.08 to 0.66) | -0.14% (-0.25 to -0.04) | 0.34 (0.09 to 0.67) | 0.34 (0.09 to 0.67) | -0.1% (-0.2 to -0.01) |
| Gambia | 4 (1 to 7) | 8 (2 to 17) | 1.39% (1.28 to 1.52) | 0.35 (0.09 to 0.7) | 0.37 (0.1 to 0.75) | 0.21% (0.06 to 0.36) | 0.39 (0.11 to 0.79) | 0.41 (0.12 to 0.84) | 0.24% (0.11 to 0.37) |
| Georgia | 59 (13 to 118) | 39 (9 to 80) | -0.33% (-0.36 to -0.28) | 1.06 (0.24 to 2.15) | 1.08 (0.25 to 2.19) | -0.39% (-1.14 to 0.36) | 1.08 (0.25 to 2.17) | 1.18 (0.26 to 2.41) | -0.16% (-0.94 to 0.61) |
| Germany | 770 (190 to 1585) | 841 (230 to 1705) | 0.09% (0.02 to 0.22) | 0.96 (0.24 to 1.98) | 0.99 (0.27 to 2.01) | -0.05% (-0.21 to 0.12) | 1 (0.24 to 2.11) | 1.02 (0.25 to 2.18) | -0.04% (-0.17 to 0.09) |
| Ghana | 59 (16 to 120) | 147 (39 to 307) | 1.49% (1.34 to 1.66) | 0.39 (0.1 to 0.8) | 0.47 (0.12 to 0.97) | 0.64% (0.56 to 0.71) | 0.43 (0.12 to 0.89) | 0.5 (0.14 to 1.04) | 0.54% (0.47 to 0.61) |
| Greece | 100 (24 to 207) | 83 (21 to 168) | -0.17% (-0.21 to -0.1) | 0.96 (0.23 to 1.99) | 0.8 (0.21 to 1.63) | -0.77% (-0.84 to -0.7) | 1.02 (0.23 to 2.17) | 0.94 (0.22 to 1.97) | -0.41% (-0.47 to -0.36) |
| Greenland | 1 (0 to 2) | 1 (0 to 1) | -0.18% (-0.25 to -0.03) | 1.56 (0.42 to 3.13) | 1.26 (0.39 to 2.52) | -0.79% (-0.9 to -0.67) | 1.59 (0.47 to 3.16) | 1.21 (0.38 to 2.4) | -1.1% (-1.17 to -1.03) |
| Grenada | 0 (0 to 1) | 1 (0 to 1) | 0.4% (0.29 to 0.52) | 0.5 (0.11 to 1.02) | 0.58 (0.14 to 1.16) | 0.34% (0.07 to 0.61) | 0.48 (0.11 to 0.98) | 0.59 (0.14 to 1.19) | 0.54% (0.28 to 0.81) |
| Guam | 1 (0 to 1) | 1 (0 to 2) | 0.24% (0.15 to 0.38) | 0.45 (0.12 to 0.94) | 0.45 (0.12 to 0.93) | -0.09% (-0.23 to 0.06) | 0.45 (0.12 to 0.92) | 0.46 (0.12 to 0.93) | 0.01% (-0.13 to 0.15) |
| Guatemala | 92 (20 to 209) | 132 (30 to 271) | 0.43% (-0.16 to 1.03) | 1.15 (0.25 to 2.62) | 0.74 (0.17 to 1.52) | -0.81% (-1.16 to -0.45) | 1.04 (0.24 to 2.35) | 0.71 (0.17 to 1.46) | -0.61% (-0.95 to -0.28) |
| Guinea | 24 (6 to 48) | 51 (13 to 104) | 1.13% (1.02 to 1.24) | 0.38 (0.1 to 0.78) | 0.4 (0.11 to 0.82) | 0.01% (-0.69 to 0.71) | 0.41 (0.11 to 0.84) | 0.45 (0.13 to 0.93) | 0.24% (-0.39 to 0.88) |
| Guinea-Bissau | 4 (1 to 9) | 8 (2 to 17) | 0.87% (0.79 to 0.97) | 0.44 (0.11 to 0.91) | 0.44 (0.11 to 0.91) | -0.69% (-2.11 to 0.76) | 0.48 (0.13 to 1.02) | 0.48 (0.13 to 1) | -0.62% (-1.89 to 0.66) |
| Guyana | 5 (1 to 10) | 5 (1 to 10) | 0.07% (-0.01 to 0.17) | 0.62 (0.14 to 1.24) | 0.66 (0.15 to 1.3) | 0.09% (-0.19 to 0.38) | 0.59 (0.14 to 1.15) | 0.66 (0.15 to 1.29) | 0.24% (-0.05 to 0.52) |
| Haiti | 33 (7 to 67) | 61 (14 to 123) | 0.85% (0.74 to 0.98) | 0.52 (0.11 to 1.05) | 0.49 (0.11 to 0.99) | 0.18% (-2.36 to 2.78) | 0.49 (0.12 to 1.02) | 0.47 (0.11 to 0.96) | 0.26% (-2.32 to 2.9) |
| Honduras | 31 (7 to 63) | 58 (13 to 118) | 0.89% (0.76 to 1.05) | 0.66 (0.14 to 1.35) | 0.59 (0.13 to 1.2) | -1.09% (-2.35 to 0.19) | 0.58 (0.13 to 1.18) | 0.57 (0.13 to 1.14) | -0.86% (-2.15 to 0.46) |
| Hungary | 186 (47 to 373) | 137 (35 to 274) | -0.26% (-0.3 to -0.23) | 1.79 (0.45 to 3.59) | 1.41 (0.37 to 2.83) | -1.14% (-1.26 to -1.02) | 1.74 (0.42 to 3.51) | 1.5 (0.37 to 3.08) | -0.79% (-0.92 to -0.67) |
| Iceland | 2 (1 to 5) | 3 (1 to 7) | 0.34% (0.27 to 0.45) | 0.98 (0.24 to 2.04) | 0.97 (0.24 to 2.02) | -0.14% (-0.26 to -0.03) | 0.98 (0.23 to 2.06) | 1.02 (0.24 to 2.17) | 0.01% (-0.09 to 0.12) |
| India | 6441 (1532 to 13125) | 10089 (2555 to 20153) | 0.57% (0.39 to 0.75) | 0.75 (0.18 to 1.53) | 0.73 (0.18 to 1.45) | -0.08% (-0.19 to 0.03) | 0.79 (0.2 to 1.6) | 0.73 (0.19 to 1.46) | -0.2% (-0.29 to -0.1) |
| Indonesia | 977 (256 to 1984) | 1218 (339 to 2437) | 0.25% (0.2 to 0.33) | 0.53 (0.14 to 1.07) | 0.47 (0.13 to 0.94) | -0.58% (-0.88 to -0.29) | 0.54 (0.15 to 1.08) | 0.48 (0.14 to 0.96) | -0.61% (-0.9 to -0.31) |
| Iran (Islamic Republic of) | 731 (237 to 1417) | 449 (105 to 903) | -0.39% (-0.63 to -0.16) | 1.25 (0.41 to 2.42) | 0.53 (0.12 to 1.07) | -1.44% (-1.94 to -0.93) | 1.28 (0.43 to 2.49) | 0.53 (0.13 to 1.06) | -1.37% (-1.94 to -0.78) |
| Iraq | 224 (48 to 495) | 384 (84 to 784) | 0.72% (0.42 to 1.03) | 1.27 (0.27 to 2.81) | 0.91 (0.2 to 1.86) | 4.11% (1.8 to 6.48) | 1.2 (0.26 to 2.62) | 0.84 (0.19 to 1.71) | 3.9% (1.55 to 6.31) |
| Ireland | 35 (8 to 73) | 47 (11 to 98) | 0.34% (0.26 to 0.45) | 0.96 (0.23 to 2.02) | 0.95 (0.23 to 1.99) | -0.14% (-0.24 to -0.04) | 0.98 (0.23 to 2.04) | 1.02 (0.24 to 2.18) | 0.05% (-0.04 to 0.15) |
| Israel | 48 (11 to 98) | 83 (20 to 175) | 0.74% (0.5 to 0.94) | 0.96 (0.22 to 1.97) | 0.89 (0.21 to 1.88) | 0.02% (-0.68 to 0.73) | 0.94 (0.22 to 1.92) | 0.9 (0.22 to 1.91) | 0.16% (-0.55 to 0.87) |
| Italy | 608 (147 to 1251) | 501 (135 to 1002) | -0.18% (-0.24 to -0.07) | 1.07 (0.26 to 2.2) | 0.83 (0.22 to 1.66) | -1.73% (-2.08 to -1.38) | 1.07 (0.27 to 2.25) | 0.86 (0.21 to 1.81) | -1.61% (-1.97 to -1.24) |
| Jamaica | 14 (3 to 28) | 15 (3 to 29) | 0.07% (-0.05 to 0.19) | 0.57 (0.13 to 1.17) | 0.52 (0.12 to 1.03) | -0.36% (-0.47 to -0.24) | 0.54 (0.13 to 1.1) | 0.52 (0.12 to 1.05) | -0.13% (-0.24 to -0.01) |
| Japan | 1225 (326 to 2473) | 1138 (358 to 2225) | -0.07% (-0.14 to 0.13) | 0.97 (0.26 to 1.96) | 0.89 (0.28 to 1.74) | -0.41% (-0.5 to -0.31) | 0.99 (0.25 to 1.99) | 0.91 (0.24 to 1.9) | -0.34% (-0.45 to -0.23) |
| Jordan | 20 (4 to 40) | 61 (13 to 129) | 2.05% (1.82 to 2.31) | 0.53 (0.11 to 1.06) | 0.53 (0.11 to 1.1) | 0.11% (-0.28 to 0.52) | 0.48 (0.11 to 0.95) | 0.49 (0.11 to 1.03) | 0.25% (-0.14 to 0.65) |
| Kazakhstan | 170 (39 to 345) | 179 (42 to 360) | 0.05% (0.01 to 0.1) | 1.04 (0.24 to 2.11) | 0.97 (0.23 to 1.96) | 0.05% (-0.08 to 0.19) | 1 (0.23 to 2.02) | 0.97 (0.23 to 1.95) | 0.16% (0.05 to 0.27) |
| Kenya | 90 (20 to 179) | 197 (46 to 395) | 1.2% (1.13 to 1.3) | 0.39 (0.09 to 0.77) | 0.39 (0.09 to 0.79) | 0.22% (-0.16 to 0.59) | 0.38 (0.1 to 0.76) | 0.39 (0.1 to 0.78) | 0.2% (-0.13 to 0.52) |
| Kiribati | 0 (0 to 0) | 0 (0 to 1) | 0.79% (0.67 to 0.91) | 0.25 (0.06 to 0.51) | 0.28 (0.06 to 0.59) | -0.63% (-1.45 to 0.2) | 0.24 (0.06 to 0.48) | 0.27 (0.06 to 0.55) | -0.6% (-1.37 to 0.18) |
| Kuwait | 57 (12 to 156) | 29 (6 to 59) | -0.5% (-0.78 to 0.17) | 3.23 (0.7 to 8.84) | 0.65 (0.15 to 1.34) | -2.17% (-3.66 to -0.66) | 2.84 (0.63 to 7.79) | 0.62 (0.14 to 1.29) | -2.24% (-3.95 to -0.5) |
| Kyrgyzstan | 44 (10 to 89) | 49 (11 to 101) | 0.13% (0.06 to 0.2) | 0.98 (0.22 to 1.98) | 0.76 (0.17 to 1.55) | -1.04% (-1.32 to -0.77) | 0.94 (0.21 to 1.89) | 0.73 (0.17 to 1.48) | -1.01% (-1.29 to -0.74) |
| Lao People's Democratic Republic | 27 (6 to 64) | 27 (6 to 53) | 0% (-0.46 to 0.5) | 0.64 (0.15 to 1.55) | 0.37 (0.09 to 0.74) | -0.51% (-0.91 to -0.12) | 0.61 (0.14 to 1.42) | 0.36 (0.09 to 0.73) | -0.53% (-0.89 to -0.17) |
| Latvia | 51 (12 to 102) | 23 (6 to 46) | -0.54% (-0.56 to -0.51) | 1.9 (0.46 to 3.83) | 1.21 (0.3 to 2.42) | -1.69% (-1.86 to -1.52) | 1.9 (0.45 to 3.8) | 1.29 (0.31 to 2.63) | -1.49% (-1.63 to -1.35) |
| Lebanon | 47 (10 to 122) | 29 (7 to 60) | -0.39% (-0.7 to 0.14) | 1.43 (0.29 to 3.71) | 0.55 (0.13 to 1.16) | -0.71% (-1.68 to 0.28) | 1.35 (0.28 to 3.46) | 0.56 (0.13 to 1.17) | -0.58% (-1.54 to 0.4) |
| Lesotho | 7 (2 to 14) | 10 (3 to 20) | 0.38% (0.27 to 0.5) | 0.39 (0.1 to 0.78) | 0.46 (0.12 to 0.95) | 0.65% (0.31 to 1) | 0.39 (0.1 to 0.79) | 0.46 (0.13 to 0.94) | 0.6% (0.29 to 0.91) |
| Liberia | 133 (19 to 420) | 17 (4 to 34) | -0.87% (-0.95 to -0.56) | 6.79 (0.98 to 21.4) | 0.35 (0.09 to 0.7) | -8.06% (-11.07 to -4.94) | 6.41 (0.96 to 20.28) | 0.38 (0.11 to 0.77) | -7.5% (-10.37 to -4.53) |
| Libya | 24 (5 to 47) | 72 (16 to 164) | 2.04% (1.11 to 4.32) | 0.56 (0.12 to 1.12) | 1.07 (0.24 to 2.44) | 3.35% (1.08 to 5.67) | 0.54 (0.13 to 1.08) | 1.05 (0.23 to 2.4) | 3.35% (1.09 to 5.67) |
| Lithuania | 65 (16 to 130) | 38 (10 to 76) | -0.42% (-0.45 to -0.36) | 1.77 (0.43 to 3.55) | 1.35 (0.34 to 2.71) | -0.88% (-1.02 to -0.75) | 1.75 (0.42 to 3.5) | 1.38 (0.32 to 2.77) | -0.8% (-0.9 to -0.69) |
| Luxembourg | 5 (1 to 10) | 7 (2 to 14) | 0.41% (0.29 to 0.56) | 1.23 (0.3 to 2.51) | 1.07 (0.28 to 2.19) | -0.68% (-0.74 to -0.62) | 1.26 (0.29 to 2.62) | 1.11 (0.27 to 2.33) | -0.66% (-0.74 to -0.57) |
| Madagascar | 60 (15 to 122) | 134 (35 to 275) | 1.23% (1.11 to 1.42) | 0.5 (0.13 to 1.02) | 0.5 (0.13 to 1.03) | -0.03% (-0.2 to 0.15) | 0.52 (0.14 to 1.06) | 0.51 (0.14 to 1.05) | -0.06% (-0.2 to 0.08) |
| Malawi | 40 (10 to 81) | 82 (21 to 167) | 1.03% (0.93 to 1.14) | 0.42 (0.11 to 0.85) | 0.44 (0.11 to 0.91) | 0.02% (-0.13 to 0.18) | 0.44 (0.12 to 0.89) | 0.45 (0.13 to 0.92) | -0.03% (-0.17 to 0.11) |
| Malaysia | 69 (17 to 139) | 136 (34 to 276) | 0.96% (0.83 to 1.15) | 0.39 (0.1 to 0.79) | 0.44 (0.11 to 0.88) | 0.22% (0.09 to 0.34) | 0.39 (0.1 to 0.78) | 0.43 (0.11 to 0.87) | 0.17% (0.05 to 0.3) |
| Maldives | 1 (0 to 2) | 2 (0 to 4) | 1.41% (1.16 to 1.74) | 0.37 (0.09 to 0.75) | 0.4 (0.1 to 0.81) | 0.07% (-0.68 to 0.82) | 0.37 (0.09 to 0.74) | 0.4 (0.1 to 0.8) | 0.04% (-0.71 to 0.8) |
| Mali | 56 (12 to 134) | 121 (28 to 267) | 1.18% (0.96 to 1.41) | 0.64 (0.14 to 1.54) | 0.55 (0.13 to 1.22) | -1.77% (-4.05 to 0.58) | 0.66 (0.16 to 1.5) | 0.58 (0.15 to 1.23) | -1.55% (-3.65 to 0.6) |
| Malta | 4 (1 to 8) | 4 (1 to 9) | 0.2% (0.12 to 0.35) | 1.01 (0.24 to 2.12) | 1.02 (0.26 to 2.09) | 0.22% (0.15 to 0.29) | 1.05 (0.25 to 2.24) | 1.14 (0.27 to 2.44) | 0.58% (0.49 to 0.67) |
| Marshall Islands | 0 (0 to 0) | 0 (0 to 0) | 0.42% (0.33 to 0.53) | 0.31 (0.07 to 0.64) | 0.36 (0.08 to 0.73) | 0.46% (0.4 to 0.53) | 0.32 (0.08 to 0.63) | 0.36 (0.09 to 0.71) | 0.34% (0.26 to 0.43) |
| Mauritania | 11 (3 to 22) | 17 (5 to 36) | 0.64% (0.37 to 0.83) | 0.51 (0.13 to 1.05) | 0.43 (0.12 to 0.89) | -0.22% (-0.44 to 0) | 0.55 (0.15 to 1.15) | 0.47 (0.13 to 0.97) | -0.25% (-0.46 to -0.04) |
| Mauritius | 4 (1 to 9) | 6 (2 to 13) | 0.41% (0.31 to 0.57) | 0.41 (0.1 to 0.83) | 0.49 (0.14 to 1.02) | 0.71% (0.64 to 0.78) | 0.4 (0.11 to 0.81) | 0.49 (0.13 to 1) | 0.74% (0.67 to 0.8) |
| Mexico | 774 (193 to 1594) | 930 (252 to 1893) | 0.2% (0.15 to 0.31) | 0.9 (0.23 to 1.86) | 0.74 (0.2 to 1.52) | 1.22% (0.68 to 1.76) | 0.9 (0.24 to 1.82) | 0.74 (0.2 to 1.52) | 1.1% (0.61 to 1.6) |
| Micronesia (Federated States of) | 0 (0 to 1) | 0 (0 to 1) | 0.16% (0.07 to 0.26) | 0.32 (0.07 to 0.64) | 0.38 (0.09 to 0.77) | 0.54% (-0.11 to 1.2) | 0.32 (0.08 to 0.63) | 0.37 (0.09 to 0.75) | 0.47% (-0.18 to 1.12) |
| Monaco | 0 (0 to 1) | 0 (0 to 1) | 0.39% (0.33 to 0.46) | 0.89 (0.28 to 1.75) | 1 (0.32 to 1.99) | 0.45% (0.4 to 0.49) | 0.92 (0.25 to 1.86) | 1.04 (0.28 to 2.14) | 0.49% (0.43 to 0.56) |
| Mongolia | 19 (4 to 38) | 34 (8 to 69) | 0.81% (0.69 to 0.93) | 0.88 (0.2 to 1.76) | 1.01 (0.23 to 2.05) | 0.67% (0.45 to 0.89) | 0.83 (0.19 to 1.67) | 0.98 (0.22 to 1.99) | 0.71% (0.51 to 0.91) |
| Montenegro | 9 (2 to 19) | 9 (2 to 18) | -0.08% (-0.13 to -0.01) | 1.5 (0.35 to 3.09) | 1.39 (0.33 to 2.82) | -0.31% (-0.38 to -0.24) | 1.49 (0.35 to 3.07) | 1.49 (0.36 to 3.08) | -0.07% (-0.15 to 0.01) |
| Morocco | 160 (35 to 320) | 225 (51 to 460) | 0.4% (0.29 to 0.54) | 0.63 (0.14 to 1.27) | 0.62 (0.14 to 1.28) | -0.23% (-0.33 to -0.12) | 0.59 (0.13 to 1.17) | 0.62 (0.14 to 1.27) | -0.02% (-0.13 to 0.09) |
| Mozambique | 141 (31 to 375) | 183 (47 to 368) | 0.29% (-0.31 to 1.19) | 1.08 (0.24 to 2.87) | 0.62 (0.16 to 1.25) | -0.68% (-1.47 to 0.11) | 1.07 (0.26 to 2.71) | 0.67 (0.19 to 1.36) | -0.45% (-1.15 to 0.26) |
| Myanmar | 242 (56 to 483) | 290 (69 to 588) | 0.2% (0.09 to 0.33) | 0.59 (0.14 to 1.18) | 0.53 (0.13 to 1.08) | -0.11% (-1.5 to 1.31) | 0.56 (0.13 to 1.1) | 0.53 (0.13 to 1.08) | 0.11% (-1.3 to 1.54) |
| Namibia | 6 (1 to 12) | 11 (3 to 21) | 0.83% (0.72 to 0.94) | 0.41 (0.11 to 0.82) | 0.44 (0.12 to 0.88) | 0.03% (-0.37 to 0.42) | 0.42 (0.11 to 0.86) | 0.44 (0.12 to 0.89) | -0.08% (-0.45 to 0.29) |
| Nauru | 0 (0 to 0) | 0 (0 to 0) | 0.22% (0.14 to 0.31) | 0.4 (0.1 to 0.84) | 0.48 (0.12 to 0.98) | 0.52% (0.45 to 0.59) | 0.43 (0.12 to 0.88) | 0.49 (0.13 to 0.98) | 0.37% (0.3 to 0.45) |
| Nepal | 119 (26 to 246) | 201 (46 to 415) | 0.69% (0.61 to 0.8) | 0.61 (0.14 to 1.26) | 0.66 (0.15 to 1.36) | 0.16% (-0.77 to 1.09) | 0.62 (0.15 to 1.27) | 0.65 (0.16 to 1.34) | 0.05% (-0.8 to 0.92) |
| Netherlands | 114 (29 to 234) | 163 (47 to 328) | 0.42% (0.29 to 0.69) | 0.77 (0.2 to 1.56) | 0.95 (0.27 to 1.91) | 0.58% (0.2 to 0.96) | 0.78 (0.19 to 1.62) | 0.89 (0.22 to 1.84) | 0.26% (-0.01 to 0.53) |
| New Zealand | 66 (16 to 135) | 85 (21 to 173) | 0.3% (0.21 to 0.44) | 1.93 (0.46 to 3.94) | 1.9 (0.47 to 3.85) | -0.02% (-0.06 to 0.02) | 1.95 (0.46 to 4.01) | 2.03 (0.48 to 4.12) | 0.14% (0.09 to 0.2) |
| Nicaragua | 26 (6 to 53) | 35 (8 to 73) | 0.33% (0.12 to 0.51) | 0.68 (0.15 to 1.37) | 0.54 (0.12 to 1.13) | -0.89% (-1.39 to -0.4) | 0.59 (0.14 to 1.18) | 0.53 (0.13 to 1.09) | -0.55% (-1.18 to 0.09) |
| Niger | 38 (9 to 79) | 101 (25 to 205) | 1.64% (1.47 to 1.8) | 0.48 (0.12 to 0.99) | 0.43 (0.11 to 0.88) | -0.01% (-0.44 to 0.43) | 0.51 (0.13 to 1.04) | 0.48 (0.13 to 0.98) | 0.17% (-0.19 to 0.52) |
| Nigeria | 349 (93 to 695) | 926 (240 to 1898) | 1.65% (1.38 to 2.23) | 0.39 (0.1 to 0.77) | 0.43 (0.11 to 0.88) | 1.03% (0.55 to 1.51) | 0.41 (0.12 to 0.83) | 0.46 (0.13 to 0.93) | 0.99% (0.57 to 1.41) |
| Niue | 0 (0 to 0) | 0 (0 to 0) | -0.15% (-0.21 to -0.06) | 0.4 (0.11 to 0.81) | 0.47 (0.13 to 0.94) | 0.55% (-0.11 to 1.22) | 0.41 (0.11 to 0.85) | 0.47 (0.13 to 0.95) | 0.44% (-0.23 to 1.11) |
| North Macedonia | 29 (7 to 61) | 27 (7 to 55) | -0.09% (-0.26 to 0.1) | 1.46 (0.33 to 3.04) | 1.24 (0.3 to 2.54) | -0.76% (-0.89 to -0.62) | 1.44 (0.33 to 3) | 1.35 (0.32 to 2.8) | -0.45% (-0.6 to -0.3) |
| Northern Mariana Islands | 0 (0 to 1) | 0 (0 to 0) | -0.08% (-0.17 to 0.07) | 0.58 (0.15 to 1.19) | 0.57 (0.16 to 1.15) | -0.31% (-0.53 to -0.08) | 0.57 (0.16 to 1.16) | 0.58 (0.17 to 1.16) | -0.07% (-0.23 to 0.1) |
| Norway | 54 (17 to 106) | 67 (23 to 136) | 0.25% (0.17 to 0.36) | 1.27 (0.41 to 2.5) | 1.26 (0.43 to 2.54) | -0.17% (-0.23 to -0.11) | 1.17 (0.34 to 2.34) | 1.14 (0.34 to 2.33) | -0.19% (-0.27 to -0.12) |
| Oman | 13 (3 to 27) | 34 (8 to 69) | 1.6% (1.39 to 1.84) | 0.68 (0.15 to 1.37) | 0.74 (0.17 to 1.52) | 0.35% (0.27 to 0.43) | 0.67 (0.16 to 1.35) | 0.7 (0.17 to 1.42) | 0.02% (-0.06 to 0.11) |
| Pakistan | 386 (88 to 772) | 927 (218 to 1875) | 1.4% (1.24 to 1.56) | 0.34 (0.08 to 0.68) | 0.41 (0.1 to 0.84) | 1.39% (0.88 to 1.9) | 0.35 (0.09 to 0.7) | 0.41 (0.1 to 0.83) | 1.23% (0.74 to 1.72) |
| Palau | 0 (0 to 0) | 0 (0 to 0) | 0.32% (0.24 to 0.48) | 0.68 (0.18 to 1.36) | 0.77 (0.22 to 1.54) | 0.28% (0.21 to 0.35) | 0.68 (0.19 to 1.37) | 0.77 (0.22 to 1.54) | 0.31% (0.26 to 0.35) |
| Palestine | 34 (7 to 86) | 33 (7 to 67) | -0.02% (-0.47 to 0.71) | 1.63 (0.34 to 4.16) | 0.66 (0.15 to 1.35) | -1.63% (-5.07 to 1.95) | 1.43 (0.32 to 3.54) | 0.61 (0.14 to 1.23) | -1.52% (-4.88 to 1.97) |
| Panama | 15 (3 to 30) | 23 (5 to 48) | 0.59% (0.52 to 0.68) | 0.61 (0.14 to 1.27) | 0.56 (0.13 to 1.15) | -0.42% (-0.52 to -0.33) | 0.58 (0.14 to 1.18) | 0.56 (0.13 to 1.16) | -0.22% (-0.31 to -0.12) |
| Papua New Guinea | 15 (3 to 30) | 40 (9 to 81) | 1.71% (1.29 to 2.02) | 0.36 (0.08 to 0.73) | 0.4 (0.09 to 0.82) | -0.12% (-0.99 to 0.74) | 0.36 (0.09 to 0.72) | 0.41 (0.1 to 0.82) | -0.06% (-0.92 to 0.81) |
| Paraguay | 29 (6 to 61) | 47 (10 to 96) | 0.61% (0.54 to 0.71) | 0.71 (0.15 to 1.5) | 0.67 (0.15 to 1.38) | -0.22% (-0.31 to -0.13) | 0.67 (0.15 to 1.39) | 0.65 (0.15 to 1.34) | -0.12% (-0.21 to -0.02) |
| Peru | 220 (48 to 510) | 182 (43 to 363) | -0.17% (-0.52 to 0.24) | 1.01 (0.22 to 2.35) | 0.54 (0.13 to 1.07) | -1.44% (-2.05 to -0.82) | 0.93 (0.21 to 2.12) | 0.53 (0.13 to 1.06) | -1.2% (-1.78 to -0.61) |
| Philippines | 341 (84 to 677) | 459 (108 to 925) | 0.35% (0.08 to 0.59) | 0.54 (0.13 to 1.07) | 0.41 (0.1 to 0.83) | -0.35% (-0.83 to 0.13) | 0.52 (0.13 to 1.02) | 0.41 (0.1 to 0.81) | -0.28% (-0.76 to 0.2) |
| Poland | 605 (147 to 1223) | 549 (134 to 1101) | -0.09% (-0.17 to 0) | 1.59 (0.39 to 3.2) | 1.43 (0.35 to 2.86) | -0.38% (-0.44 to -0.32) | 1.6 (0.39 to 3.25) | 1.5 (0.36 to 3.05) | -0.26% (-0.37 to -0.15) |
| Portugal | 92 (21 to 185) | 70 (18 to 138) | -0.24% (-0.3 to -0.09) | 0.9 (0.21 to 1.82) | 0.65 (0.17 to 1.3) | -1.52% (-1.64 to -1.39) | 0.93 (0.21 to 1.87) | 0.69 (0.17 to 1.44) | -1.41% (-1.53 to -1.28) |
| Puerto Rico | 23 (6 to 46) | 24 (7 to 48) | 0.05% (-0.06 to 0.26) | 0.64 (0.17 to 1.28) | 0.69 (0.21 to 1.35) | 0.61% (0.21 to 1.01) | 0.63 (0.17 to 1.28) | 0.68 (0.19 to 1.38) | 0.63% (0.2 to 1.05) |
| Qatar | 4 (1 to 9) | 28 (6 to 58) | 5.64% (5.09 to 6.18) | 0.95 (0.21 to 1.94) | 0.98 (0.21 to 2.02) | 0.55% (0.33 to 0.77) | 0.82 (0.19 to 1.65) | 0.81 (0.19 to 1.7) | 0.15% (0.02 to 0.28) |
| Republic of Korea | 555 (124 to 1111) | 547 (138 to 1089) | -0.01% (-0.09 to 0.15) | 1.25 (0.28 to 2.51) | 1.02 (0.26 to 2.04) | -0.99% (-1.1 to -0.88) | 1.19 (0.27 to 2.37) | 1.06 (0.25 to 2.18) | -0.68% (-0.78 to -0.57) |
| Republic of Moldova | 61 (14 to 122) | 37 (9 to 74) | -0.39% (-0.43 to -0.34) | 1.37 (0.32 to 2.74) | 1.01 (0.24 to 2) | -1.42% (-1.7 to -1.14) | 1.37 (0.31 to 2.73) | 1.07 (0.25 to 2.17) | -1.24% (-1.56 to -0.93) |
| Romania | 404 (91 to 823) | 256 (60 to 518) | -0.37% (-0.4 to -0.31) | 1.73 (0.39 to 3.52) | 1.33 (0.31 to 2.7) | -1.11% (-1.22 to -1) | 1.75 (0.39 to 3.58) | 1.47 (0.35 to 3.05) | -0.79% (-0.92 to -0.65) |
| Russian Federation | 2456 (599 to 4898) | 1943 (483 to 3950) | -0.21% (-0.26 to -0.15) | 1.63 (0.4 to 3.24) | 1.32 (0.33 to 2.69) | -0.64% (-1.02 to -0.24) | 1.64 (0.39 to 3.25) | 1.37 (0.32 to 2.8) | -0.61% (-0.92 to -0.3) |
| Rwanda | 140 (26 to 397) | 63 (17 to 127) | -0.55% (-0.8 to 0.01) | 1.96 (0.36 to 5.53) | 0.49 (0.13 to 1) | -6.03% (-8.68 to -3.31) | 1.86 (0.37 to 5.07) | 0.51 (0.14 to 1.04) | -5.77% (-8.36 to -3.1) |
| Saint Kitts and Nevis | 0 (0 to 1) | 0 (0 to 1) | 0.55% (0.45 to 0.66) | 0.6 (0.16 to 1.23) | 0.64 (0.17 to 1.33) | 0.1% (-0.05 to 0.25) | 0.6 (0.16 to 1.24) | 0.66 (0.18 to 1.36) | 0.15% (0.01 to 0.29) |
| Saint Lucia | 1 (0 to 1) | 1 (0 to 2) | 0.28% (0.16 to 0.41) | 0.49 (0.11 to 0.99) | 0.49 (0.12 to 0.98) | -0.02% (-0.19 to 0.14) | 0.47 (0.11 to 0.93) | 0.52 (0.12 to 1.03) | 0.33% (0.17 to 0.49) |
| Saint Vincent and the Grenadines | 1 (0 to 1) | 1 (0 to 1) | 0.1% (0.02 to 0.21) | 0.49 (0.11 to 0.97) | 0.53 (0.13 to 1.04) | 0.13% (-0.08 to 0.35) | 0.46 (0.11 to 0.92) | 0.54 (0.13 to 1.07) | 0.41% (0.2 to 0.62) |
| Samoa | 1 (0 to 1) | 1 (0 to 2) | 0.3% (0.09 to 0.45) | 0.38 (0.09 to 0.73) | 0.38 (0.09 to 0.79) | 0.51% (-0.78 to 1.82) | 0.38 (0.1 to 0.72) | 0.38 (0.09 to 0.77) | 0.55% (-0.73 to 1.85) |
| San Marino | 0 (0 to 0) | 0 (0 to 1) | 0.53% (0.43 to 0.72) | 0.96 (0.27 to 1.92) | 1.04 (0.33 to 2.08) | 0.32% (0.26 to 0.38) | 0.98 (0.26 to 1.99) | 1.06 (0.28 to 2.16) | 0.33% (0.26 to 0.4) |
| Sao Tome and Principe | 1 (0 to 1) | 1 (0 to 2) | 0.91% (0.8 to 1.05) | 0.45 (0.12 to 0.92) | 0.51 (0.14 to 1.05) | 0.4% (0.34 to 0.46) | 0.47 (0.13 to 0.95) | 0.55 (0.16 to 1.11) | 0.55% (0.5 to 0.6) |
| Saudi Arabia | 199 (41 to 410) | 604 (131 to 1251) | 2.03% (1.63 to 2.45) | 1.24 (0.25 to 2.56) | 1.69 (0.37 to 3.5) | 1.26% (1.11 to 1.41) | 1.19 (0.27 to 2.42) | 1.47 (0.33 to 3.02) | 0.82% (0.68 to 0.96) |
| Senegal | 32 (8 to 64) | 58 (15 to 116) | 0.81% (0.49 to 1.05) | 0.42 (0.11 to 0.84) | 0.38 (0.1 to 0.77) | -0.65% (-1.09 to -0.21) | 0.45 (0.12 to 0.91) | 0.42 (0.12 to 0.84) | -0.51% (-0.88 to -0.15) |
| Serbia | 116 (28 to 233) | 109 (26 to 226) | -0.06% (-0.14 to 0.03) | 1.23 (0.3 to 2.48) | 1.24 (0.3 to 2.59) | -0.87% (-1.95 to 0.21) | 1.28 (0.31 to 2.58) | 1.37 (0.32 to 2.85) | -0.67% (-1.8 to 0.46) |
| Seychelles | 0 (0 to 1) | 1 (0 to 1) | 0.49% (0.39 to 0.63) | 0.46 (0.12 to 0.94) | 0.49 (0.13 to 1) | 0.06% (-0.07 to 0.2) | 0.47 (0.13 to 0.95) | 0.48 (0.13 to 0.98) | -0.06% (-0.21 to 0.09) |
| Sierra Leone | 14 (4 to 28) | 32 (8 to 64) | 1.28% (1.16 to 1.39) | 0.38 (0.1 to 0.76) | 0.38 (0.1 to 0.77) | -6.03% (-8.7 to -3.27) | 0.4 (0.11 to 0.82) | 0.42 (0.12 to 0.86) | -5.46% (-8 to -2.84) |
| Singapore | 31 (7 to 64) | 47 (11 to 95) | 0.49% (0.41 to 0.64) | 1.03 (0.24 to 2.09) | 0.83 (0.2 to 1.68) | -0.91% (-1 to -0.81) | 0.96 (0.22 to 1.96) | 0.89 (0.21 to 1.84) | -0.43% (-0.52 to -0.34) |
| Slovakia | 91 (22 to 188) | 88 (22 to 175) | -0.04% (-0.09 to 0.03) | 1.73 (0.41 to 3.56) | 1.61 (0.4 to 3.22) | -0.31% (-0.38 to -0.24) | 1.72 (0.4 to 3.56) | 1.67 (0.4 to 3.44) | -0.17% (-0.27 to -0.06) |
| Slovenia | 38 (9 to 77) | 36 (10 to 73) | -0.04% (-0.11 to 0.08) | 1.92 (0.47 to 3.91) | 1.75 (0.47 to 3.5) | -0.01% (-0.14 to 0.12) | 1.9 (0.45 to 3.9) | 1.79 (0.44 to 3.7) | 0.16% (0.01 to 0.3) |
| Solomon Islands | 1 (0 to 3) | 3 (1 to 7) | 1.29% (1.13 to 1.45) | 0.44 (0.1 to 0.92) | 0.52 (0.12 to 1.09) | 0.58% (0.31 to 0.85) | 0.46 (0.11 to 0.96) | 0.54 (0.13 to 1.12) | 0.52% (0.26 to 0.78) |
| Somalia | 135 (24 to 418) | 161 (34 to 378) | 0.19% (-0.15 to 1.02) | 1.89 (0.34 to 5.85) | 0.79 (0.17 to 1.86) | 0.75% (-1.4 to 2.95) | 1.84 (0.35 to 5.5) | 0.8 (0.19 to 1.82) | 0.56% (-1.44 to 2.6) |
| South Africa | 202 (51 to 415) | 238 (64 to 483) | 0.18% (0.06 to 0.29) | 0.55 (0.14 to 1.13) | 0.43 (0.12 to 0.87) | -0.64% (-0.84 to -0.43) | 0.55 (0.15 to 1.12) | 0.42 (0.12 to 0.85) | -0.7% (-0.89 to -0.5) |
| South Sudan | 29 (8 to 60) | 63 (14 to 138) | 1.16% (0.67 to 2.27) | 0.5 (0.13 to 1.03) | 0.68 (0.16 to 1.49) | 2.11% (-0.38 to 4.66) | 0.54 (0.15 to 1.1) | 0.7 (0.18 to 1.49) | 1.83% (-0.45 to 4.15) |
| Spain | 363 (84 to 728) | 395 (103 to 812) | 0.09% (-0.02 to 0.27) | 0.94 (0.22 to 1.88) | 0.86 (0.22 to 1.76) | -0.5% (-0.57 to -0.43) | 0.96 (0.22 to 1.94) | 0.95 (0.23 to 2.01) | -0.2% (-0.27 to -0.14) |
| Sri Lanka | 338 (74 to 902) | 123 (31 to 248) | -0.64% (-0.81 to -0.29) | 1.97 (0.43 to 5.24) | 0.56 (0.14 to 1.13) | -3.85% (-5.7 to -1.97) | 1.84 (0.41 to 4.84) | 0.56 (0.14 to 1.14) | -3.61% (-5.47 to -1.72) |
| Sudan | 298 (58 to 823) | 218 (55 to 437) | -0.27% (-0.65 to 0.53) | 1.47 (0.29 to 4.07) | 0.53 (0.13 to 1.07) | -2.02% (-3.22 to -0.8) | 1.39 (0.27 to 3.62) | 0.52 (0.14 to 1.03) | -1.99% (-3.15 to -0.82) |
| Suriname | 2 (0 to 3) | 3 (1 to 5) | 0.62% (0.53 to 0.74) | 0.44 (0.1 to 0.88) | 0.48 (0.12 to 0.95) | 0.3% (0.25 to 0.34) | 0.42 (0.1 to 0.83) | 0.49 (0.12 to 0.97) | 0.53% (0.48 to 0.58) |
| Sweden | 99 (31 to 194) | 124 (42 to 248) | 0.25% (0.18 to 0.37) | 1.15 (0.36 to 2.25) | 1.21 (0.41 to 2.43) | 0.13% (0.08 to 0.19) | 1.09 (0.31 to 2.23) | 1.11 (0.31 to 2.22) | 0% (-0.11 to 0.1) |
| Switzerland | 105 (26 to 213) | 110 (30 to 223) | 0.05% (-0.02 to 0.15) | 1.53 (0.38 to 3.11) | 1.25 (0.34 to 2.54) | -1.07% (-1.2 to -0.94) | 1.5 (0.36 to 3.1) | 1.27 (0.31 to 2.7) | -0.98% (-1.16 to -0.8) |
| Syrian Arab Republic | 69 (14 to 141) | 197 (47 to 468) | 1.87% (0.75 to 4.28) | 0.53 (0.11 to 1.09) | 1.36 (0.32 to 3.23) | 10.16% (6.57 to 13.88) | 0.48 (0.11 to 0.96) | 1.58 (0.37 to 3.75) | 10.8% (7.15 to 14.58) |
| Taiwan (Province of China) | 90 (22 to 185) | 72 (20 to 143) | -0.2% (-0.27 to -0.05) | 0.44 (0.11 to 0.91) | 0.31 (0.08 to 0.61) | -1.8% (-2.13 to -1.47) | 0.43 (0.11 to 0.88) | 0.28 (0.07 to 0.56) | -2.06% (-2.4 to -1.72) |
| Tajikistan | 49 (11 to 100) | 72 (16 to 146) | 0.46% (0.39 to 0.54) | 0.91 (0.2 to 1.86) | 0.76 (0.17 to 1.53) | -3.62% (-5.53 to -1.67) | 0.86 (0.19 to 1.72) | 0.71 (0.16 to 1.43) | -3.56% (-5.44 to -1.63) |
| Thailand | 283 (67 to 566) | 327 (84 to 647) | 0.16% (0.04 to 0.32) | 0.5 (0.12 to 1) | 0.47 (0.12 to 0.92) | -0.26% (-0.48 to -0.04) | 0.48 (0.12 to 0.96) | 0.46 (0.11 to 0.93) | -0.15% (-0.37 to 0.07) |
| Timor-Leste | 12 (3 to 32) | 5 (1 to 10) | -0.59% (-0.82 to -0.1) | 1.5 (0.32 to 4.14) | 0.36 (0.08 to 0.73) | -7.63% (-9.96 to -5.23) | 1.33 (0.29 to 3.62) | 0.36 (0.09 to 0.71) | -7.17% (-9.43 to -4.85) |
| Togo | 15 (4 to 30) | 33 (9 to 68) | 1.25% (1.15 to 1.38) | 0.41 (0.1 to 0.81) | 0.42 (0.11 to 0.86) | -0.02% (-0.5 to 0.48) | 0.44 (0.12 to 0.9) | 0.45 (0.13 to 0.93) | -0.01% (-0.44 to 0.42) |
| Tokelau | 0 (0 to 0) | 0 (0 to 0) | -0.01% (-0.08 to 0.08) | 0.35 (0.09 to 0.71) | 0.41 (0.11 to 0.85) | 0.61% (0.53 to 0.68) | 0.37 (0.1 to 0.76) | 0.43 (0.12 to 0.87) | 0.49% (0.4 to 0.59) |
| Tonga | 0 (0 to 1) | 0 (0 to 1) | 0.12% (0.02 to 0.22) | 0.32 (0.07 to 0.64) | 0.34 (0.08 to 0.7) | 0.26% (-0.06 to 0.59) | 0.32 (0.08 to 0.62) | 0.33 (0.08 to 0.69) | 0.23% (-0.1 to 0.57) |
| Trinidad and Tobago | 7 (2 to 13) | 6 (1 to 12) | -0.07% (-0.26 to 0.08) | 0.55 (0.13 to 1.1) | 0.44 (0.11 to 0.88) | -0.4% (-0.55 to -0.26) | 0.53 (0.13 to 1.05) | 0.47 (0.11 to 0.95) | -0.03% (-0.19 to 0.13) |
| Tunisia | 45 (10 to 92) | 63 (15 to 126) | 0.39% (0.29 to 0.51) | 0.54 (0.12 to 1.09) | 0.54 (0.13 to 1.09) | 0.16% (0.08 to 0.23) | 0.51 (0.12 to 1.05) | 0.55 (0.13 to 1.12) | 0.38% (0.29 to 0.46) |
| Turkey | 332 (74 to 659) | 468 (113 to 971) | 0.41% (0.21 to 0.65) | 0.56 (0.12 to 1.1) | 0.58 (0.14 to 1.19) | -0.51% (-1.31 to 0.3) | 0.52 (0.12 to 1.03) | 0.58 (0.14 to 1.2) | -0.26% (-1.06 to 0.55) |
| Turkmenistan | 29 (6 to 58) | 39 (9 to 81) | 0.36% (0.3 to 0.44) | 0.78 (0.17 to 1.58) | 0.77 (0.17 to 1.59) | -0.45% (-0.87 to -0.04) | 0.71 (0.16 to 1.43) | 0.75 (0.17 to 1.54) | -0.26% (-0.73 to 0.22) |
| Tuvalu | 0 (0 to 0) | 0 (0 to 0) | 0.5% (0.41 to 0.63) | 0.36 (0.09 to 0.74) | 0.43 (0.11 to 0.87) | 0.55% (0.44 to 0.66) | 0.37 (0.1 to 0.75) | 0.43 (0.12 to 0.86) | 0.44% (0.35 to 0.52) |
| Uganda | 111 (25 to 248) | 192 (48 to 391) | 0.74% (0.15 to 1.28) | 0.64 (0.14 to 1.43) | 0.47 (0.12 to 0.95) | -1.09% (-1.95 to -0.23) | 0.64 (0.16 to 1.37) | 0.48 (0.14 to 0.97) | -0.99% (-1.75 to -0.23) |
| Ukraine | 880 (213 to 1798) | 598 (145 to 1211) | -0.32% (-0.35 to -0.28) | 1.67 (0.4 to 3.41) | 1.36 (0.33 to 2.75) | -0.9% (-1.1 to -0.7) | 1.71 (0.4 to 3.5) | 1.45 (0.33 to 2.96) | -0.79% (-1.04 to -0.54) |
| United Arab Emirates | 13 (3 to 27) | 64 (15 to 129) | 3.86% (3.51 to 4.34) | 0.71 (0.16 to 1.42) | 0.7 (0.16 to 1.4) | 0.05% (-0.12 to 0.21) | 0.65 (0.15 to 1.31) | 0.64 (0.15 to 1.29) | -0.15% (-0.21 to -0.09) |
| United Kingdom | 527 (130 to 1086) | 629 (164 to 1287) | 0.19% (0.1 to 0.32) | 0.92 (0.23 to 1.89) | 0.94 (0.24 to 1.91) | 0.06% (-0.02 to 0.14) | 0.97 (0.23 to 2.02) | 0.97 (0.24 to 2.03) | -0.04% (-0.13 to 0.06) |
| United Republic of Tanzania | 119 (31 to 243) | 262 (68 to 532) | 1.19% (1.11 to 1.29) | 0.46 (0.12 to 0.94) | 0.46 (0.12 to 0.94) | 0.02% (-0.1 to 0.14) | 0.48 (0.13 to 0.97) | 0.48 (0.13 to 0.96) | 0% (-0.1 to 0.1) |
| United States of America | 2835 (811 to 5679) | 3630 (1134 to 7096) | 0.28% (0.17 to 0.52) | 1.12 (0.32 to 2.24) | 1.11 (0.35 to 2.16) | -0.27% (-0.53 to -0.01) | 1.1 (0.31 to 2.23) | 1 (0.29 to 2.02) | -0.72% (-1.02 to -0.42) |
| United States Virgin Islands | 1 (0 to 1) | 1 (0 to 1) | 0.02% (-0.07 to 0.18) | 0.55 (0.14 to 1.11) | 0.57 (0.17 to 1.14) | 0.01% (-0.11 to 0.14) | 0.55 (0.15 to 1.12) | 0.59 (0.16 to 1.2) | 0.08% (-0.07 to 0.23) |
| Uruguay | 35 (8 to 71) | 34 (8 to 69) | -0.03% (-0.12 to 0.06) | 1.11 (0.26 to 2.26) | 0.98 (0.23 to 2) | -0.6% (-0.7 to -0.51) | 1.13 (0.26 to 2.3) | 1.02 (0.24 to 2.11) | -0.53% (-0.63 to -0.42) |
| Uzbekistan | 190 (41 to 384) | 278 (62 to 563) | 0.47% (0.39 to 0.55) | 0.91 (0.19 to 1.83) | 0.83 (0.18 to 1.67) | -0.49% (-0.62 to -0.36) | 0.84 (0.18 to 1.69) | 0.79 (0.18 to 1.59) | -0.39% (-0.49 to -0.29) |
| Vanuatu | 0 (0 to 1) | 1 (0 to 2) | 1.17% (1.04 to 1.32) | 0.31 (0.07 to 0.62) | 0.34 (0.08 to 0.71) | -0.26% (-1.43 to 0.93) | 0.3 (0.07 to 0.6) | 0.34 (0.08 to 0.68) | -0.3% (-1.49 to 0.91) |
| Venezuela (Bolivarian Republic of) | 140 (32 to 285) | 175 (42 to 359) | 0.25% (0.18 to 0.35) | 0.74 (0.17 to 1.51) | 0.62 (0.15 to 1.28) | -0.89% (-1.72 to -0.06) | 0.69 (0.16 to 1.39) | 0.65 (0.15 to 1.34) | -0.53% (-1.37 to 0.32) |
| Viet Nam | 271 (65 to 547) | 457 (118 to 906) | 0.68% (0.52 to 0.9) | 0.4 (0.09 to 0.81) | 0.47 (0.12 to 0.94) | 0.51% (0.38 to 0.64) | 0.41 (0.1 to 0.8) | 0.48 (0.12 to 0.95) | 0.43% (0.29 to 0.57) |
| Yemen | 71 (15 to 143) | 739 (160 to 2039) | 9.34% (3.59 to 23.5) | 0.52 (0.11 to 1.04) | 2.35 (0.51 to 6.47) | 4.91% (3.13 to 6.73) | 0.5 (0.12 to 1) | 2.07 (0.45 to 5.69) | 4.84% (3.04 to 6.68) |
| Zambia | 35 (9 to 71) | 80 (21 to 164) | 1.28% (1.06 to 1.48) | 0.44 (0.11 to 0.9) | 0.44 (0.11 to 0.9) | 0.12% (0 to 0.24) | 0.47 (0.13 to 0.95) | 0.47 (0.13 to 0.94) | 0.08% (-0.01 to 0.17) |
| Zimbabwe | 38 (10 to 76) | 63 (16 to 126) | 0.65% (0.47 to 1.04) | 0.37 (0.09 to 0.74) | 0.42 (0.11 to 0.84) | 0.07% (-0.21 to 0.35) | 0.39 (0.11 to 0.78) | 0.43 (0.12 to 0.85) | -0.01% (-0.27 to 0.24) |

YLDs, years lived with disability; CR, crude rate; ASR, age-standardized rate; EAPC, estimated annual percentage change; UI, uncertainty interval; CI, confidence interval.
